# Supplementary material for: Differences in risk factors associated with single and multiple concurrent forms of undernutrition (stunting, wasting or underweight) among children under 5 in Bangladesh: a nationally representative cross-sectional study
Source: BMJ Open. 2021 Dec 11;11(12):e052814. doi: 10.1136/bmjopen-2021-052814 (PMC8672009; doi:10.1136/bmjopen-2021-052814)
Supplement: Supplementary data [file bmjopen-2021-052814supp001.pdf]

Supplementary Table 1

Definitions of outcome variables

Variable	Type of variable	Description	Measurement	Scale of measurement	Number of responses/Mis sing
**Multiple concurrent forms of undernutrition**	**Outcome variable**	A child was considered to have multiple concurrent forms of undernutrition when stunting and underweight and wasting and underweight, or these three forms of undernutrition (stunting, wasting, and underweight) are present in the same child (Figure 2)	No, yes	Binary	7,806/96
**Single form of undernutrition**	**Outcome variable**	A child with a single form of undernutrition (in a composite manner) includes a child is living with stunting, wasting, or underweight only (Figure 2)	No, yes	Binary	7,806/96

Supplementary Table 2

Estimating scores for a child with different nutritional status

**Nutritional indicators**	**Code**	**Stunting**	**wasting**	**underweight**	**Total score**
		**yes (1)/no (0)**	**yes (1)/no (0)**	**yes (1)/no (0)**	
**Stunning**					
No	0				
Yes	1				
**Wasting**					
No	0				
Yes	1				
**Underweight**					
No	0				
Yes	1				
**Healthy children/no undernutrition**		0	0	0	0
**Only stunting**		1	0	0	1
**Only wasting**		0	1	0	1
**Only underweight**		0	0	1	1
**Stunting & wasting**		1	0	1	2
**Stunting & underweight**		1	0	1	2
**Wasting and underweight**		0	1	1	2
**Stunting, wasting and underweight**		1	1	1	3

Supplementary Table 3

Variable definitions (parental characteristics)

Variable	Type of variable	Description	Measurement	Scale of measurement	Number of responses/Mis sing
**Mothers' age (in years)**	Independent variable	Age of mothers at the time of data collection	15-19, 20-24, 25-29, 30-34, 35-39, ≥ 40	Discrete, categorial	7,902/0
**Parents' education**	Independent variable	Parental educational status	Both parents were uneducated, only father was uneducated, only mother was uneducated, both parents were educated	Categorical	7,779/123
**Mother currently working**	Independent variable	Mothers engaged in economic activity at the time of data collection	No, yes	Binary	7,902/0
**Underweight mother**	Independent variable	Mother with <18.5 kg/m^2 of body mass index	No, yes	Binary	7,902/0
**Mothers received antenatal care**	Independent variable	Medical surveillance and review performed during pregnancy for the early detection of possible complications of pregnancy	No, yes	Binary	4,540/3,562
**Mothers received postnatal care**	Independent variable	Postnatal care is the individualised care provided to meet the needs of a mother and her baby following childbirth	No, yes	Binary	4,535/3,367
**Mothers' experience inmate partner violence**	Independent variable	In the BDHS surveys, IPV was measured using a shortened and modified version of the Conflict Tactics Scale and based on information such as physical violence and sexual violence. Physical violence included a wife being beaten if she went out without telling her partner, if she neglected the children and if she ever argued with her partner. The incidence of sexual violence was calculated on a woman's positive response to a question asking whether she had ever been physically forced to	No, yes	Binary	7,902/0

		have sexual intercourse even when she did not want to. For each of the questions, responses were re-coded dichotomously: 1= yes and 0 = no and IPV scores were calculated through adding up all four binary IPV indicators with maximum and minimum values of 4 and 0 respectively. These estimated scores were then recoded as a binary variable with categories labeled no IPV=0 and yes=1 (score 1- 4).			
**Mothers' decision-making autonomy**	Independent variable	in the BDHS surveys, a woman's decision-making power is assessed on the following three themes: 1) a woman who usually decides on her healthcare 2) a woman who usually decides on large household purchases and 3) a woman who usually decides on visits to family or relatives. The response options were as follows: (a) respondent alone, (b) respondent and husband/partner, (c) respondent and another person, (d) husband/partner alone, (e) someone else, (f) other. For each question, a value of 1 was assigned for inability in decision-making if the responses were d, e, or f and 0 for otherwise if the responses were a, b, or c. The values were then added, resulting in a score from 0 to 3. The Cronbach's α for the instruments was 0·79, indicating high internal consistency.	Not practiced, Practiced	Binary	7,778/124
**Father's occupation**	Independent variable	Fathers engaged in economic activity at the time of data collection	Currently not working, manual labourer, professional, businessman	Categorical	7,772/130

Supplementary Table 4

Variable definitions (household and contextual factors)

Variable	Type of variable	Description	Measurement	Scale of measurement	Number of responses/Mis sing
**Source of water**	Independent variable	improved: piped into dwelling, piped to yard/plot, public tap/standpipe, piped to neighbor, tube well or borehole, protected well, protected spring, rainwater, tanker truck, cart with small tank, bottled water; unimproved: unprotected well, unprotected spring, surface water (river, dam, lake, pond, stream, canal, irrigation channel), other	Improved, unimproved	Binary	7,902/0
**Type of toilet facility**	Independent variable	improved: flush - to piped sewer system, flush - to septic tank, flush - to pit latrine, flush - don't know where, pit latrine - ventilated improved pit, pit latrine - with slab, composting toilet; unimproved: flush - to somewhere else, pit latrine - without slab / open pit, bucket toilet, hanging toilet/latrine, other	Improved, unimproved	Binary	7,902/0
**Solid waste use in cooking**	Independent variable	solid waste includes coal, lignite, charcoal, wood, straw / shrubs / grass, agricultural crop and animal dung; unsolid include electricity, natural gas, processed gas, biogas, kerosene	No, yes	Binary	7,896/8
**Mass media exposure**	Independent variable	mass media exposure through television, radio and newspaper/magazine has been defined as exposure to at least one media that exposes to at least once a week	No, yes	Binary	7,902/0
**Wealth index**	Independent variable	wealth index in the DHS surveys is calculated, by the DHS authority, based on information on household characteristics and assets using	Poorest, poorer, middle, richer, richest	Categorical	7,902/0

		principal component analysis. Then households are classified into quintiles based on the values of the wealth index, where households with lower values of the index is considered as poorest and vice-versa			
**Place of residence**	Independent variable		Urban, rural	Binary	7,902/0

Supplementary Table 5

Variable definitions (child characteristics)

Variable	Type of variable	Description	Measurement	Scale of measurement	Number of responses/Mis sing
**Children's age (in months)**	Independent variable	Age of the children at the time of data collection	0-11 months, 12-23 months, 24-35 months, 36-47 months, 48-59 months	Categorical	7,902/0
**Sex of child**	Independent variable	Sex differential of children	Male, female	Binary	7,902/0
**Birth order**	Independent variable	Birth order is the chronological order of sibling births in a family	One, two, three, four and above	Categorical	7,902/0
**Low birth weight**	Independent variable	Children were <2.5 kg of weight during birth. Approximately 75% mothers can correctly report their baby's size at birth; therefore mother's recall is a valid proxy measure of birth weight.	No, yes, not weighted	Categorical	4,735/3,167
**Currently had disease**	Independent variable	Children had at least cough, fever or diarrhea before 2 weeks of the survey	No, yes	Binary	7,899/3
