# Supplementary material for: Differences in risk factors associated with single and multiple concurrent forms of undernutrition (stunting, wasting or underweight) among children under 5 in Bangladesh: a nationally representative cross-sectional study
Source: BMJ Open. 2021 Dec 11;11(12):e052814. doi: 10.1136/bmjopen-2021-052814 (PMC8672009; doi:10.1136/bmjopen-2021-052814)
Supplement: Author's manuscript [file bmjopen-2021-052814.draft_revisions.pdf]

BMJ Open is committed to open peer review. As part of this commitment we make the peer review history of every article we publish publicly available.

When an article is published we post the peer reviewers' comments and the authors' responses online. We also post the versions of the paper that were used during peer review. These are the versions that the peer review comments apply to.

The versions of the paper that follow are the versions that were submitted during the peer review process. They are not the versions of record or the final published versions. They should not be cited or distributed as the published version of this manuscript.

BMJ Open is an open access journal and the full, final, typeset and author-corrected version of record of the manuscript is available on our site with no access controls, subscription charges or pay-per-view fees (http://bmjopen.bmj.com).

If you have any questions on BMJ Open's open peer review process please email info.bmjopen@bmj.com

BMJ Open

Differences in risk factors between cooccurrence and the single dimension of undernutrition among under-5 children in Bangladesh

Journal:	*BMJ Open*
Manuscript ID	bmjopen-2021-052814
Article Type:	Original research
Date Submitted by the Author:	27-Apr-2021
Complete List of Authors:	Chowdhury, Mohammad ; First Capital University of Bangladesh, Department of Public Health<br>Khan, Hafiz; University of West London, College of Nursing, Midwifery and Healthcare<br>Rashid, Mamunur; University of Gävle, Department of Public Health and Sports Sciences<br>Kabir, Russell; Anglia Ruskin University<br>Islam, Sazin; First Capital University of Bangladesh, Department of Public Health<br>Shariful Islam, Md.; First Capital University of Bangladesh, Department of Public Health<br>Kader, Manzur; Karolinska Institutet, Institute of Environmental Medicine
Keywords:	Community child health < PAEDIATRICS, NUTRITION & DIETETICS, PUBLIC HEALTH, PREVENTIVE MEDICINE

SCHOLARONE™
Manuscripts

I, the Submitting Author has the right to grant and does grant on behalf of all authors of the Work (as defined in the below author licence), an exclusive licence and/or a non-exclusive licence for contributions from authors who are: i) UK Crown employees; ii) where BMJ has agreed a CC-BY licence shall apply, and/or iii) in accordance with the terms applicable for US Federal Government officers or employees acting as part of their official duties; on a worldwide, perpetual, irrevocable, royalty-free basis to BMJ Publishing Group Ltd ("BMJ") its licensees and where the relevant Journal is co-owned by BMJ to the co-owners of the Journal, to publish the Work in this journal and any other BMJ products and to exploit all rights, as set out in our licence.

The Submitting Author accepts and understands that any supply made under these terms is made by BMJ to the Submitting Author unless you are acting as an employee on behalf of your employer or a postgraduate student of an affiliated institution which is paying any applicable article publishing charge ("APC") for Open Access articles. Where the Submitting Author wishes to make the Work available on an Open Access basis (and intends to pay the relevant APC), the terms of reuse of such Open Access shall be governed by a Creative Commons licence – details of these licences and which Creative Commons licence will apply to this Work are set out in our licence referred to above.

Other than as permitted in any relevant BMJ Author's Self Archiving Policies, I confirm this Work has not been accepted for publication elsewhere, is not being considered for publication elsewhere and does not duplicate material already published. I confirm all authors consent to publication of this Work and authorise the granting of this licence.

For peer review only

Differences in risk factors between cooccurrence and the single dimension of undernutrition among under-5 children in Bangladesh

Mohammad Rocky Khan Chowdhury[1], Hafiz TA Khan[2], Mamunur Rashid[3], Russell Kabir[4], Sazin Islam[1], Md. Shariful Islam[1], Manzur Kader[5]

[1]Department of Public Health, First Capital University of Bangladesh, Chuadanga, Bangladesh

[2]College of Nursing, Midwifery and Healthcare University of West London, London, United Kingdom

[3]Department of Public Health and Sports Sciences, University of Gävle, Sweden

[4]Senior lecturer, School of Allied Health, Faculty of Health, Education, Medicine and Social Care, Anglia Ruskin University, Chelmsford, Essex, United Kingdom

[1]Department of Public Health, First Capital University of Bangladesh, Chuadanga, Bangladesh

[1]Department of Public Health, First Capital University of Bangladesh, Chuadanga, Bangladesh

[5]Institute of Environmental Medicine, Karolinska Institutet, Stockholm, Sweden

*Correspondence to

Dr. Manzur Kader, Ph.D.

Unit of Occupational Medicine,

Institute of Environmental Medicine | Karolinska Institute

Solnavägen 4, Torsplan floor 10, 113 65 Stockholm

Phone: +46 768 53 98 67 | manzur.kader@ki.se

For peer review only - http://bmjopen.bmj.com/site/about/guidelines.xhtml

Abstract

Objectives: The current study aims to differentiate the risk factors of cooccurrence and the single dimension of undernutrition among under-5 children in Bangladesh.

Design, setting, and participants: This cross-sectional study analyzed data from the 2017-18 Bangladesh Demographic and Health Survey (BDHS), a nationally representative sample of 7,661 children under-5.

Outcome measure: A child is considered to have a cooccurrence of undernutrition if he/she has either coexistence of stunting and underweight; wasting and underweight at the same time or the coexistence of stunting, wasting, and underweight. Moreover, a child with a single dimension of undernutrition includes having stunting, wasting, and underweight independently.

Statistical analysis: A Chi-square test was used to assess the prevalence of undernutrition. Odds ratio (OR) and confidence interval (CI) of potential risk factors were quantified using logistic regression analysis.

Results: Two out of five under-5 children are suffering undernutrition. The prevalence of cooccurrence and the single dimension of child undernutrition were 19.3% and 18.9% respectively. The key risk factors of cooccurrence of undernutrition were children born with low birth weight [OR-3.40, 95% CI-2.52, 4.57], socio-economically poorest households [OR-2.29, 95% CI-1.74, 3.01] and children age group 48-59 months [OR-2.18, 95% CI-1.80, 2.63], on the other hand, children age group 12-23 months [AOR-161, 95% CI-1.35, 1.92], socio-economically poorer households [OR-1.41, 95% CI-1.09, 1.82] and paternal illiteracy [OR-1.19, 95% CI-1.01, 1.42] was significantly associated with a single dimension of undernutrition.

Conclusion: One-fifth of the children are suffering cooccurrence of undernutrition and this is similar as measured by the single dimension of undernutrition. Parental education, mother's poor nutritional status, children's age, birth order, and low birth weight are the main differentiating risk factors of cooccurrence and the single dimension of undernutrition in Bangladesh which should be taken into consideration to formulate an evidence-based strategy to reduce undernutrition among under-5 children.

Keywords: Children, malnutrition, prevalence, determinant, coexistence, and Bangladesh

For peer review only - http://bmjopen.bmj.com/site/about/guidelines.xhtml

Strengths and limitations of this study

To the best of our knowledge, this is the first study that differentiate the single dimension and occurrence of under-5 child undernutrition in Bangladesh using a nationally representative, probability-based cross-sectional sample survey.

The study considered a wide range of variables to assess the risk factors of both the single dimension and occurrence of undernutrition by adopting a robust analytical method.

Parental education, mothers with poor nutritional status, father's employment status, children's age, birth order, and low birth weight differentiate the risk assessment between cooccurrence and the single dimension of undernutrition.

The study sample does not allow to establish causation.

For peer review only

For peer review only - http://bmjopen.bmj.com/site/about/guidelines.xhtml

Word count: 3309 words

Introduction

Worldwide, approximately 195 million under-5 children, are suffering from undernutrition in form of stunting, wasting, and underweight; of which 3.1 million children death occur due to these childhood conditions.[1,2] Globally, 124 countries have the burden of coexistence of undernutrition and of which 29% (41 countries) are struggling with high levels of all three conditions of undernutrition, which leads to a 12-fold elevated risk of mortality than usual.[3,4] Also, globally around 3.62% of children aged under five years have coexistence of stunting and wasting.[4] Undernutrition reduces the immunological capacity of a child to defend against diseases and recurrent infections, such as diarrhea, pneumonia, malaria, measles, and AIDS, which leads to an inconsistent growth and development of a child both mentally and physically.[5,6]

Child undernutrition is one of the global health problems, particularly in low-and middle-income countries including Bangladesh. Despite child undernutrition has been declined over the years, rates of child malnutrition are still among the highest in the world.[4] According to recent estimates, around 40% of children under-5 are suffering from undernutrition in Bangladesh.[7] Previous studies conducted in Bangladesh have been shown that child undernutrition occurs from several risk factors such as poor maternal education, lower socioeconomic status, low birth weight, poor infant and young child feeding practices, frequent infections, and inadequate access to health care and water and sanitation.[5,8,9] These previous studies identified the risk factors of undernutrition using conventional disaggregated indicators/conditions (such as stunting, wasting, and/or underweight). These indicators of undernutrition are not sufficient to convey the credible estimates of its determinants as many children are suffering from duel or triple conditions of undernutrition.

For peer review only - http://bmjopen.bmj.com/site/about/guidelines.xhtml

Under-5 child undernutrition and its risk factors is still an issue of deliberation in developing countries, like Bangladesh. More than 30% of children under-5 were reported to have the cooccurrence of undernutrition in Bangladesh, which can differ the magnitude of the prevalence and risk factors other than conventional indicators.[10] Although previous studies in Bangladesh identified several risk factors for child undernutrition, the factors were measured for disaggregated indicators – stunting, wasting, and underweight.[5,8] A previous study also assessed the under-5 child undernutrition using Composite Index of Anthropometric Failure (CIAF), but this method did not differentiate the risk factors of single dimension and cooccurrence of undernutrition.[9] To the best of our knowledge, the prevalence and risk factors of undernutrition among under-5 children living with a single dimension and cooccurrence of undernutrition have not been widely investigated previously in Bangladesh. Factors associated with cooccurrence of undernutrition may not be the same factors for a single dimension of undernutrition as well as their magnitudes. Further, the risk factors measured by disaggregated indicators might differ both the outcomes (single dimension and cooccurrence of undernutrition). Therefore, the present study aims to differentiate the risk factors of cooccurrence and single dimension of undernutrition among under-5 children in Bangladesh using a more recent nationally representative population-based survey. To address the nutritionally more vulnerable children, this study might be useful for policymakers.

Methodology

Data source

After excluding missing information with regards to selected variables, a total of 7,661 children under-5 were born in January 2013 or later were included in this study from Bangladesh Demographic Health Surveys (BDHS) 2017-18 (Figure 1). The BDHS 2017-18

For peer review only - http://bmjopen.bmj.com/site/about/guidelines.xhtml

data is nationally representative with a nearly 99% response rate that includes data from Bangladeshi adults (both male and female) regarding demographic and social characteristics, health and nutritional indicators to monitor a wide range of the population. The data collection was started on 24 October 2017 and finished on 15 March 2018. These surveys were based on multistage stratified sampling techniques of households. At the first stage, 675 primary sampling units (PSUs) (250 PSU from urban and 425 PSU from rural) based on enumeration areas (clusters) from the census survey 2011 designed by the Bangladesh Bureau of Statistics were selected using probability proportional to size technique and a systematic sample of 30 households from each PSU were selected at the final stage using equal probability systematic sampling technique.[7] This multistage sampling technique including its sampling weight helps reducing potential bias. Besides, the survey interviewers interviewed all ever-married women aged 15-49 years from the pre-selected households without replacement and change in the implementing stage to prevent bias. Each BDHS used a standard questionnaire. Details of the survey questionnaire, sample design, data collection procedure, and all other issues are discussed elsewhere.[7]

Outcome variables and operational definitions

The outcomes of the study were the cooccurrence and single dimension of undernutrition among under-5 children. A child was considered to be undernourished or stunted (short stature for age), wasted (dangerously thin) and underweight (low weight for age) respectively if the height-for-age, weight-for-height, and weight-for-age indices were 2 standard deviations (SDs) or more below the respective median of the WHO reference population.[11] A child is considered to have a cooccurrence of undernutrition if he/she has either coexistence of stunting and underweight; wasting and underweight at the same time or the coexistence of

For peer review only - http://bmjopen.bmj.com/site/about/guidelines.xhtml

stunting, wasting, and underweight. Also, a child with a single dimension of undernutrition includes stunting, wasting, or underweight only (Figure 2).

Independent variables

The candidate variables considered for analysis in the present study were based on relevant previous pieces of literature.[5,12-16] These variables include maternal age in years (15-19, 20-24, 25-29, 30-34, 35-39, ≥40); parents' education (both parents with no formal education, only father with no formal education when mother was educated, only mother with no formal education when father was educated, both parents educated); mother's current working status (currently not working, currently working); mother's underweight status ((normal/average (≥18.5 kg/m^2), underweight (<18.5 kg/m^2)); status of mother's antenatal and postnatal care (not received, received); mother's experience of inmate partner violence (IPV) (not experienced, experienced: a wife being beaten by partner if she went out without telling him or/and neglected the children or/and she ever argued with her partner or/and burned food or/and refused to have sex);[7] mothers' decision-making autonomy (not experienced, experienced: a woman who usually decides by herself/jointly with husband at least on her healthcare or on large household purchases or visits to family or relatives);[7] religion (Islam, others); source of drinking water (improved, unimproved),[17] use of solid waste in cooking (solid, non-solid); type of toilet facility (improved, unimproved),[17] mass media exposure (no, yes: expose to either radio, television, newspapers, or magazines at least once a week), wealth index (integrating household asset ownership and access to drinking water and sanitation).[7] Moreover, various factors related to children, such as age, birth order, and birth weight status,[18-20] recent morbidity status (the child had at least one morbid condition out of diarrhea, fever, and cough in two weeks preceding the survey)[7] were included. Detailed variables' scale of measurement with missing information used in this study has been clearly defined in online supplemental table 1.

Patient and public involvement

Patients were not involved in the design, conduct or interpretation of the study.

For peer review only - http://bmjopen.bmj.com/site/about/guidelines.xhtml

Statistical analysis

The background characteristics of the children and their parents were calculated using descriptive statistics. Chi-square test (bivariate analysis) and logistic regression analysis were used to determine associations between the selected independent variables and outcomes. All independent variables found significant in bivariate analysis were simultaneously entered into the multiple regression model for adjustment to identify risk factors for cooccurrence and a single dimension of undernutrition. The findings from logistic regression analysis were assessed using odds ratio (OR) and confidence interval (CI). The significance level was set at $p < 0.05$ (2-tailed) in all analyses. The data were analyzed by considering cluster effects using survey weights that represent the whole country, both urban and rural areas, to ensure the precision of the estimates. Stata version 14.2 (StataCorp LP, College Station, Texas) was used for all analyses considering the complex nature of the sampling weight of the BDHS.

Results

Table 1 shows that 7% of mothers of children had no education, 15% of mothers were reported underweight, more than 90% of mothers of children received antenatal care and one-third of them (33%) did not receive postnatal care, and 18% of mothers experienced IPV. Around 42% of children were from poor households and 66% were living in rural areas. Parallel percentage distribution of the children (around 20%) was observed among the various age categories and 51% of children were male (Table 1).

Prevalence of cooccurrence and single dimension of undernutrition

For peer review only - http://bmjopen.bmj.com/site/about/guidelines.xhtml

In Bangladesh, the overall prevalence of child undernutrition was 38%. The prevalence of cooccurrence of undernutrition and single dimension of undernutrition among under-5 children was approximately estimated at 19%. Further, the prevalence of cooccurrence of undernutrition was significantly higher among children of parents with no formal education (37%), children of underweight mothers (approximately 30%), children with fathers who were manual labour (28%), children were ≥ 4 birth order (approximately 28%), and children born with low birth weight (27%) (Table 2).

The single dimension of undernutrition was highly prevalent among children of a father with no formal education (when mothers are educated) (23%), children of age group 12-35 months (approximately 23%), those from the poorest section of the society (22%), children of underweight mothers and households had no mass media exposure (21%), children of fathers who were manual labourer and households with unimproved toilet facility (approximately 21%) and children born with low birth weight (20%) (Table 2).

Risk factors

The key risk factors of cooccurrence of undernutrition were children born with low birth weight (vs healthy weight) [AOR-3.40, 95% CI-2.52, 4.57]; children of socio-economically poorest households (vs richest) [AOR-2.29, 95% CI-1.74, 3.01]; children age group 48-59 months (vs children age group 0-11 months) [AOR-2.18, 95% CI-1.80, 2.63]; children with fathers who are currently unemployed (vs businessman fathers) [AOR-1.84, 95% CI-1.07, 3.16] and mothers being underweight (vs healthy) [AOR-1.78, 95% CI-153, 2.06] (Table 3).

In terms of a single dimension, children age group 12-23 months (vs children age group 0-11 months) [AOR-1.61, 95% CI-1.35, 1.92]; children of socio-economically poorest households (vs richest) [AOR-1.41, 95% CI-1.09, 1.82]; children of fathers with no formal education

For peer review only - http://bmjopen.bmj.com/site/about/guidelines.xhtml

when mothers are educated (vs both parents educated) [AOR-1.19, 95% CI-1.01, 1.42] and children of fathers who are manual labourer (vs businessman fathers) [AOR-1.17, 95% CI-1.01, 1.36] were identified as key risk factors of single dimension of undernutrition (Table 3).

Discussion

The prevalence of cooccurrence and single dimension of undernutrition among under-5 children in Bangladesh was nearly equal which accounts for 19%. This is a bit worrying figure when comparing it with countries, such as Yemen, India, Ethiopia, Malawi, and Argentina where the prevalence of cooccurrence of undernutrition among under-5 children were successively 48%, 39%, 26%, 12%, and 2%; and the single dimension of undernutrition were respectively 21%, 24%, 23%, 39%, and 5%.[21-25] Bangladesh has not still achieved sustainable improvement in reducing child undernutrition as one-fifth of the children are suffering from both conditions of undernutrition. One of the possible reasons could be that poor understanding of the multisectoral approach, lack of coordination among key government sectors, such as health, agriculture, education, urban and local development to address the issue of undernutrition, and poor linkage between key institutions, for example, government institutions, academic, research and training institutions, and national/international non-governmental organizations.[26]

This study reveals that children of mothers with no formal education are living in less protective nature in Bangladesh. Four out of ten children of mothers with no formal education experience a cooccurrence of undernutrition. Further, children born with low birth weight had a 240% higher risk of having cooccurrence of undernutrition. Also, three out of ten children born with low birth weight experience cooccurrence of undernutrition. Previous studies in Bangladesh, Pakistan, Nepal, Malawi, Mexico, and Iran also reported children born with low

For peer review only - http://bmjopen.bmj.com/site/about/guidelines.xhtml

birth weight were more likely to explore malnutrition regardless of cooccurrence and single dimension.[1,27-30] Children born with low birth weight generally increase their height and weight by small increments .[31] Thus, they may remain shorter and lighter and might be severely malnourished without adequate nutrition support. Children with low birth weights are often born to mothers with no formal education and underweight status.[32,33] Maternal/parental illiteracy is associated with poor maternal healthcare access and caregiving to children often leads to an adverse nutritional outcome for mothers and children.[34] This study shows that children of mothers with no formal education and underweight mothers were 1.78 and 1.51 times more likely to experience cooccurrence of undernutrition. A numerous study assessed these factors as key determinants of child undernutrition using disaggregated indicators and need more assessment to estimate the magnitude of risks in case of occurrence and the single dimension of undernutrition.[5,8]

Other key risk factors of cooccurrence of undernutrition include children of socio-economically poorest households, children age group 48-59 months, children of fathers who are currently unemployed that differs by the single dimension of undernutrition to some extent additionally with children age group 12-23 months, children of fathers with no formal education (when mothers are educated) and children of fathers who are labourers. An assessment of the risk difference between parents with formal education and no formal education, and the richest and poorest socio-economic status for cooccurrence and the single dimension of undernutrition indicates higher educational and wealth inequalities for cooccurrence of undernutrition. Further, level of maternal education and economic hardship are not decidedly responsible for suffering from a single dimension of undernutrition among under-5 children. Earlier studies in Bangladesh and other South Asian countries highlighted parental education and wealth index as key risk factors of child undernutrition regardless of cooccurrence and single dimension, but the studies need more precise estimation that could

For peer review only - http://bmjopen.bmj.com/site/about/guidelines.xhtml

For peer review only

help policymakers to introduce context-specific actions for reducing undernutrition.[5,8,14,16,35] Women who have low social status and less education have very limited influence on household decision-making that might influence child nutritional status. On the other hand, a large community in Bangladesh are concerned with wider issues of chronic poverty and this would not reflect the improvement of undernutrition at a community level.[36] To reduce under-5 child undernutrition, increasing the participation of women and girls in education, especially, in rural areas, minimizing the socio-economic inequality by rising the income-generating sectors, and strengthening the nutrition-specific health programs are recommended.

Children of age group 48-59 months had 218% higher risk of having cooccurrence of undernutrition, while children of age group 12-23 months were 61% more likely of having a single dimension of undernutrition. Age has been consistently addressed by many studies and older children were reported to have a higher risk of being undernourished in Bangladesh, Nepal, Pakistan, Ethiopia, and Congo without classifying the two conditions.[12-16] However, in India, younger children had a higher likelihood of being undernourished.[37] Older children are less protective for adverse nutritional outcomes due to poor per capita food distribution among family members in large households and allocate less amount of household foods inequitably meet age-specific energy requirement to young and older children.[38] The risk of poor parental education on cooccurrence of undernutrition differs by the risk of paternal illiteracy (when mothers are educated) on the single dimension of undernutrition in identifying the impact of education on undernutrition. This study shows that paternal education was associated with the single dimension of undernutrition among under-5 children in Bangladesh. Evidence suggests that the impact of paternal education on undernutrition has not been widely addressed; alternatively, maternal education has larger effects on child undernutrition than paternal education.[39] Parental education is associated with positive

For peer review only - http://bmjopen.bmj.com/site/about/guidelines.xhtml

attitudes and behaviors that can reduce the incidence of child undernutrition to some extent. This helps to understand the complex nature of modern health care messages and behavioral changes are not answered by just the most basic literacy and numeracy skills of mothers.[39] Reducing the gender gap for the enrolment in universal upper secondary education may help in tackling the burden of all levels of undernutrition.

This study also indicates that children of unemployed fathers were more likely to experience cooccurrence of undernutrition; conversely, children of fathers who were labourers were less protective in case of a single dimension of undernutrition. Additionally, three out of ten children experience cooccurrence of undernutrition and it was 21% for the single dimension. The effect of paternal unemployment on child undernutrition has not been widely explained, except in a study conducted in China and the study found a significant impact of parental unemployment on children undernutrition.[40] On the other hand, children of fathers having low-paid jobs (labourers, farmers, etc.) have a greater risk of suffering undernutrition.[8,10] A loss of employment or low-paid job is typically associated with lower income available for spending on market goods, including health care, non-household childcare, and nutritious consumption.[40] Increases in home-produced and time-intensive health investments might be some suggestions to tackle undernutrition.

The study analyzed the data of the national survey, the findings can apply to the whole country, and to the low- and middle-income countries whose socio-demographic characteristics and healthcare setting is like Bangladesh. Another strength was to perform appropriate application of statistical tools that might help in understanding the findings. Besides, the multistage sampling technique including its sampling weight helps reducing potential bias. This study was not free from limitations. The cross-sectional nature of the data did not establish a causal relationship between risk factors and outcome variables. This study

For peer review only - http://bmjopen.bmj.com/site/about/guidelines.xhtml

did not control other important indicators, such as the diet pattern of the child, behavioral factors of parents, immunization, and ethnicity due to lack of availability and missing values across the surveys. Another limitation may arise by involving recall bias or information bias, which may result from collecting information about self-reporting age, education, occupation, and household assets, etc.

Conclusion

In Bangladesh, one out of five under-5 children is suffering cooccurrence of undernutrition and a single dimension of undernutrition. Children born with low birth weight, poorest socioeconomic status, and children age group 48-59 months were identified as key risk factors of cooccurrence of undernutrition. On the other hand, children aged group 12-23 months, paternal illiteracy had significant effects on a single dimension of undernutrition. Parental education, underweight mothers, paternal employment status, age of children, birth order, and low birth weight are the main differentiating risk factors of cooccurrence and the single dimension of undernutrition among under-5 children in Bangladesh. The findings highlight factors that should be taken into consideration to formulate an evidence-based strategy to reduce undernutrition among under-5 children. Besides, concerted efforts are essential in making strong collaborations among multiple sectors, such as, government, non-government, educational, social, cultural, and religious institutions to improve nutritional status. The findings from this study may be recommended for low- and middle-income countries. However, further study is warranted in this regard.

For peer review only - http://bmjopen.bmj.com/site/about/guidelines.xhtml

Ethics Approval:

No ethical approval was needed for this study because this study was based on secondary analysis of the data obtained from the Bangladesh Demographic and Health Survey (BDHSs), 2017-2018, The BDHSs were reviewed and approved by the ICF Macro Institutional Review Board (USA), which complies with all of the requirements of 45 CFR 46 "Protection of Human Subjects". The Bangladesh DHS was also reviewed and approved by the National Research Ethics Committee of the Bangladesh Medical Research Council (Dhaka, Bangladesh). The survey ensured international ethical standards of confidentiality, anonymity, and informed consent. However, a request to access datasets from the measure DHS website is made, and the websites have allowed the same before analyses are made.

Consent for publication: Not applicable.

Acknowledgement: The authors thank the MEASURE DHS project for their support and free access to the original data and the Department of Public Health, First Capital University of Bangladesh and for enabling the scope of the research.

Funding: This research received no specific grant from any institutions.

Competing interests: The authors declare that they have no competing interests.

Authors' contribution: MRKC conceptualized the basic idea for the study, performed the statistical analysis together with MSI and RK. MRKC and SI prepared data for analysis. MRKC and MK prepared the first draft of the manuscript. HTAK and MR critically revised the manuscript for intellectual content. All authors have reviewed and approved the final manuscript.

For peer review only - http://bmjopen.bmj.com/site/about/guidelines.xhtml

Checklist for the appropriate reporting statement: Add STROBE checklist as supplementary.

A patient consent form: Not applicable

Data availability statement:

The datasets used and/or analysed during the current study are available from the corresponding author on reasonable request.

Supplementary and raw data: Supplementary Table 1 has been submitted with the main manuscript. The data underlying the results presented in the study are publicly accessible and available from the DHS website (https://dhsprogram.com/data/available-datasets.cfm). The name of the dataset is Bangladesh Demographic and Health Survey (BDHS).

For peer review only

For peer review only - http://bmjopen.bmj.com/site/about/guidelines.xhtml

Reference

1. Rahman MS, Howlader T, Masud MS, Rahman ML. Association of low-birth weight with malnutrition in children under five years in Bangladesh: do mother's education, socio-economic status, and birth interval matter? *PloS one.* 2016;11(6):e0157814.
2. Hasan MM, Uddin J, Pulok MH, Zaman N, Hajizadeh M. Socioeconomic Inequalities in Child Malnutrition in Bangladesh: Do They Differ by Region? *International journal of environmental research and public health.* 2020;17(3):1079.
3. McDonald CM, Olofin I, Flaxman S, et al. The effect of multiple anthropometric deficits on child mortality: meta-analysis of individual data in 10 prospective studies from developing countries. *The American journal of clinical nutrition.* 2013;97(4):896-901.
4. Fanzo J, Hawkes C, Udomkesmalee E, et al. 2018 Global Nutrition Report: Shining a light to spur action on nutrition. 2018.
5. Chowdhury MRK, Rahman MS, Khan MMH, Mondal MNI, Rahman MM, Billah B. Risk factors for child malnutrition in Bangladesh: a multilevel analysis of a nationwide population-based survey. *The Journal of pediatrics.* 2016;172:194-201. e191.
6. De P, Chattopadhyay N. Effects of malnutrition on child development: Evidence from a backward district of India. *Clinical Epidemiology and Global Health.* 2019;7(3):439-445.
7. NIPORT. *Demographic and Health Survey 2017-2018. National Institute of Population Research and Training.* 2019.
8. Islam MR, Rahman MS, Rahman MM, et al. Reducing childhood malnutrition in Bangladesh: the importance of addressing socio-economic inequalities. *Public health nutrition.* 2020;23(1):72-82.
9. Islam MS, Biswas T. Prevalence and correlates of the composite index of anthropometric failure among children under 5 years old in Bangladesh. *Maternal & child nutrition.* 2020;16(2):e12930.
10. Chowdhury MRK, Khan HT, Mondal MNI, Kabir R. Socio-demographic risk factors for severe malnutrition in children aged under five among various birth cohorts in Bangladesh. *Journal of Biosocial Science.* 2020:1-16.
11. WHO. *WHO child growth standards: length/height-for-age, weight-for-age, weight-for-length, weight-for-height and body mass index-for-age: methods and development.* World Health Organization; 2006.
12. Kismul H, Acharya P, Mapatano MA, Hatløy A. Determinants of childhood stunting in the Democratic Republic of Congo: further analysis of Demographic and Health Survey 2013–14. *BMC public health.* 2018;18(1):74.
13. Abera L, Dejene T, Laelago T. Magnitude of stunting and its determinants in children aged 6–59 months among rural residents of Damot Gale district; southern Ethiopia. *BMC research notes.* 2018;11(1):557.
14. Adhikari RP, Shrestha ML, Acharya A, Upadhaya N. Determinants of stunting among children aged 0–59 months in Nepal: findings from Nepal Demographic and health Survey, 2006, 2011, and 2016. *BMC nutrition.* 2019;5(1):37.
15. Khanam M, Shimul SN, Sarker AR. Individual-, household-, and community-level determinants of childhood undernutrition in Bangladesh. *Health services research and managerial epidemiology.* 2019;6:2333392819876555.
16. Khan S, Zaheer S, Safdar NF. Determinants of stunting, underweight and wasting among children< 5 years of age: evidence from 2012-2013 Pakistan demographic and health survey. *BMC Public Health.* 2019;19(1):358.

For peer review only - http://bmjopen.bmj.com/site/about/guidelines.xhtml

17. Croft TN, Marshall A, Allen C. Guide to DHS statistics. Rockville, Maryland, USA: ICF; 2018. In:2018.
18. Sreeramareddy CT, Shidhaye RR, Sathiakumar N. Association between biomass fuel use and maternal report of child size at birth-an analysis of 2005-06 India Demographic Health Survey data. *BMC public health.* 2011;11(1):1-10.
19. Khanal V, Sauer K, Karkee R, Zhao Y. Factors associated with small size at birth in Nepal: further analysis of Nepal Demographic and Health Survey 2011. *BMC pregnancy and childbirth.* 2014;14(1):1-9.
20. Haque SR, Tisha S, Huq N. Poor birth size a badge of low birth weight accompanying less antenatal care in Bangladesh with substantial divisional variation: evidence from BDHS-2011. *Birth.* 2015;1(2476):33.38.
21. Boregowda G, Soni G, Jain K, Agrawal S. Assessment of under nutrition using composite index of anthropometric failure (CIAF) amongst toddlers residing in Urban slums of Raipur City, Chhattisgarh, India. *Journal of clinical and diagnostic research: JCDR.* 2015;9(7):LC04.
22. Endris N, Asefa H, Dube L. Prevalence of malnutrition and associated factors among children in rural Ethiopia. *BioMed research international.* 2017;2017.
23. Al-Sadeeq AH, Bukair AZ, Al-Saqladi A-WM. Assessment of undernutrition using Composite Index of Anthropometric Failure among children aged< 5 years in rural Yemen. *Eastern Mediterranean Health Journal.* 2018;24(12).
24. Ziba M, Kalimbira AA, Kalumikiza Z. Estimated burden of aggregate anthropometric failure among Malawian children. *South African Journal of Clinical Nutrition.* 2018;31(2):20-23.
25. Bejarano IF, Oyhenart EE, Torres M, et al. Extended composite index of anthropometric failure in Argentinean preschool and school children. *Public health nutrition.* 2019;22(18):3327-3335.
26. Saha KK, Billah M, Menon P, El Arifeen S, Mbuya NV. *Bangladesh National Nutrition Services: assessment of implementation status.* The World Bank; 2015.
27. Tiwari R, Ausman LM, Agho KE. Determinants of stunting and severe stunting among under-fives: evidence from the 2011 Nepal Demographic and Health Survey. *BMC pediatrics.* 2014;14(1):1-15.
28. Varela-Silva MI, Azcorra H, Dickinson F, Bogin B, Frisancho A. Influence of maternal stature, pregnancy age, and infant birth weight on growth during childhood in Yucatan, Mexico: a test of the intergenerational effects hypothesis. *American Journal of Human Biology: The Official Journal of the Human Biology Association.* 2009;21(5):657-663.
29. Ntenda PAM. Association of low birth weight with undernutrition in preschool-aged children in Malawi. *Nutrition journal.* 2019;18(1):1-15.
30. Jahanihashemi H, Noroozi M, Zavoshy R, Afkhamrezaei A, Jalilolghadr S, Esmailzadehha N. Malnutrition and birth related determinants among children in Qazvin, Iran. *European journal of public health.* 2017;27(3):559-562.
31. Doyle LW. Growth and respiratory health in adolescence of the extremely low-birth weight survivor. *Clinics in perinatology.* 2000;27(2):421-432.
32. Ntenda PAM, Chuang Y-C. Analysis of individual-level and community-level effects on childhood undernutrition in Malawi. *Pediatrics & Neonatology.* 2018;59(4):380-389.
33. Bhowmik B, Siddique T, Majumder A, et al. Maternal BMI and nutritional status in early pregnancy and its impact on neonatal outcomes at birth in Bangladesh. *BMC pregnancy and childbirth.* 2019;19(1):1-14.

For peer review only - http://bmjopen.bmj.com/site/about/guidelines.xhtml

34. Lenters L, Wazny K, Bhutta ZA. Management of severe and moderate acute malnutrition in children. 2016.
35. Hossain MB, Khan MHR. Role of parental education in reduction of prevalence of childhood undernutrition in Bangladesh. *Public health nutrition.* 2018;21(10):1845-1854.
36. Nisbett N, Davis P, Yosef S, Akhtar N. Bangladesh's story of change in nutrition: Strong improvements in basic and underlying determinants with an unfinished agenda for direct community level support. *Global food security.* 2017;13:21-29.
37. Sinha RK, Dua R, Bijalwan V, Rohatgi S, Kumar P. Determinants of stunting, wasting, and underweight in five high-burden pockets of four Indian states. *Indian Journal of Community Medicine: Official Publication of Indian Association of Preventive & Social Medicine.* 2018;43(4):279.
38. Aiga H, Abe K, Andrianome VN, et al. Risk factors for malnutrition among school-aged children: a cross-sectional study in rural Madagascar. *BMC public health.* 2019;19(1):1-13.
39. Alderman H, Headey DD. How important is parental education for child nutrition? *World Development.* 2017;94:448-464.
40. Pieters J, Rawlings S. Parental unemployment and child health in China. *Review of Economics of the Household.* 2020;18(1):207-237.

For peer review only

For peer review only - http://bmjopen.bmj.com/site/about/guidelines.xhtml

Tables

Table 1 Background characteristics of the under-5 children

Factors	Number	Percentage (%)
**Total**	**7,661**	**100.0**
**Mothers' age (in years)**		
15-19	938	12.2
20-24	2,679	35.0
25-29	2,146	28.0
30-34	1,293	16.9
35-39	481	6.3
≥ 40	124	1.6
**Parents' education**		
Both parents with no formal education	294	3.8
Only father with no formal education	865	11.3
Only mother with no formal education	243	3.2
Both parents with no formal education	6,259	81.7
**Mother currently working**		
No	4,560	59.5
Yes	3,101	40.5
**Underweight mother**		
No	6,535	85.3
Yes (<18.5 kg/m^2)	1,126	14.7
**Mothers received antenatal care (n=4,540)**		
No	363	8.0
Yes	4,177	92.0
**Mothers received postnatal care (n=4,535)**		
No	1,509	33.3
Yes	3,026	66.7
**Mothers experience IPV** [a]		
No	6,264	81.8
Yes	1,397	18.2
**Mothers' decision-making autonomy** [b]		
Not practiced	1,083	14.1
Practiced	6,578	85.9
**Father's occupation**		
Currently not working	73	1.0
Manual labourer	5,458	71.2
Professional	472	6.2
Businessman	1,658	21.6
**Source of water**		
Improved	6,658	86.9
Unimproved	1,003	13.1
**Type of toilet facility**		
Improved	4,359	56.9
Unimproved	3,302	43.1
**Solid waste use in cooking**		
No	2,218	28.9
Yes	5,443	71.1
**Mass media exposure** [c]		
No	4,890	63.8
Yes	2,771	36.2
**Wealth index** [d]		
Poorest	1,708	22.2
Poorer	1,545	20.2
Middle	1,381	18.0
Richer	1,533	20.0
Richest	1,494	19.5

For peer review only - http://bmjopen.bmj.com/site/about/guidelines.xhtml

**Place of residence**		
Urban	2,605	34.0
Rural	5,056	66.0
**Children's age (in months)**		
0-11 months	1,673	21.8
12-23 months	1,583	20.7
24-35 months	1,475	19.3
36-47 months	1,417	18.5
48-59 months	1,513	19.7
**Sex of child**		
Male	3,995	52.2
Female	3,666	47.8
**Birth order**		
One	2,902	37.9
Two	2,507	32.7
Three	1,297	16.9
Four and above	955	12.5
**Low birth weight [e](n=4,735)**		
No	1,815	38.3
Yes (<2.5 kg)	326	6.9
Not weighted	2,594	54.8
**Currently had disease [f]**		
No	4,043	52.8
Yes	3,618	47.2

IPV, Intimate partner violence.
[a] a wife being beaten if she went out without telling her partner/ neglected the children/argued with her partner/burnt food/ was forced to have sex regardless of her consent
[b] a woman usually can decide by herself or jointly on her healthcare/large household purchases/visits to family or relatives
[c] exposed television/radio/newspaper/magazine to some extent
[d] integrating household asset ownership and access to drinking water and sanitation
[e] child's weight at birth measured as ≤2.5 kg
[f] child had at least one morbid condition out of diarrhea, fever, and cough in the two weeks preceding the survey

For peer review only

Table 2: Prevalence of cooccurrence and single dimension of undernutrition among under-5 children

Factors	Cooccurrence of undernutrition		Single dimension of undernutrition	
	Number (%)	Prevalence (95% CI)	Number (%)	Prevalence (95% CI)
**Total**	1528	19.3 (18.2-20.5)	1456	18.9 (17.9-19.9)
**Mothers' age (in years)**				
15-19	183	18.9 (16.2-21.9)	193	20.1 (17.3-23.2)
20-24	492	18.3 (16.6-20.1)	505	18.8 (17.2-20.6)
25-29	449	20.2 (18.4-22.1)	387	18.0 (16.2-19.9)
30-34	264	19.4 (16.8-22.2)	262	20.1 (17.9-22.6)
35-39	103	19.9 (16.2-24.2)	90	19.0 (15.3-23.3)
≥ 40	37	29.0 (20.8-38.9)	19	14.3 (8.8-22.3)
		p=0.160		*p*=0.533
**Parents' education**				
Both parents with no formal education	108	37.4 (31.0-44.3)	61	19.1 (14.7-24.5)
Only father with no formal education	221	25.6 (22.3-29.1)	199	23.0 (20.2-26.1)
Only mother with no formal education	75	28.0 (21.9-35.0)	52	21.8 (16.2-28.7)
Both parents with no formal education	1124	17.3 (16.2-18.5)	1144	18.2 (17.1-19.4)
		p<0.001		*p*=0.017
**Mother currently working**				
No	845	17.8 (16.4-19.3)	856	18.5 (17.2-20.0)
Yes	683	21.6 (19.9-23.4)	600	19.5 (17.9-21.1)
		p=0.0004		*p*=0.396
**Underweight mother**				
No	1182	17.7 (16.5-19.0)	1212	18.5 (17.4-19.7)
Yes (<18.5 kg/m^2)	346	29.5 (26.7-32.5)	244	21.2 (18.8-23.9)
		p<0.001		*p*=0.053
**Mothers received antenatal care**				
No	97	26.2 (21.6-31.3)	84	22.5 (18.2-27.5)
Yes	701	16.0 (14.8-17.4)	843	20.2 (18.8-21.5)
		p<0.001		*p*=0.327
**Mothers received postnatal care**				
No	219	14.2 (12.3-16.3)	299	19.5 (17.2-21.9)
Yes	578	18.3 (16.7-19.9)	628	20.8 (19.3-22.4)
		p=0.001		*p*=0.335
**Mothers experience IPV** [a]				
No	1209	18.7 (17.5-19.9)	1193	18.8 (17.7-20.0)
Yes	319	22.2 (19.8-24.8)	263	19.2 (17.1-21.5)
		p=0.005		*p*=0.744
**Mothers' decision-making autonomy** [b]				
Not practiced	231	20.5 (17.9-23.3)	202	17.8 (15.5-20.4)
Practiced	1297	19.2 (17.9-20.5)	1254	19.1 (18.0-20.2)
		p=0.370		*p*=0.377
**Father's occupation**				
Currently not working	21	28.1 (17.8-41.4)	10	13.0 (6.7-23.8)
Manual labourer	1181	20.9 (19.6-22.3)	1111	20.5 (19.4-21.7)
Professional	47	9.6 (7.1-12.9)	58	11.0 (8.4-14.4)
Businessman	279	16.0 (14.0-18.2)	277	15.7 (13.7-17.8)
		p<0.001		*p*<0.001
**Source of water**				
Improved	1349	19.7 (18.4-21.0)	1259	18.9 (17.8-20.0)
Unimproved	179	17.1 (14.6-19.9)	197	19.1 (16.7-21.8)
		p=0.092		*p*=0.857
**Type of toilet facility**				
Improved	772	17.4 (16.0-18.9)	776	17.7 (16.4-19.1)
Unimproved	756	21.9 (20.1-23.8)	680	20.5 (19.0-22.0)
		p=0.0001		*p*=0.006

For peer review only - http://bmjopen.bmj.com/site/about/guidelines.xhtml

**Solid waste use in cooking**				
No	330	15.3 (13.5-17.3)	372	16.8 (15.1-18.7)
Yes	1198	21.1 (19.7-22.5)	1084	19.8 (18.6-21.0)
		p<0.001		*p*=0.008
**Mass media exposure** [c]				
No	676	23.8 (21.9-25.8)	596	21.2 (19.6-23.0)
Yes	852	16.9 (15.7-18.3)	860	17.6 (16.4,19.0)
		p<0.001		*p*=0.0009
**Wealth index** [d]				
Poorest	460	26.4 (24.0-29.0)	384	22.1 (20.1-24.3)
Poorer	373	22.8 (20.5-25.2)	332	21.0 (18.8-23.3)
Middle	276	18.7 (16.3-21.4)	245	17.7 (15.6-19.9)
Richer	267	17.2 (14.9-19.8)	287	18.8 (16.7-21.1)
Richest	152	10.2 (8.5-12.3)	208	14.2 (12.1-16.5)
		p<0.001		*p*<0.001
**Place of residence**				
Urban	453	17.2 (15.2-19.5)	432	16.0 (14.3-17.8)
Rural	1075	20.1 (18.7-21.5)	1024	19.9 (18.7-21.2)
		p=0.032		*p*= 0.0004
**Children's age (in months)**				
0-11 months	219	12.0 (10.4-13.8)	267	16.2 (14.4-18.1)
12-23 months	290	17.3 (15.3-19.5)	368	22.9 (20.6-25.3)
24-35 months	334	22.4 (19.9-25.0)	336	22.9 (20.4-25.5)
36-47 months	320	22.3 (19.8-25.0)	280	19.3 (17.1-21.7)
48-59 months	365	23.9 (21.5-26.4)	205	13.3 (11.5-15.4)
		p<0.001		*p*<0.001
**Sex of child**				
Male	807	19.6 (18.0-21.2)	754	19.0 (17.6-20.4)
Female	721	19.1 (17.6-20.7)	702	18.8 (17.4-20.3)
		p=0.645		*p*=0.863
**Birth order**				
One	517	17.7 (16.1-19.5)	525	18.1 (16.6-19.7)
Two	464	17.9 (16.3-19.7)	467	18.6 (16.9-20.4)
Three	274	19.6 (17.4-22.0)	265	20.6 (18.2-23.2)
Four and above	273	27.6 (24.2-31.2)	199	19.9 (17.4-22.7)
		p<0.001		*p*=0.285
**Low birth weight** [e]				
No	197	10.5 (9.0-12.3)	313	17.6 (15.7-19.8)
Yes (<2.5 kg)	96	27.3 (22.3-32.9)	66	20.3 (15.7-25.8)
Not weighted	551	20.3 (18.6-22.2)	593	22.5 (20.9-24.2)
		p<0.001		*p*=0.002
**Currently had disease** [f]				
No	766	18.5 (17.1-20.1)	792	19.1 (17.7-20.5)
Yes	762	20.2 (18.6-21.9)	664	18.7 (17.3-20.2)
		p=0.103		*p*=0.699

CI, confidence interval; IPV, Intimate partner violence.
[a] a wife being beaten if she went out without telling her partner/ neglected the children/argued with her partner/burnt food/ was forced to have sex regardless of her consent
[b] a woman usually can decide by herself or jointly on her healthcare/large household purchases/visits to family or relatives
[c] exposed television/radio/newspaper/magazine to some extent
[d] integrating household asset ownership and access to drinking water and sanitation
[e] child's weight at birth measured as ≤2.5 kg
[f] child had at least one morbid condition out of diarrhea, fever, and cough in the two weeks preceding the survey

For peer review only - http://bmjopen.bmj.com/site/about/guidelines.xhtml

Table 3. Risk factors of cooccurrence and single dimension of under-5 child undernutrition

Factors	Cooccurrence		Single dimension	
	AOR (95% CI)	P values	AOR (95% CI)	P values
**Parents' education**				
Both parents with no formal education	1.64 (1.25-2.15)	<0.001	1.03 (0.76-1.39)	0.857
Only father with no formal education	1.17 (0.98-1.40)	0.080	1.19 (1.01-1.42)	0.050
Only mother with no formal education	1.51 (1.12-2.02)	0.006	1.11 (0.81-1.53)	0.517
Both parents with no formal education	1.00		1.00	
**Mothers currently working**				
No	1.00			
Yes	0.95 (0.84-1.07)	0.422		
**Underweight mothers**				
No	1.00			
Yes (<18.5 kg/m^2)	1.78 (1.53-2.06)	<0.001		
**Mothers received antenatal care**				
No	1.28 (0.98-1.67)	0.069		
Yes				
**Mothers received postnatal care**				
No	0.84 (0.70-1.02)	0.075		
Yes	1.00			
**Mother's experience IPV** [a]				
No	1.00			
Yes	1.11 (0.96-1.28)	0.162		
**Father's occupation**				
Currently not working	1.84 (1.07-3.16)	0.028	0.80 (0.41-1.59)	0.533
Manual labourer	1.12 (0.96-1.30)	0.149	1.17 (1.01-1.36)	0.042
Professional	0.81 (0.58-1.14)	0.230	0.80 (0.59-1.09)	0.157
Businessman	1.00		1.00	
**Type of toilet facility**				
Improved	1.00		1.00	
Unimproved	1.08 (0.94-1.23)	0.276	1.03 (0.90-1.18)	0.658
**Solid waste used in cooking**				
No	1.00		1.00	
Yes	1.03 (0.88-1.22)	0.695	0.99 (0.84-1.16)	0.869
**Mass media exposure** [b]				
No	1.05 (0.92-1.20)	0.493	1.10 (0.96-1.26)	0.180
Yes	1.00		1.00	
**Wealth index** [c]				
Poorest	2.29 (1.74-3.01)	<0.001	1.41 (1.09-1.82)	0.009
Poorer	2.28 (1.76-2.94)	<0.001	1.40 (1.10-1.78)	0.006
Middle	1.94 (1.52-2.48)	<0.001	1.17 (0.93-1.47)	0.192
Richer	1.70 (1.36-2.14)	<0.001	1.31 (1.06-1.61)	0.012
Richest	1.00		1.00	
**Place of residence**				
Urban	1.00		1.00	
Rural	0.92 (0.80-1.06)	0.232	1.09 (0.94-1.25)	0.246
**Children's age (in months)**				
0-11 months	1.00			
12-23 months	1.49 (1.23-1.81)	<0.001	1.61 (1.35-1.92)	<0.001
24-35 months	2.07 (1.71-2.50)	<0.001	1.57 (1.31-1.88)	<0.001
36-47 months	2.05 (1.69-2.49)	<0.001	1.31 (1.09-1.58)	0.004
48-59 months	2.18 (1.80-2.63)	<0.001	0.82 (0.67-1.00)	0.053
**Birth order**				
One	1.00			
Two	1.05 (0.91-1.21)	0.499		
Three	1.15 (0.97-1.36)	0.113		
Four and above	1.41 (1.17-1.70)	<0.001		
**Low birth weight** [d]				
No	1.00		1.00	
Yes	3.40 (2.52-4.57)	<0.001	1.18 (0.86-1.60)	0.257

AOR, adjusted odds ratio.
[a] a wife being beaten if she went out without telling her partner/ neglected the children/argued with her partner/burnt food/ was forced to have sex regardless of her consent
[b] exposed television/radio/newspaper/magazine to some extent

For peer review only

For peer review only - http://bmjopen.bmj.com/site/about/guidelines.xhtml

[c] integrating household asset ownership and access to drinking water and sanitation
[d] child's weight at birth measured as ≤2.5 kg

For peer review only

For peer review only - http://bmjopen.bmj.com/site/about/guidelines.xhtml

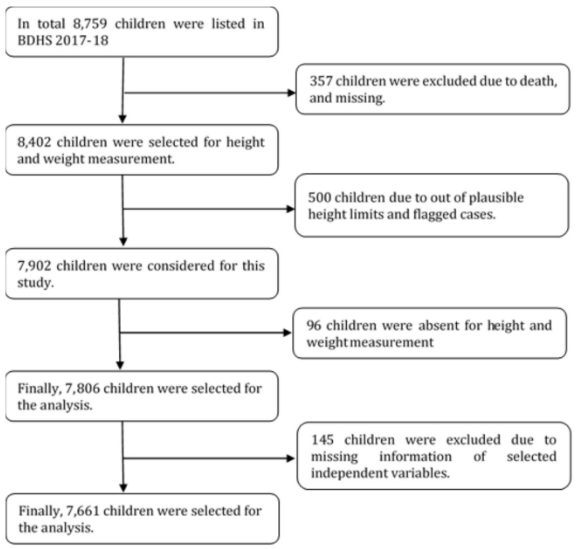

Figure 1: Sample size selection

Figure 1: Sample size selection

210x297mm (300 x 300 DPI)

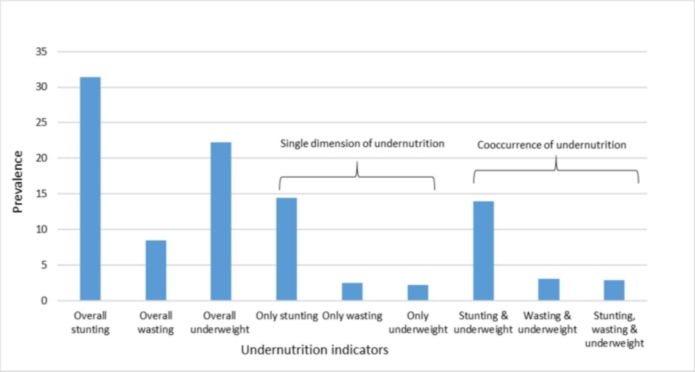

Figure 2: Prevalence of various forms of under-5 child undernutrition in Bangladesh

1

Figure 2: Prevalence of various forms of under-5 child undernutrition in Bangladesh

210x297mm (300 x 300 DPI)

Supplementary Table 1. Variable definitions

Variable	Type of variable	Description	Measurement	Scale of measurement	Number of responses/Mis sing
**Cooccurrence of malnutrition**	**Outcome variable**	coexistence of stunting and underweight; wasting and underweight at the same time or the coexistence of stunting, wasting and underweight (Figure 2)	No, yes	Binary	7,806/96
**Single dimension of malnutrition**	**Outcome variable**	a child with single dimension of undernutrition includes stunting, wasting or underweight only (Figure 2)	No, yes	Binary	7,806/96
**Mothers' age (in years)**	Independent variable	Age of mothers at the time of data collection	15-19, 20-24, 25-29, 30-34, 35-39, ≥ 40	Discrete, categorial	7,902/0
**Parents' education**	Independent variable	Parental educational status	Both parents were uneducated, only father was uneducated, only mother was uneducated, both parents were educated	Categorical	7,779/123
**Mother currently working**	Independent variable	Mothers engaged in economic activity at the time of data collection	No, yes	Binary	7,902/0
**Underweight mother**	Independent variable	Mother with <18.5 kg/m^2 of body mass index	No, yes	Binary	7,902/0
**Mothers received antenatal care**	Independent variable	Medical surveillance and review performed during pregnancy for the early detection of possible complications of pregnancy	No, yes	Binary	4,540/3,562
**Mothers received postnatal care**	Independent variable	Postnatal care is the individualised care provided to meet the needs of a mother and her baby following childbirth	No, yes	Binary	4,535/3,367
**Mothers' experience inmate partner violence**	Independent variable	In the BDHS surveys, IPV was measured using a shortened and modified version of the Conflict Tactics Scale and based on information such as physical violence	No, yes	Binary	7,902/0

For peer review only

		and sexual violence. Physical violence included a wife being beaten if she went out without telling her partner, if she neglected the children and if she ever argued with her partner. The incidence of sexual violence was calculated on a woman's positive response to a question asking whether she had ever been physically forced to have sexual intercourse even when she did not want to. For each of the questions, responses were re-coded dichotomously: 1= yes and 0 = no and IPV scores were calculated through adding up all four binary IPV indicators with maximum and minimum values of 4 and 0 respectively. These estimated scores were then recoded as a binary variable with categories labeled no IPV=0 and yes=1 (score 1- 4).			
**Mothers' decision-making autonomy**	Independent variable	in the BDHS surveys, a woman's decision-making power is assessed on the following three themes: 1) a woman who usually decides on her healthcare 2) a woman who usually decides on large household purchases and 3) a woman who usually decides on visits to family or relatives (NIPORT, 2013, 2016). The response options were as follows: (a) respondent alone, (b) respondent and husband/partner, (c) respondent and another person, (d) husband/partner alone, (e) someone else, (f) other. For each question, a value of 1 was assigned for inability in decision-making if the responses were d, e, or f and 0 for otherwise if the responses	Not practiced, Practiced	Binary	7,778/124

For peer review only

		were a, b, or c. The values were then added, resulting in a score from 0 to 3. The Cronbach's α for the instruments was 0·79, indicating high internal consistency.			
**Father's occupation**	Independent variable	Fathers engaged in economic activity at the time of data collection	Currently not working, manual labourer, professional, businessman	Categorical	7,772/130
**Source of water**	Independent variable	improved: piped into dwelling, piped to yard/plot, public tap/standpipe, piped to neighbor, tube well or borehole, protected well, protected spring, rainwater, tanker truck, cart with small tank, bottled water; unimproved: unprotected well, unprotected spring, surface water (river, dam, lake, pond, stream, canal, irrigation channel), other	Improved, unimproved	Binary	7,902/0
**Type of toilet facility**	Independent variable	improved: flush - to piped sewer system, flush - to septic tank, flush - to pit latrine, flush - don't know where, pit latrine - ventilated improved pit, pit latrine - with slab, composting toilet; unimproved: flush - to somewhere else, pit latrine - without slab / open pit, bucket toilet, hanging toilet/latrine, other	Improved, unimproved	Binary	7,902/0
**Solid waste use in cooking**	Independent variable	solid waste includes coal, lignite, charcoal, wood, straw / shrubs / grass, agricultural crop and animal dung; unsolid include electricity, natural gas, processed gas, biogas, kerosene	No, yes	Binary	7,896/8
**Mass media exposure**	Independent variable	mass media exposure through television, radio and newspaper/magazine has been defined as exposure to at least one	No, yes	Binary	7,902/0

For peer review only

		media that exposes to at least once a week			
**Wealth index**	Independent variable	wealth index in the DHS surveys is calculated, by the DHS authority, based on information on household characteristics and assets using principal component analysis. Then households are classified into quintiles based on the values of the wealth index, where households with lower values of the index is considered as poorest and vice-versa	Poorest, poorer, middle, richer, richest	Categorical	7,902/0
**Place of residence**	Independent variable		Urban, rural	Binary	7,902/0
**Children's age (in months)**	Independent variable	Age of the children at the time of data collection	0-11 months, 12-23 months, 24-35 months, 36-47 months, 48-59 months	Categorical	7,902/0
**Sex of child**	Independent variable	Sex differential of children	Male, female	Binary	7,902/0
**Birth order**	Independent variable	Birth order is the chronological order of sibling births in a family	One, two, three, four and above	Categorical	7,902/0
**Low birth weight**	Independent variable	Children were <2.5 kg of weight during birth	No, yes, not weighted	Categorical	4,735/3,167
**Currently had disease**	Independent variable	Children had at least cough, fever or diarrhea before 2 weeks of the survey	No, yes	Binary	7,899/3

For peer review only

STROBE 2007 (v4) Statement—Checklist of items that should be included in reports of *cross-sectional studies*

Manuscript: Differences in risk factors between cooccurrence and single dimension of undernutrition among under-5 children in Bangladesh

Section/Topic	Item #	Recommendation	Reported on page #
**Title and abstract**	1	(*a*) Indicate the study's design with a commonly used term in the title or the abstract	2
		(*b*) Provide in the abstract an informative and balanced summary of what was done and what was found	2-3
**Introduction**			
Background/rationale	2	Explain the scientific background and rationale for the investigation being reported	4-5
Objectives	3	State specific objectives, including any prespecified hypotheses	5
**Methods**			
Study design	4	Present key elements of study design early in the paper	5-6
Setting	5	Describe the setting, locations, and relevant dates, including periods of recruitment, exposure, follow-up, and data collection	6
Participants	6	(*a*) Give the eligibility criteria, and the sources and methods of selection of participants	6
Variables	7	Clearly define all outcomes, exposures, predictors, potential confounders, and effect modifiers. Give diagnostic criteria, if applicable	6-7
Data sources/ measurement	8*	For each variable of interest, give sources of data and details of methods of assessment (measurement). Describe comparability of assessment methods if there is more than one group	7 (online supplemental table 1)
Bias	9	Describe any efforts to address potential sources of bias	6, 8
Study size	10	Explain how the study size was arrived at	
Quantitative variables	11	Explain how quantitative variables were handled in the analyses. If applicable, describe which groupings were chosen and why	6-7
Statistical methods	12	(*a*) Describe all statistical methods, including those used to control for confounding	8
		(*b*) Describe any methods used to examine subgroups and interactions	8
		(*c*) Explain how missing data were addressed	5 (Figure 1)
		(*d*) If applicable, describe analytical methods taking account of sampling strategy	Not applicable
		(*e*) Describe any sensitivity analyses	8
**Results**			
Participants	13*	(a) Report numbers of individuals at each stage of study—eg numbers potentially eligible, examined for eligibility, confirmed eligible, included in the study, completing follow-up, and analysed	Page 5 (Figure 1)

For peer review only

		(b) Give reasons for non-participation at each stage	Page 5 (Figure 1)
		(c) Consider use of a flow diagram	Page 5 (Figure 1)
Descriptive data	14*	(a) Give characteristics of study participants (eg demographic, clinical, social) and information on exposures and potential confounders	8-9
		(b) Indicate number of participants with missing data for each variable of interest	8-9
Outcome data	15*	Report numbers of outcome events or summary measures	8-9
Main results	16	(*a*) Give unadjusted estimates and, if applicable, confounder-adjusted estimates and their precision (eg, 95% confidence interval). Make clear which confounders were adjusted for and why they were included	9
		(*b*) Report category boundaries when continuous variables were categorized	9
		(*c*) If relevant, consider translating estimates of relative risk into absolute risk for a meaningful time period	Not relevant
Other analyses	17	Report other analyses done—eg analyses of subgroups and interactions, and sensitivity analyses	9
**Discussion**			
Key results	18	Summarise key results with reference to study objectives	10-13
Limitations	19	Discuss limitations of the study, taking into account sources of potential bias or imprecision. Discuss both direction and magnitude of any potential bias	13
Interpretation	20	Give a cautious overall interpretation of results considering objectives, limitations, multiplicity of analyses, results from similar studies, and other relevant evidence	10-13
Generalisability	21	Discuss the generalisability (external validity) of the study results	13-14
**Other information**			
Funding	22	Give the source of funding and the role of the funders for the present study and, if applicable, for the original study on which the present article is based	15

*Give information separately for cases and controls in case-control studies and, if applicable, for exposed and unexposed groups in cohort and cross-sectional studies.

Note: An Explanation and Elaboration article discusses each checklist item and gives methodological background and published examples of transparent reporting. The STROBE checklist is best used in conjunction with this article (freely available on the Web sites of PLoS Medicine at http://www.plosmedicine.org/, Annals of Internal Medicine at http://www.annals.org/, and Epidemiology at http://www.epidem.com/). Information on the STROBE Initiative is available at www.strobe-statement.org.

For peer review only

BMJ Open

Differences in risk factors associated with the composite manner of single and multiple concurrent forms of undernutrition (stunting, wasting, or underweight) among children under 5 years in Bangladesh: A nationally representative cross-sectional study

Journal:	*BMJ Open*
Manuscript ID	bmjopen-2021-052814.R1
Article Type:	Original research
Date Submitted by the Author:	01-Sep-2021
Complete List of Authors:	Chowdhury, Mohammad ; First Capital University of Bangladesh, Department of Public Health<br>Khan, Hafiz; University of West London, College of Nursing, Midwifery and Healthcare<br>Rashid, Mamunur; University of Gävle, Department of Public Health and Sports Sciences<br>Kabir, Russell; Anglia Ruskin University<br>Islam, Sazin; First Capital University of Bangladesh, Department of Public Health<br>Shariful Islam, Md.; First Capital University of Bangladesh, Department of Public Health<br>Kader, Manzur; Karolinska Institutet, Institute of Environmental Medicine
**Primary Subject Heading**:	Public health
Secondary Subject Heading:	Paediatrics
Keywords:	Community child health < PAEDIATRICS, NUTRITION & DIETETICS, PUBLIC HEALTH, PREVENTIVE MEDICINE

SCHOLARONE™
Manuscripts

I, the Submitting Author has the right to grant and does grant on behalf of all authors of the Work (as defined in the below author licence), an exclusive licence and/or a non-exclusive licence for contributions from authors who are: i) UK Crown employees; ii) where BMJ has agreed a CC-BY licence shall apply, and/or iii) in accordance with the terms applicable for US Federal Government officers or employees acting as part of their official duties; on a worldwide, perpetual, irrevocable, royalty-free basis to BMJ Publishing Group Ltd ("BMJ") its licensees and where the relevant Journal is co-owned by BMJ to the co-owners of the Journal, to publish the Work in this journal and any other BMJ products and to exploit all rights, as set out in our licence.

The Submitting Author accepts and understands that any supply made under these terms is made by BMJ to the Submitting Author unless you are acting as an employee on behalf of your employer or a postgraduate student of an affiliated institution which is paying any applicable article publishing charge ("APC") for Open Access articles. Where the Submitting Author wishes to make the Work available on an Open Access basis (and intends to pay the relevant APC), the terms of reuse of such Open Access shall be governed by a Creative Commons licence – details of these licences and which Creative Commons licence will apply to this Work are set out in our licence referred to above.

Other than as permitted in any relevant BMJ Author's Self Archiving Policies, I confirm this Work has not been accepted for publication elsewhere, is not being considered for publication elsewhere and does not duplicate material already published. I confirm all authors consent to publication of this Work and authorise the granting of this licence.

For peer review only

Title: Differences in risk factors associated with the composite manner of single and multiple concurrent forms of undernutrition (stunting, wasting, or underweight) among children under 5 years in Bangladesh: A nationally representative cross-sectional study

Authors: Mohammad Rocky Khan Chowdhury[1], Hafiz TA Khan[2], Mamunur Rashid[3], Russell Kabir[4], Sazin Islam[1], Md. Shariful Islam[1], Manzur Kader*[5]

[1]Department of Public Health, First Capital University of Bangladesh, Chuadanga, Bangladesh

[2]College of Nursing, Midwifery and Healthcare University of West London, London, United Kingdom

[3]Department of Public Health and Sports Sciences, University of Gävle, Sweden

[4]School of Allied Health, Faculty of Health, Education, Medicine, and Social Care, Anglia Ruskin University, Chelmsford, Essex, United Kingdom

[1]Department of Public Health, First Capital University of Bangladesh, Chuadanga, Bangladesh

[1]Department of Public Health, First Capital University of Bangladesh, Chuadanga, Bangladesh

[5]Institute of Environmental Medicine, Karolinska Institutet, Stockholm, Sweden

Running title: Single and multiple concurrent forms of undernutrition among children in Bangladesh

*Correspondence to

Dr. Manzur Kader, PhD

Institute of Environmental Medicine, Karolinska Institute

Solnavägen 4, Torsplan floor 10, 113 65 Stockholm

Phone: +46 768 53 98 67 | manzur.kader@ki.se

For peer review only

Abstract

Objectives: The study aims at differentiating the risk factors of single and multiple concurrent forms of undernutrition among children under 5 years in Bangladesh.

Design: A nationally representative cross-sectional study.

Setting: Bangladesh.

Respondents: Children age under 5 years of age.

Outcome measure: This study considered two dichotomous outcome: single form (children without single form and children with single form) and multiple concurrent forms (children without multiple forms and children with multiple forms) of undernutrition.

Statistical analysis: Adjusted odds ratio (OR) and confidence interval (CI) of potential risk factors were calculated using logistic regression analysis.

Results: Around 38.2% of under-5 children are suffering undernutrition in Bangladesh. The prevalence of multiple concurrent forms and a single form of child undernutrition was 19.3% and 18.9%, respectively. The key risk factors of multiple concurrent forms of undernutrition were children born with low birth weight [OR: 3.37, 95% CI: 2.53, 4.49], lowest socio-economic quintile [OR: 2.35, 95% CI-1.89, 2.92] and children age group 48-59 months [OR: 2.15, 95% CI: 1.78, 2.60], in contrast, children age group 12-23 months [AOR: 1.60, 95% CI: 1.34, 1.91], socio-economically poorer households [OR: 1.57, 95% CI: 1.29, 1.92] and paternal illiteracy [OR: 1.19, 95% CI: 1.01, 1.43] was significantly associated with single form of undernutrition. Parental education, mother's nutritional status, children's age, birth order, and low birth weight were the differentiating risk factors of multiple concurrent forms and a single form of undernutrition.

Conclusion: One-fifth of the children are suffering multiple concurrent forms of undernutrition, which is similar as measured by the single form. Parental education, mother's undernutrition status, children's age, birth order, and low birth weight disproportionately affect multiple concurrent forms and a single form of undernutrition among under-5 children in Bangladesh, which should be considered to formulate an evidence-based strategy to reduce undernutrition among children.

Keywords: Children, malnutrition, prevalence, determinant, co-occurrence, and Bangladesh

For peer review only

Strengths and limitations of this study

To the best of our knowledge, this is the first study differentiating the factors associated with the composite manner of single and multiple concurrent forms of undernutrition among under-5 children in Bangladesh using a nationally representative, probability-based cross-sectional sample survey.

The multistage sampling technique of the national survey (BDHS 2017-18), including its sampling weight, helps reducing potential selection bias.

The study considered a wide range of variables to assess the risk factors of the single and multiple concurrent forms of undernutrition by adopting a robust analytical method.

All three indicators, such as stunting, wasting, and underweight, were used to formulate the study outcomes (e.g., composite manner single and multiple concurrent forms of undernutrition) without considering distinct associated factors that might affect the results.

The cross-sectional nature of the study sample does not allow establishing causation.

For peer review only

Introduction

Child undernutrition refers to deficiencies or imbalances in a child's intake of energy and/or nutrients, infectious disease, or a combination of both.[1] Worldwide, in 2020, approximately 149 million children under 5 were estimated to be stunted, 45 million were estimated to be wasted, and 85 million were underweight. About 45% of deaths in children are linked to these conditions.[1 2] Globally, 124 countries out of 141 (88%) have the burden of multiple concurrent forms of undernutrition among under-5 children. About 29% (41 countries) are struggling with high levels of all three concurrent forms of undernutrition (stunting, wasting, and underweight).[3] The multiple concurrent forms of undernutrition lead to a 12-fold elevated risk of child mortality than healthy children.[4] Also, globally, around 3% of children under five years were estimated to coexist stunting and wasting.[5] An effect of stunting, wasting, and underweight on mortality through reduced muscle mass suggests that young infants and children are especially vulnerable to undernutrition, particularly to multiple concurrent forms.[6] A reduced muscle or fat mass (a common mechanism involving limbs being both thinner and shorter) increases the risk of death considerably when wasting and stunting are present in the same child.[6] Fat stimulates the necessary energy to maintain the immune system.[6] a malnourished child with low muscle mass and reduced function of key organs, such as the heart, kidney, and immune system, is more likely to die during an acute food shortage.[6] Overall, undernutrition reduces the immunological capacity of a child to defend against diseases and recurrent infections, such as diarrhea, pneumonia, malaria, measles, and AIDS, which leads to an inconsistent growth and development of a child both mentally and physically.[7 8]

Child undernutrition is one of the global health problems, particularly in low-and middle-income countries, including Bangladesh. In Bangladesh, despite child undernutrition has declined over the years, rates of undernutrition are still among the highest in the world.[9] According to recent estimates, around 40% of children under-5 are suffering from undernutrition in Bangladesh.[10] The risk factors of child undernutrition are still an issue of deliberation in Bangladesh and other developing countries. Poor maternal education, low socioeconomic status, low birth weight, poor feeding practices, frequent infections, inadequate access to health care, and water and sanitation are key risk factors of child undernutrition in Bangladesh and other developing countries.[7 11-13] However, these risk factors of undernutrition were identified using conventional disaggregated indicators (e.g., stunting, wasting and underweight). The massages from those studies are not sufficient to formulate complete policy

For peer review only - http://bmjopen.bmj.com/site/about/guidelines.xhtml

decision-making as many children in Bangladesh (around 30%) and other developing countries are suffering from multiple concurrent forms of undernutrition that pose a threat to high mortality rate among children.[14]

Previously, factors associated with the single form (in a composite manner) and multiple concurrent forms (in composite manner) of undernutrition have not been identified in Bangladesh. However, Chowdhury et al. (2020) tried to link several socio-demographic factors with a composite manner of single and multiple forms of severe undernutrition.[14] This study appended multiple datasets of previous national surveys, which did not represent the current scenario and overall burden of single and multiple concurrent forms of undernutrition. Drivers or factors associated with single and concurrent forms of undernutrition are yet fully uncovered in Bangladesh and other developing countries. According to some recent evidence, age, sex, and food insecurity have been linked to multiple concurrent forms of undernutrition.[4 15 16]On the other hand, factors associated with the composite manner of a single form of undernutrition have not been widely investigated previously in developing countries, including Bangladesh. Studies on the composite manner of single and multiple concurrent forms of undernutrition might evaluate different results than finding grounded on conventional disaggregated indicators. Therefore, the present study aims at differentiating the risk factors of the composite manner of multiple concurrent forms and a single form of undernutrition among under-5 children in Bangladesh using a more recent nationally representative population-based survey. This study might be helpful for policymakers to address the nutritionally more vulnerable children.

Methodology

Study setting

Bangladesh is a densely populated country with 164.69 million populations; 14.33 are under 5 years of age.[17] Administratively, Bangladesh is divided into eight divisions: Barishal, Chattogram, Dhaka, Khulna, Mymensingh, Rajshahi, Rangpur, and Sylhet. Each division is divided into zilas (district) and each zila into upazilas (sub-district). Urban areas in an upazila are classified into wards, and wards are further subdivided into mohallas (suburb). Urban areas were further classified into two groups: city corporations and areas other than city corporations. Rural areas in an upazila are divided into union parishads (village), further subdivided into

For peer review only - http://bmjopen.bmj.com/site/about/guidelines.xhtml

mouzas (small villages). Therefore, the divisions separate the country as a whole into rural and urban areas. In Bangladesh, 54% of households are in rural areas.[10]

Management of undernutrition in Bangladesh

Undernutrition in Bangladesh is among the highest in the world. Bangladesh has developed facility-based (therapeutic care for malnourished children with complications) and community-based care (undernourished children without complications and children who have been discharged from facility-based inpatient care) for the management of malnourished children.[18 19] In line with this, Bangladesh's government has also introduced several policies and strategies to improve undernutrition. For example, in 1997, Bangladesh National Nutrition Council has developed a policy called the Bangladesh National Plan of Action for Nutrition (NPAN) to improve the nutritional status and quality of life of the people of Bangladesh.[20] National Nutritional policy Plan of action was approved on August 2008 by the Food Planning and Monitoring Committee to ensure an adequate and stable supply of safe and nutritious food, increase purchasing power and access to food of the people, and proper nutrition for all individuals, especially for women and children.[21] Again, the government of Bangladesh has taken the initiative to incorporate nutrition into public health and family planning services to improve the country's nutrition situation. Consequently, adopting National Nutritional Policy 2015 reflects the commitment of the State as a whole to improve the nutritional status and quality of life of the population especially disadvantaged groups, including mothers, adolescent girls, and children.[22] A new holistic policy called "Food and Nutrition Security Policy" has been adopted to promote the use of a "nutrition lens". This policy will help develop multi-sectoral inter-linked interventions that will improve the nutritional outcomes and cover the target period 2020-2030 in synchronization with the Sustainable Development Goal (SDG).[23]

Data source

After excluding missing information regarding selected variables, a total of 7,661 children under-5 were born in January 2013 or later were included in this study from Bangladesh Demographic Health Surveys (BDHS) 2017-18 (Figure 1). The BDHS 2017-18 data is nationally representative with a 99% response rate that includes data from Bangladeshi adults and children (both male and female) regarding demographic and social characteristics and health and nutritional indicators to monitor a wide range of the population. The data collection was started on 24 October 2017 and finished on 15 March 2018.[10] This survey was funded by

For peer review only - http://bmjopen.bmj.com/site/about/guidelines.xhtml

the United States Agency for International Development (USAID) and conducted by the National Institute of Population Research and Training (NIPORT) under the Ministry of Health and Family Welfare, Bangladesh. All survey-related issues were implemented by a Bangladeshi research organization 'Mitra and Associate' with technical support from the ICF International of Calverton, Maryland, USA. The 2017-18 BDHS was reviewed and approved by the ICF Macro Institutional Review Board (USA).[10]

These surveys were based on multistage stratified sampling techniques of households. At the first stage, 675 primary sampling units (PSUs) (250 PSU from urban and 425 PSU from rural) based on enumeration areas (clusters) from the census survey 2011 designed by the Bangladesh Bureau of Statistics were selected using probability proportional to size technique. Then, a systematic sample of 30 households from each PSU were selected at the final stage using an equal probability systematic sampling technique.[10] This multistage sampling technique, including its sampling weight, helps reducing potential sampling bias.[10] In BDHS data, sample weights were calculated in each sampling stage, considering each cluster and stratum that had been adjusted for non-response to obtain the final standard weights.[10] In addition, the survey interviewers interviewed all ever-married women aged 15-49 years from the pre-selected households without replacement and change in the implementing stage to prevent selection bias.[10] Informed consent was also obtained verbally from each participant to collect information about them and their children before enrolling in the study. A significant number of the study sample was illiterate, so verbal consent was considered the most suitable option to confirm participation.[10] Each BDHS used a standard questionnaire. Details of the survey questionnaire, sample design, data collection procedure, and other issues are discussed elsewhere.[10]

For peer review only

Outcome variables and operational definitions

The outcomes of the study were the co-occurrence and single dimension of undernutrition among under-5 children. A child was considered to be undernourished or stunted (short stature for age), wasted (dangerously thin), and underweight (low weight for age), respectively, if the height-for-age, weight-for-height, and weight-for-age indices were two standard deviations (SDs) or more below the respective median of the WHO reference population.[24] A child was considered to have multiple concurrent forms of undernutrition when stunting and underweight and wasting and underweight, or these three forms of undernutrition (stunting, wasting, and underweight) are present in the same child (Figure 2). Also, a child with a single form of

For peer review only - http://bmjopen.bmj.com/site/about/guidelines.xhtml

undernutrition (in a composite manner) includes a child is living with stunting, wasting, or underweight only (Figure 2). Detailed variables' scale of measurement with missing information has been clearly defined in online supplemental table 1. Children were categorized 0 for 'normal' and 1 for 'undernutrition' for each of the three indicators. After that, the values of all three indicators were added, which resulted in a score ranging from 0 to 3. The scores were again re-categorized as 0 for normal, 1 for a single form of undernutrition (in a composite manner), and 2 to 3 for multiple concurrent forms of undernutrition (in a composite manner). Detail classifications and coding of outcome variables used in this study have been clearly defined in online supplemental table 2.

Independent variables

Since 1990, the UNICEF conceptual framework on undernutrition has guided interventions from a multi-sectoral and multidimensional perspective.[25] This framework included the basic, underlying, and immediate causes of undernutrition. The basic causes address social, economic, environmental, and political issues that lead to the lack of or unequal distribution of financial, human, physical, social, and natural resources. The underlying causes focus on household food security, adequate care, and feeding practices, access to health services, and residing in a healthy environment. The basic and underlying causes lead to the immediate causes at the individual level through inadequate food intake and disease.[25]

The candidate variables (potential causes) considered for analysis in the present study were based on relevant previous literatures.[7 26-30] These variables include maternal age in years (15-19, 20-24, 25-29, 30-34, 35-39, ≥40); parents' education (both parents uneducated, only father was uneducated when mother was educated, only mother was uneducated when father was educated, both parents educated); mother's current working status (currently not working, currently working); mother's underweight status ((normal/average (≥18.5 kg/m^2), underweight (<18.5 kg/m^2)); status of mother's antenatal and postnatal care (not received, received); mother's experience of inmate partner violence (IPV) (not experienced, experienced: a wife being beaten by partner if she went out without telling him or/and neglected the children or/and she ever argued with her partner or/and burned food or/and refused to have sex);[10] mothers' decision-making autonomy (not experienced, experienced: a woman who usually decides by herself/jointly with husband at least on her healthcare or on large household purchases or visits to family or relatives);[10] father's occupation (currently not working, manual labourer, professional and businessman); source of drinking water (improved, unimproved),[31] use of

For peer review only - http://bmjopen.bmj.com/site/about/guidelines.xhtml

solid waste in cooking (solid, non-solid); type of toilet facility (improved, unimproved),[31] mass media exposure (no, yes: expose to either radio, television, newspapers, or magazines at least once a week), wealth index (integrating household asset ownership and access to drinking water and sanitation)[10] and place of residence (unban, rural). Moreover, various factors related to children, such as age, birth order, and birth weight status,[32-34] recent morbidity status (the child had at least one morbid condition out of diarrhea, fever, and cough in two weeks preceding the survey)[10] were included. Detailed variables' scale of measurement with missing information used in this study has been clearly defined in online supplemental table 3, 4 & 5 .

Patient and public involvement

Patients were not involved in the design, conduct, or interpretation of the study.

Statistical analysis

The background characteristics of the children and their parents were calculated using descriptive statistics. Crosstab analysis and Chi-square test (bivariate analysis) were used to determine the prevalence/percentages and associations (p values) between the selected independent variables and outcomes. Further, all independent variables found significant in bivariate analysis were simultaneously entered into the stepwise (forward selection) multivariate regression models for adjustment to identify risk factors for multiple concurrent forms and a single form of undernutrition. The findings from logistic regression analysis were assessed using odds ratio (OR) and confidence interval (CI). The significance level was set at $p < 0.25$ (2-tailed) in bivariate analyses instead of the traditional cutoff point 0.05 that can fail in identifying variables known to be important.[35] On the other hand, the significance level was set at $p < 0.05$ (2-tailed) in multivariate analyses. The data were analyzed by controlling cluster (PSU) and stratum (urban area: city corporation area + other than city corporation area and rural area) with sampling weights that represent the whole country, both urban and rural areas, to ensure the precision of the estimates. Multicollinearity was checked by examining the standard errors (SEs) of regression coefficients in the logistic regression analyses. An SE >2.0 indicates multicollinearity among the independent variables.[36] The SEs for the independent variables in the adjusted models for each outcome were <1, indicating an absence of multicollinearity. Stata version 14.2 (StataCorp LP, College Station, Texas) was used for all analyses considering the complex nature of the sampling weight of the BDHS. To adjust sampling weight in the analysis, the Stata command "svyset" was used.

For peer review only - http://bmjopen.bmj.com/site/about/guidelines.xhtml

Results

Table 1 shows that approximately 7% (both parents uneducated: 3.8% and only mothers were uneducated: 3.2%) mothers of children had no formal education, 15% of mothers were reported underweight, more than 90% of mothers of children received antenatal care, and one-third of them (33%) did not receive postnatal care and 18% of mothers experienced IPV. Around 42% of children were from poor households, and 66% were living in rural areas. The children's similar percentage distribution (approximately 20%) was observed among the various age categories, and 52% of them were male (Table 1).

Prevalence of multiple concurrent forms and a single form of undernutrition

In Bangladesh, the overall prevalence of child undernutrition was 38%. The overall prevalence of stunting, wasting, and underweight was 31%, 8%, and 22%, respectively (Figure 2). The prevalence of multiple concurrent forms (from Figure 1, stunting and underweight: 13.50%; wasting and underweight: 3.10%; and stunting, wasting, underweight: 2.74%) and single form (from Figure 1, only stunting: 14.32%, only wasting: 2.14% and only underweight: 2.44%) of undernutrition among under-5 children were approximately estimated at 19%. Further, the prevalence of multiple concurrent forms of undernutrition was significantly higher among children of uneducated parents (37%) when compared to educated parents (17%), children of underweight mothers (about 30%), children with fathers who were manual labourers (28%), children were ≥ 4 birth order (about 28%), and children born with low birth weight (27%) (Table 2).

The single form of undernutrition was highly prevalent among children of the uneducated father (when mothers are educated) (23%), children of age group 12-35 months (23%), those from the poorest section of the society (22%) when compared to the richest (14%), children of underweight mothers and households had no mass media exposure (21%), children of fathers who were manual labourer and households with unimproved toilet facility (21%) and children born with low birth weight (20%) (Table 3).

Risk factors

The key risk factors of multiple concurrent forms of undernutrition were children born with low birth weight (when compared to healthy weight children) [AOR: 3.37, 95% CI: 2.53, 4.49,

For peer review only - http://bmjopen.bmj.com/site/about/guidelines.xhtml

p<0.001]; children of lowest socioeconomic quintile (when compared to highest socioeconomic quintile) [AOR: 2.35, 95% CI: 1.89, 2.92, *p*<0.001]; children age group 48-59 months (when compared to children age group 0-11 months) [AOR: 2.15, 95% CI: 1.78, 2.60, *p*<0.001]; children with fathers who are currently unemployed (when compared to businessman fathers) [AOR: 1.83, 95% CI: 1.07, 3.16, *p*=0.028] and mothers being underweight (when compared to healthy mother) [AOR: 1.78, 95% CI: 1.54, 2.08, *p*<0.001] (Table 4).

In terms of a single form of undernutrition, children age group 12-23 months (when compared to children age group 0-11 months) [AOR: 1.60, 95% CI: 1.34, 1.91, *p*<0.001]; children of lowest socioeconomic quintile (when compared to highest socioeconomic quintile) [AOR: 1.57, 95% CI: 1.29, 1.92, *p*<0.001]; children of uneducated fathers when mothers are educated (when compared to educated parents) [AOR: 1.19, 95% CI: 1.00, 1.43, *p*=0.050] and children of fathers who are manual labourer (when compared to businessman fathers) [AOR: 1.17, 95% CI: 1.01, 1.36, *p*=0.038] were identified as key risk factors (Table 4).

Discussion

The prevalence of multiple concurrent forms and a single form of undernutrition among under-5 children in Bangladesh was nearly equal, accounting for 19%. One out of five children is suffering from both cases of undernutrition. This high figure is a concern when comparing it with some developing countries from Africa and North America, such as Ethiopia, Malawi and Argentina where the prevalence of co-occurrence of undernutrition among under-5 children was successively 26%, 12% and 2%. The single dimension of undernutrition were respectively 23%, 39%, and 5%.[37-39] Further, Asian countries such as Yemen and India are struggling with a high burden of both forms of undernutrition (co-occurrence of undernutrition: 48% and 39% respectively; single dimension of undernutrition: 21% and 24% respectively).[40 41] The findings indicated that Bangladesh has not still achieved sustainable improvement in reducing child undernutrition. One of the possible reasons could be that poor understanding of the multi-sectoral approach, lack of coordination among key government sectors, such as health, agriculture, education, urban and local development to address the issue of undernutrition, and poor linkage between key institutions, for example, government institutions, academic, research and training institutions, and national/international non-governmental organizations.[42]

This study reveals that children of uneducated mothers are living in less protective nature in Bangladesh. Three out of five children (65.4%) of uneducated mothers and two out of five

For peer review only - http://bmjopen.bmj.com/site/about/guidelines.xhtml

children (37.4%) experience multiple concurrent forms of undernutrition. Also, three out of ten children (27.3%) born with low birth weight experience multiple concurrent forms of undernutrition. Further, the odds of multiple concurrent forms of undernutrition were 3.37 times higher among children born with low birth weight than the children with healthy birth weight. Previous studies in Bangladesh, Pakistan, Nepal, Malawi, Mexico, and Iran also reported children born with low birth weight were more likely to explore malnutrition regardless of multiple concurrent forms and single form.[43-47] Children born with low birth weight generally increase their height and weight by small increments.[48] Thus, they may remain shorter and lighter and might be severely malnourished without adequate nutrition support. Children with low birth weights are often born to uneducated and underweight mothers.[49 50] Maternal/parental illiteracy is associated with poor maternal healthcare access, and caregiving to children often leads to an adverse nutritional outcome for mothers and children.[51] This study shows that the odds of multiple concurrent forms of undernutrition were 1.78 and 1.54 times higher among children of underweight mothers and mothers with no formal education, respectively. Numerous studies assessed these factors as key determinants of child undernutrition using disaggregated indicators and need more assessment to estimate the magnitude of risks in case of multiple concurrent forms and a single form of undernutrition.[7 11]

Other key risk factors of multiple concurrent forms of undernutrition include children of lowest socioeconomic quintile, children age group 48-59 months, children of fathers who are currently unemployed that differs by a single form of undernutrition to some extent additionally with children age group 12-23 months, children of uneducated fathers (when mothers are educated) and children of fathers who are labourers. An assessment of the risk difference between educated and uneducated parents and the richest and poorest socioeconomic status for multiple concurrent forms and a single form of undernutrition indicates higher educational and wealth inequalities for the co-occurrence of undernutrition. Further, the level of maternal education and economic hardship are not decidedly responsible for suffering from a single form of undernutrition among under-5 children. Earlier studies in Bangladesh and other South Asian countries highlighted parental education and wealth index as key risk factors of child undernutrition regardless of multiple concurrent forms and single form. Still, the studies need more precise estimation that could help policy makers introduce context-specific actions for reducing undernutrition.[7 11 28 30 52] Women who have low social status, and less education have very limited influence on household decision-making that might influence child nutritional

For peer review only - http://bmjopen.bmj.com/site/about/guidelines.xhtml

status. On the other hand, a large community in Bangladesh are concerned with broader issues of chronic poverty, and this would not reflect the improvement of undernutrition at a community level.[52] To reduce under-5 child undernutrition, increasing the participation of women and girls in education, especially in rural areas, minimizing the socioeconomic inequality by raising the income-generating sectors, and strengthening the nutrition-specific health programs are recommended.

Children of age group 48-59 months had a 215% higher risk of having multiple concurrent forms of undernutrition, while children aged 12-23 months were 60% more likely to have a single form of undernutrition. Many studies have consistently addressed age and older children to have a higher risk of being undernourished in Bangladesh, Nepal, Pakistan, Ethiopia, and Congo without classifying the two conditions.[26-30] However, in India, younger children had a higher likelihood of being undernourished.[53] Older children are less protective of adverse nutritional outcomes due to poor per capita food distribution among family members in large households and allocate less amount of household foods that inequitably meet age-specific energy requirements to young and older children.[54] The risk of poor parental education on multiple concurrent forms of undernutrition differs by paternal illiteracy (when mothers are educated) on a single dimension of undernutrition in identifying the impact of education on undernutrition. This study shows that paternal education was associated with a single form of undernutrition among under-5 children in Bangladesh. Evidence suggests that the impact of paternal education on undernutrition has not been widely addressed; alternatively, maternal education has more significant effects on child undernutrition than paternal education.[53] Parental education is associated with positive attitudes and behaviors that can reduce the incidence of child undernutrition to some extent. It helps to understand the complex nature of modern health care messages and behavioral changes are not answered by just mothers' most basic literacy and numeracy skills.[55] Reducing the gender gap for the enrolment in universal upper secondary education may help tackle the burden of all undernutrition.

This study also indicates that children of unemployed fathers were more likely to experience multiple concurrent forms of undernutrition; conversely, children of fathers who were labourers were less protective in the case of a single form of undernutrition. Additionally, three out of ten children experience multiple concurrent forms of undernutrition, and it was 21% for a single form. The effects of paternal unemployment on child undernutrition have not been widely explained, except in a study conducted in China. The study found a significant impact

For peer review only - http://bmjopen.bmj.com/site/about/guidelines.xhtml

of paternal unemployment on children undernutrition.[56] On the other hand, children of fathers having low paid jobs (labourer, farmer, etc.) have a greater risk of suffering undernutrition.[11 14] A loss of employment or low paid job is typically associated with lower income available for spending on market goods, including health care, non-household childcare, and nutritious consumption.[56] Increases in home-produced and time-intensive health investments could be recommended to tackle undernutrition.

This study strongly suggests some policy implications or interventions from developing countries' perspectives (e.g., Bangladesh) to successfully prevent and treat both forms of undernutrition, particularly the multiple concurrent forms based on evidence and broad-spectrum. Firstly, factors associated with undernutrition should be considered to formulate an evidence-based strategy to reduce undernutrition, particularly the multiple concurrent forms. Routine national and subnational level nutrition surveys such as Multiple Indicator Cluster Surveys (MICSs) need to be modified to include multiple concurrent forms of undernutrition to inform programme and policy decision-making.[57] Routine monitoring of the prevalence of multiple concurrent forms of undernutrition would be required to inform effective detection and treatment.[57] Community engagement and multiple concurrent forms of undernutrition screening could also be expanded in innovative methods by enrolling additional expertise and resources.[58] Innovative and early markers should be developed to predict, identify, and monitor children at short-term and long-term consequences due to multiple concurrent forms of undernutrition.[59] Maternal factors from adolescence through pregnancy need to be searched that adversely affect utero and postnatal child who is living with three forms of undernutrition.[59] Therapeutic interventions (e.g., ready-to-use therapeutic foods) must be reviewed and adjusted to ensure that the children at the highest mortality risk due to multiple concurrent forms of undernutrition are included. Comprehensive nutrition programmes must be developed to pursue SDG 2.2, to end undernutrition in all its forms by 2030.[59]

The study analyzed the data of the national survey. Thus, the findings can be applicable to the whole country and the low- and middle-income countries whose socio-demographic characteristics and healthcare settings are similar to Bangladesh. Another strength was to perform appropriate application of statistical tools that might help in understanding the findings. In addition, the multistage sampling technique, including its sampling weight, helps reducing potential selection bias. This study was not free from limitations. The cross-sectional nature of the data did not establish a causal relationship between risk factors and outcome

For peer review only - http://bmjopen.bmj.com/site/about/guidelines.xhtml

variables. All three indicators (e.g., stunting, wasting, and underweight) were used to formulate the study outcomes (composite manner single and multiple concurrent forms of undernutrition) without considering distinct associated factors that might affect the results. This study did not control other important indicators, such as the child's diet pattern, parents' behavioral factors, immunization, and ethnicity, due to lack of availability and missing values across the surveys. Another limitation may arise by involving recall bias or information bias, resulting from collecting information about self-reporting age, education, occupation, household assets, etc.

Conclusion

In Bangladesh, one out of five under-5 children is suffering multiple concurrent forms and a single form of undernutrition. Children born with low birth weight, the poorest socioeconomic status, and children age group 48-59 months were identified as key risk factors of co-occurrence of undernutrition. On the other hand, children age group 12-23 months, and paternal illiteracy had significant effects on single dimension of undernutrition. Parental education, underweight mothers, paternal employment status, age of children, birth order, and low birth weight are the main differentiating risk factors of multiple concurrent forms and a single form of undernutrition. The findings highlight factors that should be considered to formulate an evidence-based strategy to reduce undernutrition among under-5 children. In addition, concerted efforts are essential in making strong collaborations among multiple sectors, such as government, non-government, educational, social, cultural, and religious institutions, to improve nutritional status. The findings from this study may be recommended for low- and middle-income countries. However, further study is warranted in this regard.

For peer review only

Ethical statements

The BDHS was reviewed and approved by the ICF Macro Institutional Review Board (USA), which complies with all of the requirements of 45 CFR 46 "Protection of Human Subjects". The Bangladesh DHS was also reviewed and approved by the National Research Ethics Committee of the Bangladesh Medical Research Council (Dhaka, Bangladesh). Informed consent was also obtained verbally from each participant and their inmate partners (all ever-married women aged 15–49 years old) prior to being enrolled in the study. A significant number of the study sample was illiterate, so verbal consent was considered the most suitable option to confirm participation. The BDHSs also included samples of very young children (under five-year-old children either born in 2014 or later) in the data collection, and so mothers of these samples were asked to provide verbal consent on behalf of their children.

Acknowledgement: The authors thank the MEASURE DHS project for their support and free access to the original data and the Department of Public Health, First Capital University of Bangladesh, enabling the research scope.

Funding: This research received no specific grant from any institutions.

Competing interests: The authors declare that they have no competing interests.

Authors' contribution: MRKC conceptualized the basic idea for the study, performed the statistical analysis together with MSI and RK. MRKC and SI prepared data for analysis. MRKC and MK prepared the first draft of the manuscript. HTAK and MR critically revised the manuscript for intellectual content. All authors have reviewed and approved the final manuscript.

Checklist for the appropriate reporting statement: Add STROBE checklist as supplementary.

A patient consent form: Not applicable

Data sharing statement: Authors are responsible to provide the statistical code for software application on request.

Word count: 4,508 (excluding abstract, strength and limitation, reference, tables and figures)

For peer review only

Supplementary and raw data: Supplementary table 1, 2, 3, 4 & 5 have been submitted with the main manuscript. The data underlying the results presented in the study are publicly accessible and available from DHS website (https://dhsprogram.com/data/available-datasets.cfm). The name of the dataset is Bangladesh Demographic and Health Survey (BDHS) 2017-18.

For peer review only

For peer review only - http://bmjopen.bmj.com/site/about/guidelines.xhtml

Reference

1. WHO. Malnutrition. *World Health Organization (WHO) Switzerland: Geneva* 2021;Retrieved 22 August, 2021, from https://www.who.int/news-room/fact-sheets/detail/malnutrition

2. WHO. Underweight among children under 5 years of age (number in millions) (JME). The Global Health Observatory-Explore a world of health data. *World Health Organization (WHO) Switzerland: Geneva* 2021;Retrieved 22 August, 2021, from https://www.who.int/data/gho/data/indicators/indicator-details/GHO/gho-jme-underweight-numbers-(in-millions

3. McDonald CM, Olofin I, Flaxman S, et al. The effect of multiple anthropometric deficits on child mortality: meta-analysis of individual data in 10 prospective studies from developing countries. *The American journal of clinical nutrition* 2013;97(4):896-901.

4. Myatt M, Khara T, Schoenbuchner S, et al. Children who are both wasted and stunted are also underweight and have a high risk of death: a descriptive epidemiology of multiple anthropometric deficits using data from 51 countries. *Archives of Public Health* 2018;76(1):1-11.

5. Khara T, Mwangome M, Ngari M, et al. Children concurrently wasted and stunted: A meta-analysis of prevalence data of children 6–59 months from 84 countries. *Maternal & child nutrition* 2018;14(2):e12516.

6. Briend A, Khara T, Dolan C. Wasting and stunting—similarities and differences: policy and programmatic implications. *Food and nutrition bulletin* 2015;36(1_suppl1):S15-S23.

7. Chowdhury MRK, Rahman MS, Khan MMH, et al. Risk factors for child malnutrition in Bangladesh: a multilevel analysis of a nationwide population-based survey. *The Journal of pediatrics* 2016;172:194-201. e1.

8. De P, Chattopadhyay N. Effects of Malnutrition on child development: Evidence from a backward district of India. *Clinical Epidemiology and Global Health* 2019;7(3):439-45.

9. DI. 2020 Global Nutrition Report: Action on equity to end malnutrition. Bristol, UK:

Development Initiatives. *Development Initiatives* 2020

10. NIPORT. Demographic and Health Survey 2017–2018. *National Institute of Population Research and Training* 2019

For peer review only - http://bmjopen.bmj.com/site/about/guidelines.xhtml

11. Islam MR, Rahman MS, Rahman MM, et al. Reducing childhood malnutrition in Bangladesh: the importance of addressing socioeconomic inequalities. *Public health nutrition* 2020;23(1):72-82.
12. Islam MS, Biswas T. Prevalence and correlates of the composite index of anthropometric failure among children under 5 years old in Bangladesh. *Maternal & child nutrition* 2020;16(2):e12930.
13. Adhikari RP, Shrestha ML, Acharya A, et al. Determinants of stunting among children aged 0–59 months in Nepal: findings from Nepal Demographic and Health Survey, 2006, 2011, and 2016. *BMC nutrition* 2019;5(1):1-10.
14. Chowdhury MRK, Khan HT, Mondal MNI, et al. Socio-demographic risk factors for severe malnutrition in children aged under five among various birth cohorts in Bangladesh. *Journal of Biosocial Science* 2021;53(4):590-605.
15. Garenne M, Myatt M, Khara T, et al. Concurrent wasting and stunting among under-five children in Niakhar, Senegal. *Maternal & child nutrition* 2019;15(2):e12736.
16. Schoenbuchner SM, Dolan C, Mwangome M, et al. The relationship between wasting and stunting: a retrospective cohort analysis of longitudinal data in Gambian children from 1976 to 2016. *The American journal of clinical nutrition* 2019;110(2):498-507.
17. UN. United Nations (UN). Department of Economic and Social Affairs, Population Division (2019). World Population Prospects 2019. Retrieved 16 August, 2021, from https://population.un.org/wpp/. 2019
18. IPHN. National Guidelines for the Facility-based Management of Children with Severe Acute Malnutrition in Bangladesh. *Institute of Public Health Nutrition (IPHN) Director General of Health Services Ministry of Health and Family Welfare Government of People's Republic of Bangladesh* 2017;Retrieved 22 August, 2021, from https://scalingupnutrition.org/wp-content/uploads/2013/02/Bangladesh_NationalFoodPolicyPlanofAction_2008_2015.pdf
19. IPHN. National Guidelines for Community Based Management of Acute Malnutrition in Bangladesh. *Institute of Public Health Nutrition (IPHN) Director General of Health Services Ministry of Health and Family Welfare Government of People's Republic of Bangladesh* 2017;Retrieved 22 August, 2021, from https://www.humanitarianresponse.info/sites/www.humanitarianresponse.info/files/documents/files/cmam_guideline_revised_10_jul17.pdf

For peer review only

20. WHO. Bangladesh National Plan of Action for Nutrition (NPAN). Bangladesh National Nutrition Council *World Health Organization (WHO) Switzerland: Geneva Organization Global database on the implementation of nutrition action* 1997;Retrieved 22 August, 2021, from https://extranet.who.int/nutrition/gina/en/node/8230

21. FPMU. National Food Policy Plan of Action (2008-2015). *Food Planning and Monitoring Unit (FPMU) Ministry of Food and Disaster Management Dhaka, Bangladesh* 2008;Retrieved 22 August, 2021, from https://scalingupnutrition.org/wp-content/uploads/2013/02/Bangladesh_NationalFoodPolicyPlanofAction_2008_2015.pdf

22. NNP. National Nutrition Policy 2015. Nutrition Is the Foundation for Development. *National Nutrition Policy (NNP)* 2015;Retrieved August 22, 2021, from http://extwprlegs1.fao.org/docs/pdf/bgd152517.pdf

23. FAO. National Food and Nutrition Security Policy of Bangladesh (NFNSP). *Food and agriculture organization (FAO) in Bangladesh* 2019;Retrieved 22 August, 2021, from http://fpmu.gov.bd/agridrupal/sites/default/files/file/policy/NFNSP-2019-Draft-English.pdf

24. WHO. WHO child growth standards: length/height-for-age, weight-for-age, weight-for-length, weight-for-height and body mass index-for-age: methods and development: World Health Organization 2006.

25. Reinhardt K, Fanzo J. Addressing chronic malnutrition through multi-sectoral, sustainable approaches: a review of the causes and consequences. *Frontiers in nutrition* 2014;1:13.

26. Kismul H, Acharya P, Mapatano MA, et al. Determinants of childhood stunting in the Democratic Republic of Congo: further analysis of Demographic and Health Survey 2013–14. *BMC public health* 2018;18(1):74.

27. Abera L, Dejene T, Laelago T. Magnitude of stunting and its determinants in children aged 6–59 months among rural residents of Damot Gale district; southern Ethiopia. *BMC research notes* 2018;11(1):557.

28. Adhikari RP, Shrestha ML, Acharya A, et al. Determinants of stunting among children aged 0–59 months in Nepal: findings from Nepal Demographic and health Survey, 2006, 2011, and 2016. *BMC nutrition* 2019;5(1):37.

For peer review only - http://bmjopen.bmj.com/site/about/guidelines.xhtml

29. Khanam M, Shimul SN, Sarker AR. Individual-, household-, and community-level determinants of childhood undernutrition in Bangladesh. *Health services research and managerial epidemiology* 2019;6:2333392819876555.

30. Khan S, Zaheer S, Safdar NF. Determinants of stunting, underweight and wasting among children< 5 years of age: evidence from 2012-2013 Pakistan demographic and health survey. *BMC Public Health* 2019;19(1):358.

31. Croft T, Marshall A, Allen C. Guide to DHS statistics. Rockville, Maryland, USA: ICF; 2018, 2018.

32. Sreeramareddy CT, Shidhaye RR, Sathiakumar N. Association between biomass fuel use and maternal report of child size at birth-an analysis of 2005-06 India Demographic Health Survey data. *BMC public health* 2011;11(1):1-10.

33. Khanal V, Sauer K, Karkee R, et al. Factors associated with small size at birth in Nepal: further analysis of Nepal Demographic and Health Survey 2011. *BMC pregnancy and childbirth* 2014;14(1):1-9.

34. Haque SR, Tisha S, Huq N. Poor birth size a badge of low birth weight accompanying less antenatal care in Bangladesh with substantial divisional variation: evidence from BDHS-2011. *Birth* 2015;1(2476):33.8.

35. Bursac Z. Gauss CH Williams DK, & Hosmer DW (2008). *Purposeful selection of variables in logistic regression Source Code for Biology and Medicine*;3:17.

36. Chan Y. Biostatistics 202: logistic regression analysis. *Singapore medical journal* 2004;45(4):149-53.

37. Endris N, Asefa H, Dube L. Prevalence of Malnutrition and associated factors among children in rural Ethiopia. *BioMed research international* 2017;2017

38. Ziba M, Kalimbira AA, Kalumikiza Z. Estimated burden of aggregate anthropometric failure among Malawian children. *South African Journal of Clinical Nutrition* 2018;31(2):20-23.

39. Bejarano IF, Oyhenart EE, Torres MF, et al. Extended composite index of anthropometric failure in Argentinean preschool and school children. *Public health nutrition* 2019;22(18):3327-35.

40. Al-Sadeeq AH, Bukair AZ, Al-Saqladi A-WM. Assessment of undernutrition using Composite Index of Anthropometric Failure among children aged< 5 years in rural Yemen. *Eastern Mediterranean Health Journal* 2018;24(12)

41. Boregowda G, Soni G, Jain K, et al. assessment of under nutrition using composite index of anthropometric failure (CIAF) amongst toddlers residing in Urban slums of Raipur

For peer review only - http://bmjopen.bmj.com/site/about/guidelines.xhtml

City, Chhattisgarh, India. *Journal of clinical and diagnostic research: JCDR* 2015;9(7):LC04.

42. Saha KK, Billah M, Menon P, et al. Bangladesh National Nutrition Services: assessment of implementation status: World Bank Publications 2015.
43. Rahman MS, Howlader T, Masud MS, et al. Association of low-birth weight with malnutrition in children under five years in Bangladesh: do mother's education, socioeconomic status, and birth interval matter? *PloS one* 2016;11(6):e0157814.
44. Tiwari R, Ausman LM, Agho KE. Determinants of stunting and severe stunting among under-fives: evidence from the 2011 Nepal Demographic and Health Survey. *BMC pediatrics* 2014;14(1):1-15.
45. Varela-Silva MI, Azcorra H, Dickinson F, et al. Influence of maternal stature, pregnancy age, and infant birth weight on growth during childhood in Yucatan, Mexico: a test of the intergenerational effects hypothesis. *American Journal of Human Biology: The Official Journal of the Human Biology Association* 2009;21(5):657-63.
46. Ntenda PAM. Association of low birth weight with undernutrition in preschool-aged children in Malawi. *Nutrition journal* 2019;18(1):1-15.
47. Jahanihashemi H, Noroozi M, Zavoshy R, et al. malnutrition and birth related determinants among children in Qazvin, Iran. *European journal of public health* 2017;27(3):559-62.
48. Doyle LW. Growth and respiratory health in adolescence of the extremely low-birth weight survivor. *Clinics in perinatology* 2000;27(2):421-32.
49. Bhowmik B, Siddique T, Majumder A, et al. Maternal BMI and nutritional status in early pregnancy and its impact on neonatal outcomes at birth in Bangladesh. *BMC pregnancy and childbirth* 2019;19(1):1-14.
50. Ntenda PAM, Chuang Y-C. Analysis of individual-level and community-level effects on childhood undernutrition in Malawi. *Pediatrics & Neonatology* 2018;59(4):380-89.
51. Lenters L, Wazny K, Bhutta ZA. Management of severe and moderate acute Malnutrition in children. *Reproductive, maternal, newborn, and child health: disease control priorities 3rd edition Washington, DC: World Bank* 2016:205-23.
52. Nisbett N, Davis P, Yosef S, et al. Bangladesh's story of change in nutrition: Strong improvements in basic and underlying determinants with an unfinished agenda for direct community level support. *Global food security* 2017;13:21-29.
53. Sinha RK, Dua R, Bijalwan V, et al. Determinants of stunting, wasting, and underweight in five high-burden pockets of four Indian states. *Indian journal of community*

For peer review only - http://bmjopen.bmj.com/site/about/guidelines.xhtml

medicine: official publication of Indian Association of Preventive & Social Medicine 2018;43(4):279.

54. Aiga H, Abe K, Andrianome VN, et al. Risk factors for malnutrition among school-aged children: a cross-sectional study in rural Madagascar. *BMC Public Health* 2019;19(1):1-13.
55. Alderman H, Headey DD. How important is parental education for child nutrition? *World development* 2017;94:448-64.
56. Pieters J, Rawlings S. Parental unemployment and child health in China. *Review of Economics of the Household* 2020;18(1):207-37.
57. Odei Obeng-Amoako GA, Myatt M, Conkle J, et al. Concurrently wasted and stunted children 6-59 months in Karamoja, Uganda: prevalence and case detection. *Maternal & child nutrition* 2020;16(4):e13000.
58. Imam A, Hassan-Hanga F, Sallahdeen A, et al. A cross-sectional study of prevalence and risk factors for stunting among under-fives attending acute malnutrition treatment programmes in north-western Nigeria: Should these programmes be adapted to also manage stunting? *International Health* 2021;13(3):262-71.
59. Wells JC, Briend A, Boyd EM, et al. Beyond wasted and stunted—a major shift to fight child undernutrition. *The Lancet Child & Adolescent Health* 2019;3(11):831-34.

For peer review only

For peer review only - http://bmjopen.bmj.com/site/about/guidelines.xhtml

Tables

Table 1

Background characteristics of the under-5 children

Factors	Number	Percentage (%)
**Total**	**7,661**	**100.0**
**Mothers' age (in years)**		
15-19	938	12.2
20-24	2,679	35.0
25-29	2,146	28.0
30-34	1,293	16.9
35-39	481	6.3
≥ 40	124	1.6
**Parents' education**		
Both parents were uneducated	294	3.8
Only father was uneducated	865	11.3
Only mother was uneducated	243	3.2
Both parents were educated	6,259	81.7
**Mother currently working**		
No	4,560	59.5
Yes	3,101	40.5
**Underweight mother[a]**		
No	6,535	85.3
Yes(<18.5 kg/m^2)	1,126	14.7
**Mothers received antenatal care (n=4,540)**		
No	363	8.0
Yes	4,177	92.0
**Mothers received postnatal care (n=4,535)**		
No	1,509	33.3
Yes	3,026	66.7
**Mothers experience IPV[b]**		
No	6,264	81.8
Yes	1,397	18.2
**Mothers' decision-making autonomy[c]**		
Not practiced	1,083	14.1
Practiced	6,578	85.9
**Father's occupation**		
Currently not working	73	1.0
Manual labourer	5,458	71.2
Professional	472	6.2
Businessman	1,658	21.6
**Source of water**		
Improved	6,658	86.9
Unimproved	1,003	13.1
**Type of toilet facility**		
Improved	4,359	56.9
Unimproved	3,302	43.1
**Solid waste use in cooking**		
No	2,218	28.9
Yes	5,443	71.1
**Mass media exposure[d]**		
No	4,890	63.8
Yes	2,771	36.2
**Wealth index [e]**		
Poorest	1,708	22.2
Poorer	1,545	20.2
Middle	1,381	18.0
Richer	1,533	20.0

For peer review only

Richest	1,494	19.5
**Place of residence**		
Urban	2,605	34.0
Rural	5,056	66.0
**Children's age (in months)**		
0-11 months	1,673	21.8
12-23 months	1,583	20.7
24-35 months	1,475	19.3
36-47 months	1,417	18.5
48-59 months	1,513	19.7
**Sex of child**		
Male	3,995	52.2
Female	3,666	47.8
**Birth order**		
One	2,902	37.9
Two	2,507	32.7
Three	1,297	16.9
Four and above	955	12.5
**Low birthweight [f] (n=4,735)**		
No	1,815	38.3
Yes (<2.5 kg)	326	6.9
Not weighted	2,594	54.8
**Currently had disease [g]**		
No	4,043	52.8
Yes	3,618	47.2

[a] underweight measured as <18.5 kg/m^2

[b] a wife being beaten if she went out without telling her partner/ neglected the children/argued with her partner/burnt food/ was forced to have sex regardless of her consent

[c] a woman usually can decide by herself or jointly on her healthcare/large household purchases/visits to family or relatives

[d] exposed television/radio/newspaper/magazine to some extent

[e] integrating household asset ownership and access to drinking water and sanitation

[f] child's weight at birth measured as ≤2.5 kg

[g] child had at least one morbid condition out of diarrhea, fever and cough in the two weeks preceding the survey

For peer review only

For peer review only - http://bmjopen.bmj.com/site/about/guidelines.xhtml

Table 2

Prevalence of multiple concurrent forms of undernutrition among under-5 children

Factors	Multiple concurrent forms of undernutrition			
	No		Yes	
	Number (%)	Proportion (95% CI)	Number (%)	Proportion/prevalence (95% CI)
**Total**	6133	80.7 (79.5,81.8)	1528	19.3 (18.2-20.5)
**Mothers' age (in years)**				
15-19	755	81.1 (78.1-83.8)	183	18.9 (16.2-21.9)
20-24	2187	81.7 (79.9-83.4)	492	18.3 (16.6-20.1)
25-29	1697	79.8 (77.9-81.6)	449	20.2 (18.4-22.1)
30-34	1029	80.6 (77.8-83.2)	264	19.4 (16.8-22.2)
35-39	378	80.1 (75.8-83.8)	103	19.9 (16.2-24.2)
≥ 40	87	71.0 (61.1-79.2)	37	29.0 (20.8-38.9)
***p* values (Chi-square test)**			0.160	
**Parents' education**				
Both parents were uneducated	186	62.6 (55.7-69.0)	108	37.4 (31.0-44.3)
Only father was uneducated	644	74.4 (70.9-77.7)	221	25.6 (22.3-29.1)
Only mother was uneducated	168	72.0 (65.0-78.1)	75	28.0 (21.9-35.0)
Both parents were educated	5135	82.7 (81.5-83.8)	1124	17.3 (16.2-18.5)
***p* values (Chi-square test)**			<0.001	
**Mother currently working**				
No	3715	82.2 (80.7-83.6)	845	17.8 (16.4-19.3)
Yes	2418	78.4 (76.6-80.1)	683	21.6 (19.9-23.4)
***p* values (Chi-square test)**			0.0004	
**Underweight mother**				
No	5353	82.3 (81.0-83.5)	1182	17.7 (16.5-19.0)
Yes (<18.5 kg/m^2)	780	70.5 (67.5-73.3)	346	29.5 (26.7-32.5)
***p* values (Chi-square test)**			<0.001	
**Mothers received antenatal care**				
No	266	73.8 (68.7,78.4)	97	26.2 (21.6-31.3)
Yes	3476	84.0 (82.6,85.2)	701	16.0 (14.8-17.4)
***p* values (Chi-square test)**			<0.001	
**Mothers received postnatal care**				
No	1290	85.8 (83.7-87.7)	219	14.2 (12.3-16.3)
Yes	2448	81.7 (80.1-83.3)	578	18.3 (16.7-19.9)
			p=0.001	

For peer review only - http://bmjopen.bmj.com/site/about/guidelines.xhtml

**Mothers experienced IPV**				
No	5055	81.3 (80.1-82.5)	1209	18.7 (17.5-19.9)
Yes	1078	77.8 (75.2-80.2)	319	22.2 (19.8-24.8)
***p* values (Chi-square test)**		0.005		
**Mothers' decision-making autonomy**				
Not practiced	852	79.5 (76.7-82.1)	231	20.5 (17.9-23.3)
Practiced	5281	80.8 (79.5-82.1)	1297	19.2 (17.9-20.5)
***p* values (Chi-square test)**		0.370		
**Father's occupation**				
Currently not working	52	71.9 (58.6-82.2)	21	28.1 (17.8-41.4)
Manual labourer	4277	79.1 (77.7-80.4)	1181	20.9 (19.6-22.3)
Professional	425	90.4 (87.1-92.9)	47	9.6 (7.1-12.9)
Businessman	1379	84.0 (81.8-86.0)	279	16.0 (14.0-18.2)
***p* values (Chi-square test)**		*p*<0.001		
**Source of water**				
Improved	5309	80.3 (79.0-81.6)	1349	19.7 (18.4-21.0)
Unimproved	824	82.9 (80.1-85.4)	179	17.1 (14.6-19.9)
***p* values (Chi-square test)**		0.092		
**Type of toilet facility**				
Improved	3587	82.6 (81.1-84.0)	772	17.4 (16.0-18.9)
Unimproved	2546	78.1 (76.2-79.9)	756	21.9 (20.1-23.8)
		0.0001		
**Solid waste use in cooking**				
No	1888	84.7 (82.7-86.5)	330	15.3 (13.5-17.3)
Yes	4245	78.9 (77.5-80.3)	1198	21.1 (19.7-22.5)
***p* values (Chi-square test)**		<0.001		
**Mass media exposure**				
No	2095	76.2 (74.2-78.1)	676	23.8 (21.9-25.8)
Yes	4038	83.1 (81.7-84.3)	852	16.9 (15.7-18.3)
***p* values (Chi-square test)**		<0.001		
**Wealth index**				
Poorest	1248	73.6 (71.0-76.0)	460	26.4 (24.0-29.0)
Poorer	1172	77.2 (74.8-79.5)	373	22.8 (20.5-25.2)
Middle	1105	81.3 (78.6-83.7)	276	18.7 (16.3-21.4)
Richer	1266	82.8 (80.2-85.1)	267	17.2 (14.9-19.8)
Richest	1342	89.8 (87.7-91.5)	152	10.2 (8.5-12.3)
***p* values (Chi-square test)**		*p*<0.001		
**Place of residence**				

For peer review only

Urban	2152	82.8 (80.5-84.8)	453	17.2 (15.2-19.5)
Rural	3981	79.9 (78.5-81.3)	1075	20.1 (18.7-21.5)
***p* values (Chi-square test)**			0.032	
**Children's age (in months)**				
0-11 months	1454	88.0 (86.2-89.6)	219	12.0 (10.4-13.8)
12-23 months	1293	82.7 (80.5-84.7)	290	17.3 (15.3-19.5)
24-35 months	1141	77.6 (75.0-80.1)	334	22.4 (19.9-25.0)
36-47 months	1097	77.7 (75.0-80.2)	320	22.3 (19.8-25.0)
48-59 months	1148	76.1 (73.6-78.5)	365	23.9 (21.5-26.4)
***p* values (Chi-square test)**			<0.001	
**Sex of child**				
Male	3188	80.4 (78.8-82.0)	807	19.6 (18.0-21.2)
Female	2945	80.9 (79.3-82.4)	721	19.1 (17.6-20.7)
***p* values (Chi-square test)**			0.645	
**Birth order**				
One	2385	82.3 (80.5-83.9)	517	17.7 (16.1-19.5)
Two	2043	82.1 (80.3-83.7)	464	17.9 (16.3-19.7)
Three	1023	80.4 (78.0-82.6)	274	19.6 (17.4-22.0)
Four and above	682	72.4 (68.8-75.8)	273	27.6 (24.2-31.2)
***p* values (Chi-square test)**			<0.001	
**Low birth weight**				
No	1618	89.5 (87.7-91.0)	197	10.5 (9.0-12.3)
Yes (<2.5 kg)	230	72.7 (67.1-77.7)	96	27.3 (22.3-32.9)
Not weighted	2043	79.7 (77.8-81.4)	551	20.3 (18.6-22.2)
***p* values (Chi-square test)**			<0.001	
**Currently had disease**				
No	3277	81.5 (79.9-82.9)	766	18.5 (17.1-20.1)
Yes	2856	79.8 (78.1-81.4)	762	20.2 (18.6-21.9)
***p* values (Chi-square test)**			0.103	

No undenutrition for multiple concurrent forms includes healthy children and children with single form of undernutrition
CI, confidence interval

For peer review only - http://bmjopen.bmj.com/site/about/guidelines.xhtml

Table 3

Prevalence of single form of undernutrition among under-5 children

Factors	Single form of undernutrition			
	No		Yes	
	Number (%)	Proportion (95% CI)	Number (%)	Proportion/prevalence (95% CI)
**Total**	6205	81.1 (80.1-82.1)	1456	18.9 (17.9-19.9)
**Mothers' age (in years)**				
15-19	745	79.9 (76.8-82.7)	193	20.1 (17.3-23.2)
20-24	2174	81.2 (79.4-82.8)	505	18.8 (17.2-20.6)
25-29	1759	82.0 (80.1-83.8)	387	18.0 (16.2-19.9)
30-34	1031	79.9 (77.4-82.1)	262	20.1 (17.9-22.6)
35-39	391	81.0 (76.7-84.7)	90	19.0 (15.3-23.3)
≥ 40	105	85.7 (77.7-91.2)	19	14.3 (8.8-22.3)
***p* values (Chi-square test)**			0.533	
**Parents' education**				
Both parents were uneducated	233	80.9 (75.5-85.3)	61	19.1 (14.7-24.5)
Only father was uneducated	666	77.0 (73.9-79.8)	199	23.0 (20.2-26.1)
Only mother was uneducated	191	78.2 (71.3-83.8)	52	21.8 (16.2-28.7)
Both parents were educated	5115	81.8 (80.6-82.9)	1144	18.2 (17.1-19.4)
***p* values (Chi-square test)**			0.017	
**Mother currently working**				
No	3704	81.5 (80.0-82.8)	856	18.5 (17.2-20.0)
Yes	2501	80.5 (78.9-82.1)	600	19.5 (17.9-21.1)
***p* values (Chi-square test)**			0.396	
**Underweight mother**				
No	5323	81.5 (80.3-82.6)	1212	18.5 (17.4-19.7)
Yes (<18.5 kg/m^2)	882	78.8 (76.1-81.2)	244	21.2 (18.8-23.9)
***p* values (Chi-square test)**			0.053	
**Mothers received antenatal care**				
No	279	77.5 (72.5-81.8)	84	22.5 (18.2-27.5)
Yes	3334	79.8 (78.5-81.2)	843	20.2 (18.8-21.5)
***p* values (Chi-square test)**			0.327	
**Mothers received postnatal care**				
No	1210	80.5 (78.1-82.8)	299	19.5 (17.2-21.9)
Yes	2398	79.2 (77.6-80.7)	628	20.8 (19.3-22.4)
			0.335	
**Mothers experienced IPV**				
No	5071	81.2 (80.0-82.3)	1193	18.8 (17.7-20.0)
Yes	1134	80.8 (78.5-82.9)	263	19.2 (17.1-21.5)
***p* values (Chi-square test)**			0.744	
**Mothers' decision-making autonomy**				
Not practiced	881	82.2 (79.6-84.5)	202	17.8 (15.5-20.4)
Practiced	5324	80.9 (79.8-82.0)	1254	19.1 (18.0-20.2)
***p* values (Chi-square test)**			0.377	
**Father's occupation**				
Currently not working	63	87.0 (76.2-93.3)	10	13.0 (6.7-23.8)

For peer review only - http://bmjopen.bmj.com/site/about/guidelines.xhtml

For peer review only

Manual labourer	4347	79.5 (78.3-80.6)	1111	20.5 (19.4-21.7)
Professional	414	89.0 (85.6-91.6)	58	11.0 (8.4-14.4)
Businessman	1381	84.3 (82.2-86.3)	277	15.7 (13.7-17.8)
***p* values (Chi-square test)**		<0.001		
**Source of water**				
Improved	5399	81.1 (80.0-82.2)	1259	18.9 (17.8-20.0)
Unimproved	806	80.9 (78.2-83.3)	197	19.1 (16.7-21.8)
***p* values (Chi-square test)**		0.857		
**Type of toilet facility**				
Improved	3583	82.3 (80.9-83.6)	776	17.7 (16.4-19.1)
Unimproved	2622	79.5 (78.0-81.0)	680	20.5 (19.0-22.0)
		0.006		
**Solid waste use in cooking**				
No	1846	83.2 (81.3-84.9)	372	16.8 (15.1-18.7)
Yes	4359	80.2 (79.0-81.4)	1084	19.8 (18.6-21.0)
***p* values (Chi-square test)**		0.008		
**Mass media exposure**				
No	2175	78.8 (77.0-80.4)	596	21.2 (19.6-23.0)
Yes	4030	82.4 (81.0-83.6)	860	17.6 (16.4,19.0)
***p* values (Chi-square test)**		0.0009		
**Wealth index**				
Poorest	1324	77.9 (75.7-79.9)	384	22.1 (20.1-24.3)
Poorer	1213	79.0 (76.7-81.2)	332	21.0 (18.8-23.3)
Middle	1136	82.3 (80.1-84.4)	245	17.7 (15.6-19.9)
Richer	1246	81.2 (78.9-83.3)	287	18.8 (16.7-21.1)
Richest	1286	85.8 (83.5-87.9)	208	14.2 (12.1-16.5)
***p* values (Chi-square test)**		<0.001		
**Place of residence**				
Urban	2173	84.0 (82.2-85.7)	432	16.0 (14.3-17.8)
Rural	4032	80.1 (78.8-81.3)	1024	19.9 (18.7-21.2)
***p* values (Chi-square test)**		0.0004		
**Children's age (in months)**				
0-11 months	1406	83.8 (81.9-85.6)	267	16.2 (14.4-18.1)
12-23 months	1215	77.1 (74.7-79.4)	368	22.9 (20.6-25.3)
24-35 months	1139	77.1 (74.5-79.6)	336	22.9 (20.4-25.5)
36-47 months	1137	80.7 (78.3-82.9)	280	19.3 (17.1-21.7)
48-59 months	1308	86.7 (84.6-88.5)	205	13.3 (11.5-15.4)
***p* values (Chi-square test)**		0.001		
**Sex of child**				
Male	3241	81.0 (79.6-82.4)	754	19.0 (17.6-20.4)
Female	2964	81.2 (79.7-82.6)	702	18.8 (17.4-20.3)
***p* values (Chi-square test)**		=0.863		
**Birth order**				
One	2377	81.9 (80.3-83.4)	525	18.1 (16.6-19.7)
Two	2040	81.4 (79.6-83.1)	467	18.6 (16.9-20.4)
Three	1032	79.4 (76.8-81.8)	265	20.6 (18.2-23.2)
Four and above	756	80.1 (77.3-82.6)	199	19.9 (17.4-22.7)
***p* values (Chi-square test)**		0.285		
**Low birth weight**				
No	1502	82.4 (80.2-84.3)	313	17.6 (15.7-19.8)
Yes (<2.5 kg)	260	79.7 (74.2-84.3)	66	20.3 (15.7-25.8)
Not weighted	2001	77.5 (75.8-79.1)	593	22.5 (20.9-24.2)
***p* values (Chi-square test)**		0.002		
**Currently had disease**				
No	3251	80.9 (79.5-82.3)	792	19.1 (17.7-20.5)
Yes	2954	81.3 (79.8-82.7)	664	18.7 (17.3-20.2)
***p* values (Chi-square test)**		0.699		

No undenutrition for single form includes healthy children and children with multiple concurrent forms of undernutrition

CI, confidence interval

For peer review only

For peer review only

For peer review only - http://bmjopen.bmj.com/site/about/guidelines.xhtml

Table 4
Risk factors of multiple concurrent forms and single form of under-5 child undernutrition (results of stepwise logistic regression with forward selection)

Factors	Multiple concurrent forms		Single form	
	AOR (95% CI)	*p* values	AOR (95% CI)	*p* values
**Parents' education** [a,b]				
Both parents were uneducated	1.68 (1.29-2.19)	<0.001	1.04 (0.77-1.40)	0.796
Only father was uneducated	1.18 (0.99-1.41)	0.065	1.19 (1.00-1.43)	0.050
Only mother was uneducated	1.54 (1.15-2.06)	0.004	1.11 (0.81-1.53)	0.514
Both parents were educated	1.00		1.00	
**Underweight mothers** [a,b]				
No	1.00			
Yes	1.78 (1.54-2.07)	<0.001		
**Father's occupation** [a,b]				
Currently not working	1.83 (1.07-3.16)	0.028	0.82 (0.41-1.61)	0.557
Manual labourer	1.11 (0.96-1.30)	0.161	1.17 (1.01-1.36)	0.038
Professional	0.81 (0.57-1.13)	0.207	0.80 (0.59-1.09)	0.160
Businessman	1.00		1.00	
**Wealth index** [a,b]				
Poorest	2.35 (1.89-2.92)	<0.001	1.57 (1.29-1.92)	<0.001
Poorer	2.28 (1.83-2.83)	<0.001	1.52 (1.24-1.86)	<0.001
Middle	1.92 (1.54-2.40)	<0.001	1.23 (1.00-1.51)	0.054
Richer	1.70 (1.36-2.11)	<0.001	1.34 (1.10-1.64)	0.004
Richest	1.00		1.00	
**Children's age (in months)** [a,b]				
0-11 months	1.00		1.00	
12-23 months	1.48 (1.22-1.80)	<0.001	1.60 (1.34-1.91)	<0.001
24-35 months	2.05 (1.69-2.48)	<0.001	1.56 (1.30-1.87)	<0.001
36-47 months	2.03 (1.68-2.46)	<0.001	1.31 (1.09-1.58)	0.005
48-59 months	2.15 (1.78-2.60)	<0.001	0.82 (0.67-0.99)	0.043
**Birth order** [a,b]				
One	1.00			
Two	1.05 (0.91-1.21)	0.539		
Three	1.14 (0.96-1.35)	0.127		
Four and above	1.41 (1.17-1.69)	<0.001		
**Low birth weight** [b]				
No	1.00			
Yes	3.37 (2.53-4.49)	<0.001		

a, stepwise logistic regression model for multiple concurrent forms of undernutrition includes maternal age, parents' education, mother's current working status, mother's underweight status, mother's experience IPV, father's occupation, use of solid waste in cooking, type of toilet facility, mass media exposure, wealth index, place of residence, age of children and birth order

b, stepwise logistic regression model for multiple concurrent forms of undernutrition includes maternal age, parents' education, mother's current working status mother's underweight status, status of mother's antenatal and postnatal care, mother's experience IPV, father's occupation, use of solid waste in cooking, type of toilet facility, mass media exposure, wealth index, place of residence, age of children, birth order, and birth weight status

a, stepwise logistic regression model for single form of undernutrition includes parents' education, mother's underweight status, father's occupation, use of solid waste in cooking, type of toilet facility, mass media exposure, wealth index, place of residence and age of children

b, stepwise logistic regression model for multiple concurrent forms of undernutrition includes parents' education, mother's underweight status, father's occupation, use of solid waste in cooking, type of toilet facility, mass media exposure, wealth index, place of residence, age of children and birth weight status

AOR indicates adjusted odds ratio.

For peer review only - http://bmjopen.bmj.com/site/about/guidelines.xhtml

For peer review only

Figure 1

Sample size selection

Figure 2

Trends of the prevalence of stunting, wasting and underweight

For peer review only

For peer review only - http://bmjopen.bmj.com/site/about/guidelines.xhtml

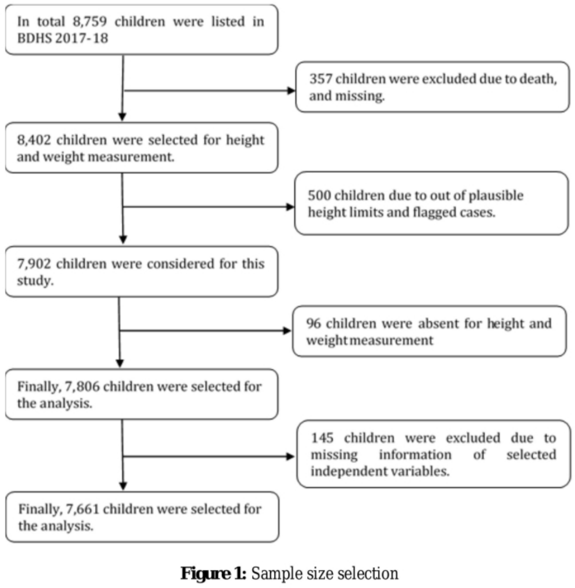

Figure 1: Sample size selection

210x297mm (300 x 300 DPI)

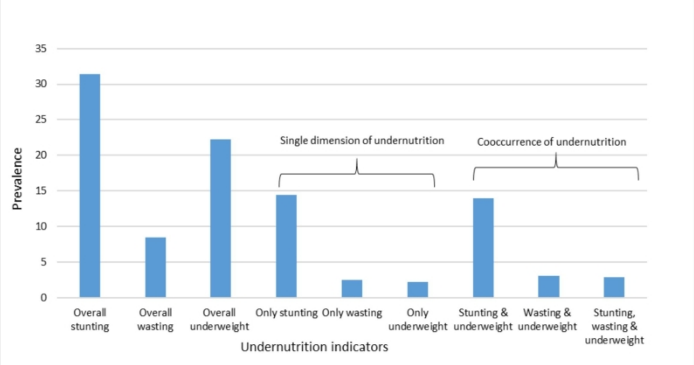

Figure 2: Prevalence of various forms of under-5 child undernutrition in Bangladesh

1

Figure 2: Prevalence of various forms of under-5 child undernutrition in Bangladesh

210x297mm (300 x 300 DPI)

Supplementary Table 1

Definitions of outcome variables

Variable	Type of variable	Description	Measurement	Scale of measurement	Number of responses/Mis sing
**Multiple concurrent forms of undernutrition**	**Outcome variable**	A child was considered to have multiple concurrent forms of undernutrition when stunting and underweight and wasting and underweight, or these three forms of undernutrition (stunting, wasting, and underweight) are present in the same child (Figure 2)	No, yes	Binary	7,806/96
**Single form of undernutrition**	**Outcome variable**	A child with a single form of undernutrition (in a composite manner) includes a child is living with stunting, wasting, or underweight only (Figure 2)	No, yes	Binary	7,806/96

For peer review only

Supplementary Table 2

Estimating scores for a child with different nutritional status

Nutritional indicators	Code	Stunting yes (1)/no (0)	wasting yes (1)/no (0)	underweight yes (1)/no (0)	Total score
**Stunning**					
No	0				
Yes	1				
**Wasting**					
No	0				
Yes	1				
**Underweight**					
No	0				
Yes	1				
**Healthy children/no undernutrition**		0	0	0	0
**Only stunting**		1	0	0	1
**Only wasting**		0	1	0	1
**Only underweight**		0	0	1	1
**Stunting & wasting**		1	0	1	2
**Stunting & underweight**		1	0	1	2
**Wasting and underweight**		0	1	1	2
**Stunting, wasting and underweight**		1	1	1	3

For peer review only

Supplementary Table 3

Variable definitions (parental characteristics)

Variable	Type of variable	Description	Measurement	Scale of measurement	Number of responses/Mis sing
**Mothers' age (in years)**	Independent variable	Age of mothers at the time of data collection	15-19, 20-24, 25-29, 30-34, 35-39, ≥ 40	Discrete, categorial	7,902/0
**Parents' education**	Independent variable	Parental educational status	Both parents were uneducated, only father was uneducated, only mother was uneducated, both parents were educated	Categorical	7,779/123
**Mother currently working**	Independent variable	Mothers engaged in economic activity at the time of data collection	No, yes	Binary	7,902/0
**Underweight mother**	Independent variable	Mother with <18.5 kg/m^2 of body mass index	No, yes	Binary	7,902/0
**Mothers received antenatal care**	Independent variable	Medical surveillance and review performed during pregnancy for the early detection of possible complications of pregnancy	No, yes	Binary	4,540/3,562
**Mothers received postnatal care**	Independent variable	Postnatal care is the individualised care provided to meet the needs of a mother and her baby following childbirth	No, yes	Binary	4,535/3,367
**Mothers' experience inmate partner violence**	Independent variable	In the BDHS surveys, IPV was measured using a shortened and modified version of the Conflict Tactics Scale and based on information such as physical violence and sexual violence. Physical violence included a wife being beaten if she went out without telling her partner, if she neglected the children and if she ever argued with her partner. The incidence of sexual violence was calculated on a woman's positive response to a question asking whether she had ever been physically forced to	No, yes	Binary	7,902/0

For peer review only

		have sexual intercourse even when she did not want to. For each of the questions, responses were re-coded dichotomously: 1= yes and 0 = no and IPV scores were calculated through adding up all four binary IPV indicators with maximum and minimum values of 4 and 0 respectively. These estimated scores were then recoded as a binary variable with categories labeled no IPV=0 and yes=1 (score 1- 4).			
**Mothers' decision-making autonomy**	Independent variable	in the BDHS surveys, a woman's decision-making power is assessed on the following three themes: 1) a woman who usually decides on her healthcare 2) a woman who usually decides on large household purchases and 3) a woman who usually decides on visits to family or relatives. The response options were as follows: (a) respondent alone, (b) respondent and husband/partner, (c) respondent and another person, (d) husband/partner alone, (e) someone else, (f) other. For each question, a value of 1 was assigned for inability in decision-making if the responses were d, e, or f and 0 for otherwise if the responses were a, b, or c. The values were then added, resulting in a score from 0 to 3. The Cronbach's α for the instruments was 0·79, indicating high internal consistency.	Not practiced, Practiced	Binary	7,778/124
**Father's occupation**	Independent variable	Fathers engaged in economic activity at the time of data collection	Currently not working, manual labourer, professional, businessman	Categorical	7,772/130

For peer review only

Supplementary Table 4

Variable definitions (household and contextual factors)

Variable	Type of variable	Description	Measurement	Scale of measurement	Number of responses/Mis sing
**Source of water**	Independent variable	improved: piped into dwelling, piped to yard/plot, public tap/standpipe, piped to neighbor, tube well or borehole, protected well, protected spring, rainwater, tanker truck, cart with small tank, bottled water; unimproved: unprotected well, unprotected spring, surface water (river, dam, lake, pond, stream, canal, irrigation channel), other	Improved, unimproved	Binary	7,902/0
**Type of toilet facility**	Independent variable	improved: flush - to piped sewer system, flush - to septic tank, flush - to pit latrine, flush - don't know where, pit latrine - ventilated improved pit, pit latrine - with slab, composting toilet; unimproved: flush - to somewhere else, pit latrine - without slab / open pit, bucket toilet, hanging toilet/latrine, other	Improved, unimproved	Binary	7,902/0
**Solid waste use in cooking**	Independent variable	solid waste includes coal, lignite, charcoal, wood, straw / shrubs / grass, agricultural crop and animal dung; unsolid include electricity, natural gas, processed gas, biogas, kerosene	No, yes	Binary	7,896/8
**Mass media exposure**	Independent variable	mass media exposure through television, radio and newspaper/magazine has been defined as exposure to at least one media that exposes to at least once a week	No, yes	Binary	7,902/0
**Wealth index**	Independent variable	wealth index in the DHS surveys is calculated, by the DHS authority, based on information on household characteristics and assets using	Poorest, poorer, middle, richer, richest	Categorical	7,902/0

For peer review only

		principal component analysis. Then households are classified into quintiles based on the values of the wealth index, where households with lower values of the index is considered as poorest and vice-versa			
**Place of residence**	Independent variable		Urban, rural	Binary	7,902/0

For peer review only

Supplementary Table 5

Variable definitions (child characteristics)

Variable	Type of variable	Description	Measurement	Scale of measurement	Number of responses/Mis sing
**Children's age (in months)**	Independent variable	Age of the children at the time of data collection	0-11 months, 12-23 months, 24-35 months, 36-47 months, 48-59 months	Categorical	7,902/0
**Sex of child**	Independent variable	Sex differential of children	Male, female	Binary	7,902/0
**Birth order**	Independent variable	Birth order is the chronological order of sibling births in a family	One, two, three, four and above	Categorical	7,902/0
**Low birth weight**	Independent variable	Children were <2.5 kg of weight during birth. Approximately 75% mothers can correctly report their baby's size at birth; therefore mother's recall is a valid proxy measure of birth weight.	No, yes, not weighted	Categorical	4,735/3,167
**Currently had disease**	Independent variable	Children had at least cough, fever or diarrhea before 2 weeks of the survey	No, yes	Binary	7,899/3

For peer review only

STROBE 2007 (v4) Statement—Checklist of items that should be included in reports of *cross-sectional studies*

Section/Topic	Item #	Recommendation	Reported on page #
**Title and abstract**	1	(*a*) Indicate the study's design with a commonly used term in the title or the abstract	2
		(*b*) Provide in the abstract an informative and balanced summary of what was done and what was found	2
**Introduction**			
Background/rationale	2	Explain the scientific background and rationale for the investigation being reported	4-6
Objectives	3	State specific objectives, including any prespecified hypotheses	5-6
**Methods**			
Study design	4	Present key elements of study design early in the paper	6-8
Setting	5	Describe the setting, locations, and relevant dates, including periods of recruitment, exposure, follow-up, and data collection	6-8
Participants	6	(*a*) Give the eligibility criteria, and the sources and methods of selection of participants	7-8
Variables	7	Clearly define all outcomes, exposures, predictors, potential confounders, and effect modifiers. Give diagnostic criteria, if applicable	9-11
Data sources/ measurement	8*	For each variable of interest, give sources of data and details of methods of assessment (measurement). Describe comparability of assessment methods if there is more than one group	11 (online supplemental table 2)
Bias	9	Describe any efforts to address potential sources of bias	8
Study size	10	Explain how the study size was arrived at	Figure 1
Quantitative variables	11	Explain how quantitative variables were handled in the analyses. If applicable, describe which groupings were chosen and why	online supplemental table 2
Statistical methods	12	(*a*) Describe all statistical methods, including those used to control for confounding	11-12
		(*b*) Describe any methods used to examine subgroups and interactions	11
		(*c*) Explain how missing data were addressed	7 (Figure 1)
		(*d*) If applicable, describe analytical methods taking account of sampling strategy	11-12
		(*e*) Describe any sensitivity analyses	11
**Results**			
Participants	13*	(a) Report numbers of individuals at each stage of study—eg numbers potentially eligible, examined for eligibility, confirmed eligible, included in the study, completing follow-up, and analysed	Page 7 (Figure 1)
		(b) Give reasons for non-participation at each stage	Page 7 (Figure 1)
		(c) Consider use of a flow diagram	Page 7 (Figure 1)

For peer review only

Descriptive data	14*	(a) Give characteristics of study participants (eg demographic, clinical, social) and information on exposures and potential confounders	12-13
		(b) Indicate number of participants with missing data for each variable of interest	online supplemental table 2
Outcome data	15*	Report numbers of outcome events or summary measures	12
Main results	16	(*a*) Give unadjusted estimates and, if applicable, confounder-adjusted estimates and their precision (eg, 95% confidence interval). Make clear which confounders were adjusted for and why they were included	12-13
		(*b*) Report category boundaries when continuous variables were categorized	online supplemental table 2
		(*c*) If relevant, consider translating estimates of relative risk into absolute risk for a meaningful time period	Not relevant
Other analyses	17	Report other analyses done—eg analyses of subgroups and interactions, and sensitivity analyses	13
**Discussion**			
Key results	18	Summarise key results with reference to study objectives	14-19
Limitations	19	Discuss limitations of the study, taking into account sources of potential bias or imprecision. Discuss both direction and magnitude of any potential bias	18-19
Interpretation	20	Give a cautious overall interpretation of results considering objectives, limitations, multiplicity of analyses, results from similar studies, and other relevant evidence	14-17
Generalisability	21	Discuss the generalisability (external validity) of the study results	19
**Other information**			
Funding	22	Give the source of funding and the role of the funders for the present study and, if applicable, for the original study on which the present article is based	20

*Give information separately for cases and controls in case-control studies and, if applicable, for exposed and unexposed groups in cohort and cross-sectional studies.

Note: An Explanation and Elaboration article discusses each checklist item and gives methodological background and published examples of transparent reporting. The STROBE checklist is best used in conjunction with this article (freely available on the Web sites of PLoS Medicine at http://www.plosmedicine.org/, Annals of Internal Medicine at http://www.annals.org/, and Epidemiology at http://www.epidem.com/). Information on the STROBE Initiative is available at www.strobe-statement.org.

For peer review only

BMJ Open

Differences in risk factors associated with single and multiple concurrent forms of undernutrition (stunting, wasting, or underweight) among children under 5 in Bangladesh: A nationally representative cross-sectional study

Journal:	*BMJ Open*
Manuscript ID	bmjopen-2021-052814.R2
Article Type:	Original research
Date Submitted by the Author:	16-Oct-2021
Complete List of Authors:	Chowdhury, Mohammad ; First Capital University of Bangladesh, Department of Public Health<br>Khan, Hafiz; University of West London, College of Nursing, Midwifery and Healthcare<br>Rashid, Mamunur; University of Gävle, Department of Public Health and Sports Sciences<br>Kabir, Russell; Anglia Ruskin University<br>Islam, Sazin; First Capital University of Bangladesh, Department of Public Health<br>Shariful Islam, Md.; First Capital University of Bangladesh, Department of Public Health<br>Kader, Manzur; Karolinska Institutet, Institute of Environmental Medicine
**Primary Subject Heading**:	Public health
Secondary Subject Heading:	Paediatrics
Keywords:	Community child health < PAEDIATRICS, NUTRITION & DIETETICS, PUBLIC HEALTH, PREVENTIVE MEDICINE

SCHOLARONE™
Manuscripts

I, the Submitting Author has the right to grant and does grant on behalf of all authors of the Work (as defined in the below author licence), an exclusive licence and/or a non-exclusive licence for contributions from authors who are: i) UK Crown employees; ii) where BMJ has agreed a CC-BY licence shall apply, and/or iii) in accordance with the terms applicable for US Federal Government officers or employees acting as part of their official duties; on a worldwide, perpetual, irrevocable, royalty-free basis to BMJ Publishing Group Ltd ("BMJ") its licensees and where the relevant Journal is co-owned by BMJ to the co-owners of the Journal, to publish the Work in this journal and any other BMJ products and to exploit all rights, as set out in our licence.

The Submitting Author accepts and understands that any supply made under these terms is made by BMJ to the Submitting Author unless you are acting as an employee on behalf of your employer or a postgraduate student of an affiliated institution which is paying any applicable article publishing charge ("APC") for Open Access articles. Where the Submitting Author wishes to make the Work available on an Open Access basis (and intends to pay the relevant APC), the terms of reuse of such Open Access shall be governed by a Creative Commons licence – details of these licences and which Creative Commons licence will apply to this Work are set out in our licence referred to above.

Other than as permitted in any relevant BMJ Author's Self Archiving Policies, I confirm this Work has not been accepted for publication elsewhere, is not being considered for publication elsewhere and does not duplicate material already published. I confirm all authors consent to publication of this Work and authorise the granting of this licence.

For peer review only

Title: Differences in risk factors associated with single and multiple concurrent forms of undernutrition (stunting, wasting, or underweight) among children under 5 in Bangladesh: A nationally representative cross-sectional study

Authors: Mohammad Rocky Khan Chowdhury[1,2], Hafiz TA Khan[2], Mamunur Rashid[3], Russell Kabir[4], Sazin Islam[1], Md. Shariful Islam[1], Manzur Kader*[5]

[1]Department of Public Health, First Capital University of Bangladesh, Chuadanga, Bangladesh

[2]College of Nursing, Midwifery and Healthcare, University of West London, London, United Kingdom

[3]Department of Public Health and Sports Sciences, University of Gävle, Sweden

[4]School of Allied Health, Faculty of Health, Education, Medicine, and Social Care, Anglia Ruskin University, Chelmsford, Essex, United Kingdom

[5]Institute of Environmental Medicine, Karolinska Institutet, Stockholm, Sweden

Running title: Single and multiple concurrent forms of undernutrition among children in Bangladesh

*Correspondence to

Dr. Manzur Kader, PhD

Institute of Environmental Medicine, Karolinska Institute

Solnavägen 4, Torsplan floor 10, 113 65 Stockholm

Phone: +46 768 53 98 67 | manzur.kader@ki.se

For peer review only

Abstract

Objectives: The study aims to differentiate the risk factors of single and multiple concurrent forms of undernutrition among children under 5 in Bangladesh.

Design: A nationally representative cross-sectional study.

Setting: Bangladesh.

Respondents: Children age under 5 years of age.

Outcome measure: This study considered two dichotomous outcomes: single form (children without single form and with single form) and multiple concurrent forms (children without multiple forms and with multiple forms) of undernutrition.

Statistical analysis: Adjusted odds ratio (OR) and confidence interval (CI) of potential risk factors were calculated using logistic regression analysis.

Results: Around 38.2% of children under 5 in Bangladesh are suffering from undernutrition. The prevalence of multiple concurrent forms and single form of child undernutrition was 19.3% and 18.9%, respectively. The key risk factors of multiple concurrent forms of undernutrition were children born with low birth weight [AOR: 3.76, 95% CI: 2.78, 5.10]; children in the age group 24-35 months [AOR: 2.70, 95% CI: 2.20, 3.30], and in the lowest socioeconomic quintile [AOR: 2.57, 95% CI: 2.05, 3.23]. In contrast, those children in the age group 24-35 months [AOR: 1.94, 95% CI: 1.61, 2.34], in the lowest socioeconomic quintile [AOR: 1.79, 95% CI: 1.45, 2.21] and born with low birth weight [AOR: 1.52, 95% CI: 1.11, 2.08] were significantly associated with a single form of undernutrition. Parental education, father's occupation, children's age, and birth order were the differentiating risk factors for multiple concurrent forms and single form of undernutrition.

Conclusion: One-fifth of children under 5 years of age are suffering multiple concurrent forms of undernutrition, which is similar to the numbers suffering the single form. Parental education, father's occupation, children's age and birth order disproportionately affect the multiple concurrent forms and single form of undernutrition, which should be considered to formulate an evidence-based strategy for reducing undernutrition among these children.

Keywords: Children, malnutrition, prevalence, determinant, co-occurrence, and Bangladesh

For peer review only

Strengths and limitations of this study

To the best of our knowledge, this is the first study to differentiate the factors associated with single and multiple concurrent forms of undernutrition among children aged under 5 in Bangladesh and is based on a nationally representative, probability-based cross-sectional sample survey.

The multistage sampling technique of the national survey (BDHS 2017-18) used in this study, including its sampling weight, helped in reducing potential selection bias.

The study considered a wide range of variables to assess the risk factors of the single and multiple concurrent forms of undernutrition by adopting a robust analytical method.

All three indicators, such as stunting, wasting, and underweight, were used to formulate the study outcomes without considering distinct associated factors that might affect the results.

The cross-sectional nature of the study sample does not allow for establishing causation.

For peer review only

Introduction

Child undernutrition refers to deficiencies or imbalances in a child's intake of energy and/or nutrients, infectious disease, or a combination of both.[1] In 2020, approximately 149 million children worldwide aged under 5 were estimated to be stunted, with 45 million estimated to be wasted, and 85 million underweight. About 45% of deaths in children are linked to these conditions.[1 2] The burden of multiple concurrent forms of undernutrition among under 5 year old children is present in 124 countries out of 141 (88%) with 41 countries (around 29%) struggling with high levels of all three concurrent forms of undernutrition (stunting, wasting, and underweight).[3] The multiple concurrent forms of undernutrition lead to a 12-fold elevated risk of child mortality compared to healthy children.[4] Also globally, around 3% of children under five years were estimated to exhibit both stunting and wasting.[5] The effect of stunting, wasting, and underweight on mortality through reduced muscle mass suggests that young infants and children are especially vulnerable to undernutrition, particularly the multiple concurrent forms.[6] A reduced muscle or fat mass (a common mechanism involving limbs being both thinner and shorter) increases the risk of death considerably when wasting and stunting are present in the same child.[6] Fat stimulates the necessary energy to maintain the immune system so a undernourished child with low muscle mass and reduced function of key organs, such as the heart, kidney, and immune system, is more likely to die during an acute food shortage.[6] Overall, undernutrition reduces the immunological capacity of a child to defend against diseases and recurrent infections, such as diarrhea, pneumonia, malaria, measles, and AIDS, that leads to inconsistent growth and development both mentally and physically.[7 8]

Child undernutrition is a global health problem and is particularly prevalent in low-and middle-income countries. In spite of a decline over the years, the rate of undernutrition in Bangladesh is still among the highest in the world, with estimates putting around 40% of children aged under 5 in that category.[9 10] The risk factors leading to child undernutrition are still being debated in Bangladesh and other developing countries. Key risk factors are poor maternal education, low socioeconomic status, low birth weight, poor feeding practices, frequent infections, inadequate access to health care, and water and sanitation.[7 11-13] However, these risk factors were identified using conventional disaggregated indicators (e.g., stunting, wasting and underweight). The findings from these studies are not sufficient to enable formulation of complete policy initiatives as many children in Bangladesh (around 30%) and

For peer review only - http://bmjopen.bmj.com/site/about/guidelines.xhtml

those in other developing countries are suffering from multiple concurrent forms of undernutrition that lead to a high mortality rate among children.[14]

Those factors associated with the single form and multiple concurrent forms of undernutrition have not been previously identified in Bangladesh. Chowdhury et al. (2020) tried to link several socio-demographic factors with single and multiple forms of severe undernutrition.[14] However, the study appended multiple datasets of previous national surveys that did not represent the current scenario nor the overall burden of single and multiple concurrent forms of undernutrition. Drivers or factors associated with both these forms of undernutrition have not yet been fully identified in Bangladesh or in other developing countries. According to recent research findings, age, sex, and food insecurity are linked to multiple concurrent forms of undernutrition.[4 15 16] On the other hand, factors associated with the single form have not been widely investigated in developing countries, including Bangladesh. Studies on single and multiple concurrent forms of undernutrition might present different results from those grounded in conventional disaggregated indicators. The present study aims to differentiate between the risk factors of both the multiple concurrent forms and a single form of undernutrition among under 5-year-old children in Bangladesh using a more recent nationally representative population-based survey. This study might therefore be useful for policymakers when addressing the needs of more nutritionally vulnerable children.

Methodology

Study setting

Bangladesh is a densely populated country of 165 million people, with 14 million of them under 5 years of age.[17] The country is divided into eight administrative divisions, namely, Barishal, Chattogram, Dhaka, Khulna, Mymensingh, Rajshahi, Rangpur, and Sylhet. Each division is divided into zilas (districts) and each zila into upazilas (sub-districts). The urban areas in an upazila are further divided into wards, and these wards are further subdivided into mohallas (suburbs). Urban areas are also classified into two groups: city corporations and areas other than city corporations. Rural areas in an upazila are divided into union parishads (villages) and then further subdivided into mouzas (small villages). Overall, the divisions separate the country into rural and urban areas with 54% of households in rural areas.[10]

Management of undernutrition in Bangladesh

For peer review only - http://bmjopen.bmj.com/site/about/guidelines.xhtml

The rate of undernutrition in Bangladesh is among the highest in the world. The country has developed facility-based care (therapeutic care for malnourished children with complications) and community-based care (undernourished children without complications and children who have been discharged from facility-based inpatient care) for the management of undernourished children.[18 19] In line with this, Bangladesh's government has also introduced several policies and strategies to help deal with the rate of undernutrition. For example, in 1997, the Bangladesh National Nutrition Council developed a policy called the Bangladesh National Plan of Action for Nutrition (NPAN) to improve the nutritional status and quality of life of the people of Bangladesh.[20] The NPAN was approved by the Food Planning and Monitoring Committee in August 2008 to ensure an adequate and stable supply of safe and nutritious food, to increase people's purchasing power and access to food and thereby provide proper nutrition for all individuals, especially women and children.[21] The government of Bangladesh has again taken the initiative to incorporate nutrition into public health and family planning services to improve the country's nutrition situation. The adoption of the National Nutritional Policy 2015 reflects the commitment of the State to improve the nutritional status and quality of life of the population especially for disadvantaged groups, including mothers, adolescent girls, and children.[22] A new holistic policy called "Food and Nutrition Security Policy" has also been adopted to promote the use of a "nutrition lens". This policy will help develop multi-sectoral inter-linked interventions that will improve nutritional outcomes and cover the target period 2020-2030 in synchronization with the Sustainable Development Goal (SDG).[23]

Data source

After excluding missing information regarding selected variables, a total of 7,661 children aged under 5, who were born in January 2013 or later, were included in this study with data taken from the Bangladesh Demographic Health Surveys (BDHS) 2017-18 (Figure 1). The BDHS 2017-18 data is nationally representative with a 99% response rate that includes data from adults and children (both male and female) regarding demographic and social characteristics and health and nutritional indicators to monitor a wide range of populations. The data collection was started on 24 October 2017 and ended on 15 March 2018.[10] This survey was funded by the United States Agency for International Development (USAID) and conducted by the National Institute of Population Research and Training (NIPORT) under the Ministry of Health and Family Welfare, Bangladesh. All survey-related issues were

For peer review only - http://bmjopen.bmj.com/site/about/guidelines.xhtml

implemented by a Bangladeshi research organization 'Mitra and Associate' with technical support from the ICF International of Calverton, Maryland, USA. The 2017-18 BDHS was reviewed and approved by the ICF Macro Institutional Review Board (USA).[10]

These surveys were based on multistage stratified sampling techniques of households. At the first stage, 675 primary sampling units (PSUs) were selected (250 PSU from urban and 425 PSU from rural) based on enumeration areas (clusters) from the census survey 2011 designed by the Bangladesh Bureau of Statistics using probability proportional to size technique. Then, a systematic sample of 30 households from each PSU was selected at the final stage using an equal probability systematic sampling technique.[10] This multistage sampling technique, including its sampling weight, helps reduce potential sampling bias.[10] In the BDHS data, sample weights were calculated in each sampling stage, each cluster and stratum were considered that had been adjusted for non-response to obtain the final standard weights.[10] In addition, all ever-married women aged 15-49 years from the pre-selected households were interviewed without replacement and change in the implementing stage to prevent selection bias.[10] Informed consent was obtained verbally from each participant to collect information about them and their children before enrolling in the study. A significant number of the study sample was illiterate, so verbal consent was considered the most suitable option to confirm participation.[10] Each BDHS used a standard questionnaire and the details, including sample design, data collection procedure, and other issues, are discussed elsewhere.[10]

Outcome variables and operational definitions

The outcomes of the study involved the identification of multiple concurrent forms and single form of undernutrition among children aged under 5 years. A child was considered to be undernourished or stunted (short stature for age), wasted (dangerously thin), and underweight (low weight for age) if the height-for-age, weight-for-height, and weight-for-age indices were two standard deviations (SDs) or more below the respective median of the WHO reference population.[24] A child was considered to have multiple concurrent forms of undernutrition when stunting and underweight and wasting and underweight, or three forms of undernutrition (stunting, wasting, and underweight) were present in the same child (Figure 2). Also, a child with a single form of undernutrition (in a composite manner) included a child living with stunting, wasting, or underweight only (Figure 2). A detailed variable scale of measurement with missing information is clearly defined in online supplemental table 1. Children were categorized 0 for 'normal' and 1 for 'undernutrition' for each of the three

For peer review only - http://bmjopen.bmj.com/site/about/guidelines.xhtml

indicators. After that, the values of all three indicators were added, which resulted in a score ranging from 0 to 3. The scores were again re-categorized as 0 for normal, 1 for a single form of undernutrition, and 2 to 3 for multiple concurrent forms of undernutrition. Detailed classifications and coding of outcome variables used in this study are clearly defined in online supplemental table 2.

Independent variables

Since 1990, the UNICEF conceptual framework on undernutrition has guided interventions from multi-sectoral and multidimensional perspectives.[25] This framework included the basic, underlying, and immediate causes of undernutrition. The basic causes address social, economic, environmental, and political issues that lead to the lack of or unequal distribution of financial, human, physical, social, and natural resources. The underlying causes focus on household food security, adequate care, and feeding practices, access to health services, and residing in a healthy environment. The basic and underlying causes lead to immediate causes at the individual level through inadequate food intake and disease.[25]

The candidate variables (potential causes) considered for analysis in the present study were based on relevant previous literatures.[7 26-30] These variables include maternal age in years (15-19, 20-24, 25-29, 30-34, 35-39, ≥40); parents' education (both parents uneducated, only father was uneducated when mother was educated, only mother was uneducated when father was educated, both parents educated); mother's current working status (currently not working, currently working); mother's underweight status ((normal/average (≥18.5 kg/m^2), underweight (<18.5 kg/m^2)); status of mother's antenatal and postnatal care (not received, received); mother's experience of inmate partner violence (IPV) (not experienced, experienced): a wife being beaten by partner if she went out without telling him or/and neglected the children or/and she ever argued with her partner or/and burned food or/and refused to have sex);[10] mothers' decision-making autonomy (not experienced, experienced): a woman who usually decides by herself/jointly with husband at least on her healthcare or on large household purchases or visits to family or relatives);[10] father's occupation (currently not working, manual labourer, professional and businessman); source of drinking water (improved, unimproved),[31] use of solid waste in cooking (solid, non-solid); type of toilet facility (improved, unimproved),[31] mass media exposure (no, yes: exposed to either radio, television, newspapers, or magazines at least once a week); wealth index (integrating household asset ownership and access to drinking water and sanitation)[10] and place of

For peer review only

For peer review only - http://bmjopen.bmj.com/site/about/guidelines.xhtml

residence (urban, rural). Moreover, various factors related to children, such as age, birth order, and birth weight status,[32-34] recent morbidity status (the child had at least one morbid condition out of diarrhea, fever, and cough in the two weeks preceding the survey)[10] were included. Detailed variable scales of measurement with missing information used in this study is clearly defined in online supplemental table 3, 4 & 5 .

Patient and public involvement

Patients or the public were not involved in the design, conduct, or interpretation of the study.

Statistical analysis

The background characteristics of the children and their parents were calculated using descriptive statistics. Crosstab analysis and Bivariate analysis (Chi-square test) were used to determine the prevalence/percentages and associations (*p* values) between the selected independent variables and outcomes. Further, all independent variables found to be significant in bivariate analysis were simultaneously entered into the stepwise (backword selection) multivariate regression models for adjustment to help identify risk factors for multiple concurrent forms and single form of undernutrition and when the base outcome (reference category) was normal (various forms of undernutrition vs normal/healthy children). The findings from logistic regression analysis were assessed using odds ratio (OR) and confidence interval (CI). The significance level was set at p <0.25 (2-tailed) in bivariate analyses instead of the traditional cut off point 0.05 that can fail in identifying variables known to be important.[35] On the other hand, the significance level was set at p <0.05 (2-tailed) in multivariate analyses. The data were analyzed by controlling cluster (PSU) and stratum (urban area: city corporation area and other than city corporation area and rural area) with sampling weights that represent the whole country, both urban and rural areas, to ensure the precision of the estimates. Multicollinearity was checked by examining the standard errors (SEs) of regression coefficients in the logistic regression analyses. An SE >2.0 indicates multicollinearity among the independent variables.[36] The SEs for the independent variables in the adjusted models for each outcome were <1, indicating an absence of multicollinearity. Stata version 14.2 (StataCorp LP, College Station, Texas) was used for all analyses considering the complex nature of the sampling weight of the BDHS. To adjust sampling weight in the analysis, the Stata command "svyset" was used.

For peer review only - http://bmjopen.bmj.com/site/about/guidelines.xhtml

For peer review only

Results

Table 1 shows that approximately 7% (both parents uneducated: 3.8% and only mothers were uneducated: 3.2%) of mothers had no formal education; 15% of mothers were reported underweight; more than 90% of mothers received antenatal care; one-third (33%) did not receive postnatal care and 18% experienced IPV. Around 42% of children were from poor households with 66% living in rural areas. A similar percentage distribution of children (approximately 20%) was observed among the various age categories, and 52% of them were male (Table 1).

Prevalence of multiple concurrent forms and single form of undernutrition

In Bangladesh, the overall prevalence of child undernutrition was 38%. The overall prevalence of stunting was 31%; wasting was 8% and underweight was 22% (Figure 2). The prevalence of multiple concurrent forms (from Figure 1, stunting and underweight: 13.5%; wasting and underweight: 3.1%; and stunting, wasting, underweight: 2.7%) and single form (from Figure 1, only stunting: 14.3%, only wasting: 2.1% and only underweight: 2.4%) of undernutrition among under 5 year old children were approximately estimated at 19%. Further, the prevalence of multiple concurrent forms of undernutrition was significantly higher among children of parents with no formal education (37%) when compared to educated parents (17%), children of underweight mothers (about 30%), children with fathers who were manual labourers (28%), children were ≥ 4 birth order (about 28%), and children born with low birth weight (27%) (Table 2).

The single form of undernutrition was highly prevalent among children of fathers with no formal education when mothers are educated (23%); children in the age group 12-35 months (23%); those from the poorest section of society (22%) when compared to the richest (14%); of underweight mothers and households that had no mass media exposure (21%); of fathers who were manual labourers and households with unimproved toilet facilities (21%) and children born with low birth weight (20%) (Table 3).

Risk factors

The key risk factors of multiple concurrent forms of undernutrition were children born with low birth weight (when compared to healthy weight children) [AOR: 3.76, 95% CI: 2.78,

For peer review only - http://bmjopen.bmj.com/site/about/guidelines.xhtml

5.10, p<0.001]; children in the age group 24-35 months (when compared to children in the age group 0-11 months) [AOR: 2.70, 95% CI: 2.20, 3.30, p<0.001]; children in the lowest socioeconomic quintile (when compared to the highest socioeconomic quintile) [AOR: 2.57, 95% CI: 2.05, 3.23, p<0.001]; parents with no formal education (when compared to educated parents) [AOR: 2.03, 95% CI: 1.53, 2.71, p<0.001]; and children with fathers who were currently unemployed (when compared to businessman fathers) [AOR: 1.98, 95% CI: 1.07, 3.16, p=0.021] (Table 4).

The risk factors for a single form of undernutrition include children in the age group 24-35 months (when compared to children in the age group 0-11 months) [AOR: 1.94, 95% CI: 1.61, 2.34, p<0.001]; children in the lowest socioeconomic quintile (when compared to the highest socioeconomic quintile) [AOR: 1.79, 95% CI: 1.45, 2.21, p<0.001]; children born with low birth weight (when compared to healthy weight children) [AOR: 1.52, 95% CI: 1.11, 2.08, p=0.008]; children of fathers who are manual labourers (when compared to businessman fathers) [AOR: 1.36, 95% CI: 1.16, 1.59, p<0.001]; and children of fathers with no formal education when mothers are educated (when compared to educated parents) [AOR: 1.35, 95% CI: 1.12, 1.63, p=0.002] (Table 4).

Discussion

The prevalence of multiple concurrent forms and a single form of undernutrition among children aged under 5 in Bangladesh was nearly equal, each accounting for 19%. One out of five children suffered from both cases of undernutrition. This high figure is a concern when comparing it with developing countries from Africa and South America, such as Ethiopia, Malawi and Argentina where the prevalence of concurrent forms of undernutrition among children age under 5 stood respectively at 26%, 12%, and 2%. The single form of undernutrition was respectively 23%, 39%, and 5%.[37-39] Asian countries such as Yemen and India are struggling with a high burden of both forms of undernutrition (multiple concurrent forms of undernutrition: 48% and 39% respectively; single of undernutrition: 21% and 24% respectively).[40 41] The findings indicate that Bangladesh has not still achieved sustainable improvement in reducing child undernutrition. One reason could be poor understanding of the multi-sectoral approach involving a lack of coordination among key government sectors, such as between health, agriculture, education, urban and local development to address the issue of undernutrition, and poor linkage between key institutions, for example, government

For peer review only - http://bmjopen.bmj.com/site/about/guidelines.xhtml

institutions, academic, research and training institutions, and national/international non-governmental organizations.[42]

This study reveals that mothers with no formal education in Bangladesh live in less protective environments. Three out of five children (65.4%) of mothers with no formal education and two out of five children (37.4%) experienced multiple concurrent forms of undernutrition. Also, three out of ten children (27.3%) born with low birth weight experience multiple concurrent forms of undernutrition. Further, the odds of multiple concurrent forms of undernutrition were 3.37 times higher among children born with a low birth weight than the children with a healthy birth weight. Previous studies in Bangladesh, Pakistan, Nepal, Malawi, Mexico, and Iran also reported children born with low birth weight were more likely to experience malnutrition regardless of multiple concurrent forms and single form.[43-47] Children born with low birth weight generally increase their height and weight by small increments and thus they may remain shorter and lighter and might be severely malnourished without adequate nutritional support.[48] Children with low birth weights are often born to uneducated and underweight mothers.[49 50] Maternal/parental illiteracy is associated with poor maternal healthcare access, and caregiving provided to children often leads to an adverse nutritional outcome for mothers and children.[51] This study shows that the odds of multiple concurrent forms of undernutrition were respectively 2.03 and 1.91 times higher among children of mothers with no formal education and among underweight mothers. Numerous studies assessed these factors as key determinants of child undernutrition using disaggregated indicators and need more assessment to estimate the magnitude of risks in case of multiple concurrent forms and single form of undernutrition.[7 11]

Other key risk factors of multiple concurrent forms of undernutrition include children in the older age group (3 years and above); those in the lowest socioeconomic quintile; have fathers who are currently unemployed that differs by a single form of undernutrition, to some extent, additionally with children in the younger age group (3 years and below); have mothers with no formal education (when fathers are educated) and fathers that are labourers. An assessment of the risk difference between educated and uneducated parents and the highest and lowest socioeconomic quintile for multiple concurrent forms and a single form of undernutrition indicates higher educational and wealth inequalities for the multiple concurrent forms of undernutrition. Further, the level of parental education and economic hardship is not markedly responsible for suffering from a single form of undernutrition

For peer review only - http://bmjopen.bmj.com/site/about/guidelines.xhtml

among children aged under 5. Earlier studies in Bangladesh and other South Asian countries highlighted parental education and wealth index as key risk factors of child undernutrition regardless of multiple concurrent forms and single form. Still, the studies need more precise estimation that could help policy makers introduce context-specific actions for reducing undernutrition.[7 11 28 30 52] Women who have low social status and less education have very limited influence on household decision-making that might influence child nutritional status. On the other hand, a large community in Bangladesh are concerned with broader issues of chronic poverty, and this would not reflect the improvement of undernutrition at a community level.[52] To reduce under 5 year old child undernutrition, it is recommended that the participation of women and girls in education is increased, especially in rural areas, minimizing socioeconomic inequality by raising the income-generating sectors, and strengthening the nutrition-specific health programmes.

Children in the older age group (3 years and above) have a high risk of multiple concurrent forms of undernutrition, while children in the younger age group (3 years and below) were more likely to have a single form of undernutrition. Many studies conducted in Bangladesh, Nepal, Pakistan, Ethiopia, and Congo have consistently addressed age and older children as having a higher risk of being undernourished without classifying the two conditions.[26-30] In India, younger children, were found to have a higher likelihood of being undernourished.[53] Older children receive less protection in times of adverse nutritional outcomes due to poor per capita food distribution among family members in large households when they tend to be allocated less food than will meet their energy requirements with regards to their age.[54] The risk of poor parental education on multiple concurrent forms of undernutrition differs by paternal illiteracy (when mothers are educated) on a single form of undernutrition in identifying the impact of education on undernutrition. This study shows that paternal education was associated with a single form of undernutrition among children under 5 in Bangladesh. Evidence suggests that the impact of paternal education on undernutrition has not been widely addressed while maternal education has a more significant effect on child undernutrition than paternal education.[53] Parental education is associated with positive attitudes and behaviours that can reduce the incidence of child undernutrition to some extent. It needs to be recognised that the complex nature of modern health care messages and behavioural changes will not be effectively understood or practised by mothers with only the most basic literacy and numeracy skills.[55] Reducing the gender gap for enrolment in universal upper secondary education may help towards tackling the burden of all undernutrition.

For peer review only - http://bmjopen.bmj.com/site/about/guidelines.xhtml

This study also indicates that children of unemployed fathers are more likely to experience multiple concurrent forms of undernutrition; conversely, children of fathers who were labourers are less protected in the case of a single form of undernutrition. Additionally, three out of ten children experienced multiple concurrent forms of undernutrition, with 21% experiencing a single form. The effects of paternal unemployment on child undernutrition have not been widely explored, except for a study conducted in China that found it had a significant impact.[56] Conversely, those children of fathers that had low paid jobs (labourer, farmer, etc.) had a greater risk of suffering undernutrition.[11 14] A loss of employment or low paid jobs are typically associated with a lower income available for spending on market goods, including healthcare, non-household childcare, and nutritious consumption.[56] Increasing investment in healthcare at the domestic level could help in tackling undernutrition.

This study strongly suggests that policy implications or interventions from the perspectives of developing countries, such as Bangladesh, are needed to successfully prevent and treat both forms of undernutrition, particularly multiple concurrent forms and based on evidence and a broad-spectrum. Firstly, factors associated with undernutrition should be considered in order to formulate an evidence-based strategy for reducing undernutrition, with the emphasis on the multiple concurrent forms. Routine national and subnational level nutrition surveys, such as Multiple Indicator Cluster Surveys (MICSs), need to be modified to include multiple concurrent forms to aid in the development of programmes and policy decision-making.[57] Routine monitoring of the prevalence of multiple concurrent forms would also be required to inform effective detection and treatment.[57] Community engagement and screening for multiple concurrent forms of undernutrition could also be expanded in innovative ways by enrolling additional expertise and resources.[58] Innovative and early markers should be developed to predict, identify, and monitor children for short-term and long-term consequences.[59] Maternal factors from adolescence through pregnancy need to be scrutinised in order to identify those that can adversely affect utero and postnatal children living with three forms of undernutrition.[59] Therapeutic interventions (e.g., ready-to-use therapeutic foods) must be reviewed and adjusted to ensure that children at the highest risk of mortality are included due to multiple concurrent forms of undernutrition. Comprehensive nutrition programmes must be developed for pursuing SDG 2.2 in order to end undernutrition in all its forms by 2030.[59]

For peer review only - http://bmjopen.bmj.com/site/about/guidelines.xhtml

This study analyzed data from a national BDHS survey 2017-2018. The findings can therefore be applied to the whole country and to low- and middle-income countries whose socio-demographic characteristics and healthcare settings are similar to Bangladesh. Another strength in this study was the appropriate application of statistical tools that might help in understanding the findings. The multistage sampling technique, for instance, including its sampling weight, helped reduce potential selection bias. There were some limitations. For example, the cross-sectional nature of the data did not establish a causal relationship between risk factors and outcome variables. All three indicators (e.g., stunting, wasting, and underweight) were used to formulate the study outcomes (single and multiple concurrent forms of undernutrition) without considering distinct associated factors that might affect the results. This study did not control other important indicators, such as a child's diet pattern, parents' behaviour of parents or immunization and ethnicity, due to lack of availability and missing values across the surveys. Another limitation arises due to recall bias or information bias as a result of self-reporting of age, education, occupation and household assets for example.

Conclusion

One out of five children in Bangladesh aged under 5 years is suffering multiple concurrent forms and a single form of undernutrition. Children born with low birth weight, in the lowest socioeconomic quintile, and in the older age group (3 years and above) were identified as key risk factors for multiple concurrent forms of undernutrition. Those children in the younger age group (3 years and below), and mothers with no formal education had significant effects on the single dimension of undernutrition. Parental education, father's occupation, age, and birth order are the main differentiating risk factors between multiple concurrent forms and single form of undernutrition. The findings highlight factors that should be considered in order to formulate an evidence-based strategy to reduce undernutrition among under 5 year old children. In addition, concerted efforts are essential for developing strong collaboration among different sectors, such as between government, non-government, educational, social, cultural, and religious institutions, in order to improve nutritional status. The findings from this study may be transferable to other low- and middle-income countries but further study is warranted in this regard.

For peer review only - http://bmjopen.bmj.com/site/about/guidelines.xhtml

Ethical statements

The BDHS was reviewed and approved by the ICF Macro Institutional Review Board (USA), which complies with all of the requirements of 45 CFR 46 "Protection of Human Subjects". The Bangladesh DHS was also reviewed and approved by the National Research Ethics Committee of the Bangladesh Medical Research Council (Dhaka, Bangladesh). Informed consent was obtained verbally from each participant and their intimate partners (all ever-married women aged 15–49 years old) prior to being enrolled in the study. A significant number of the study sample was illiterate, so verbal consent was considered the most suitable option to confirm participation. The BDHS surveys also included samples of very young children (under five-year-old children either born in 2014 or later) in the data collection, and so mothers of these samples were asked to provide verbal consent on behalf of their children.

Acknowledgement: The authors thank the MEASURE DHS project for their support and free access to the original data and the Department of Public Health, First Capital University of Bangladesh, enabling the research scope.

Funding: This research received no specific grant from any institutions.

Competing interests: The authors declare that they have no competing interests.

Authors' contribution: MRKC conceptualized the basic idea for the study, performed the statistical analysis together with MSI and RK. MRKC and SI prepared data for analysis. MRKC and MK prepared the first draft of the manuscript. HTAK and MR critically revised the manuscript for intellectual content. All authors have reviewed and approved the final manuscript.

Checklist for the appropriate reporting statement: Add STROBE checklist as supplementary.

A patient consent form: Not applicable

Data sharing statement: Authors are responsible for providing the statistical code for software application, on request.

Word count: 4,657 (excluding abstract, strength and limitation, reference, tables and figures)

For peer review only - http://bmjopen.bmj.com/site/about/guidelines.xhtml

Supplementary and raw data: Supplementary table 1, 2, 3, 4 & 5 have been submitted with the main manuscript. The data underlying the results presented in the study are publicly accessible and available from the DHS website (https://dhsprogram.com/data/available-datasets.cfm). The name of the dataset is Bangladesh Demographic and Health Survey (BDHS) 2017-18.

For peer review only

For peer review only - http://bmjopen.bmj.com/site/about/guidelines.xhtml

Reference

1. WHO. Malnutrition. *World Health Organization (WHO) Switzerland: Geneva* 2021;Retrieved 22 August, 2021, from https://www.who.int/news-room/fact-sheets/detail/malnutrition
2. WHO. Underweight among children under 5 years of age (number in millions) (JME). The Global Health Observatory-Explore a world of health data. *World Health Organization (WHO) Switzerland: Geneva* 2021;Retrieved 22 August, 2021, from https://www.who.int/data/gho/data/indicators/indicator-details/GHO/gho-jme-underweight-numbers-(in-millions
3. McDonald CM, Olofin I, Flaxman S, et al. The effect of multiple anthropometric deficits on child mortality: meta-analysis of individual data in 10 prospective studies from developing countries. *The American journal of clinical nutrition* 2013;97(4):896-901.
4. Myatt M, Khara T, Schoenbuchner S, et al. Children who are both wasted and stunted are also underweight and have a high risk of death: a descriptive epidemiology of multiple anthropometric deficits using data from 51 countries. *Archives of Public Health* 2018;76(1):1-11.
5. Khara T, Mwangome M, Ngari M, et al. Children concurrently wasted and stunted: A meta-analysis of prevalence data of children 6–59 months from 84 countries. *Maternal & child nutrition* 2018;14(2):e12516.
6. Briend A, Khara T, Dolan C. Wasting and stunting—similarities and differences: policy and programmatic implications. *Food and nutrition bulletin* 2015;36(1_suppl1):S15-S23.
7. Chowdhury MRK, Rahman MS, Khan MMH, et al. Risk factors for child malnutrition in Bangladesh: a multilevel analysis of a nationwide population-based survey. *The Journal of pediatrics* 2016;172:194-201. e1.
8. De P, Chattopadhyay N. Effects of malnutrition on child development: Evidence from a backward district of India. *Clinical Epidemiology and Global Health* 2019;7(3):439-45.
9. DI. 2020 Global Nutrition Report: Action on equity to end malnutrition. Bristol, UK: Development Initiatives. *Development Initiatives* 2020
10. NIPORT. Demographic and Health Survey 2017–2018. *National Institute of Population Research and Training* 2019
11. Islam MR, Rahman MS, Rahman MM, et al. Reducing childhood malnutrition in Bangladesh: the importance of addressing socioeconomic inequalities. *Public health nutrition* 2020;23(1):72-82.
12. Islam MS, Biswas T. Prevalence and correlates of the composite index of anthropometric failure among children under 5 years old in Bangladesh. *Maternal & child nutrition* 2020;16(2):e12930.
13. Adhikari RP, Shrestha ML, Acharya A, et al. Determinants of stunting among children aged 0–59 months in Nepal: findings from Nepal Demographic and Health Survey, 2006, 2011, and 2016. *BMC nutrition* 2019;5(1):1-10.
14. Chowdhury MRK, Khan HT, Mondal MNI, et al. Socio-demographic risk factors for severe malnutrition in children aged under five among various birth cohorts in Bangladesh. *Journal of Biosocial Science* 2021;53(4):590-605.
15. Garenne M, Myatt M, Khara T, et al. Concurrent wasting and stunting among under-five children in Niakhar, Senegal. *Maternal & child nutrition* 2019;15(2):e12736.
16. Schoenbuchner SM, Dolan C, Mwangome M, et al. The relationship between wasting and stunting: a retrospective cohort analysis of longitudinal data in Gambian children from 1976 to 2016. *The American journal of clinical nutrition* 2019;110(2):498-507.
17. UN. United Nations (UN). Department of Economic and Social Affairs, Population Division (2019). World Population Prospects 2019. Retrieved 16 August, 2021, from https://population.un.org/wpp/. 2019

For peer review only - http://bmjopen.bmj.com/site/about/guidelines.xhtml

18. IPHN. National Guidelines for the Facility-based Management of Children with Severe Acute Malnutrition in Bangladesh. *Institute of Public Health Nutrition (IPHN) Director General of Health Services Ministry of Health and Family Welfare Government of People's Republic of Bangladesh* 2017;Retrieved 22 August, 2021, from https://scalingupnutrition.org/wp-content/uploads/2013/02/Bangladesh_NationalFoodPolicyPlanofAction_2008_2015.pdf
19. IPHN. National Guidelines for Community Based Management of Acute Malnutrition in Bangladesh. *Institute of Public Health Nutrition (IPHN) Director General of Health Services Ministry of Health and Family Welfare Government of People's Republic of Bangladesh* 2017;Retrieved 22 August, 2021, from https://www.humanitarianresponse.info/sites/www.humanitarianresponse.info/files/documents/files/cmam_guideline_revised_10_jul17.pdf
20. WHO. Bangladesh National Plan of Action for Nutrition (NPAN). Bangladesh National Nutrition Council *World Health Organization (WHO) Switzerland: Geneva Organization Global database on the implementation of nutrition action* 1997;Retrieved 22 August, 2021, from https://extranet.who.int/nutrition/gina/en/node/8230
21. FPMU. National Food Policy Plan of Action (2008-2015). *Food Planning and Monitoring Unit (FPMU) Ministry of Food and Disaster Management Dhaka, Bangladesh* 2008;Retrieved 22 August, 2021, from https://scalingupnutrition.org/wp-content/uploads/2013/02/Bangladesh_NationalFoodPolicyPlanofAction_2008_2015.pdf
22. NNP. National Nutrition Policy 2015. Nutrition Is the Foundation for Development. *National Nutrition Policy (NNP)* 2015;Retrieved August 22, 2021, from http://extwprlegs1.fao.org/docs/pdf/bgd152517.pdf
23. FAO. National Food and Nutrition Security Policy of Bangladesh (NFNSP). *Food and agriculture organization (FAO) in Bangladesh* 2019;Retrieved 22 August, 2021, from http://fpmu.gov.bd/agridrupal/sites/default/files/file/policy/NFNSP-2019-Draft-English.pdf
24. WHO. WHO child growth standards: length/height-for-age, weight-for-age, weight-for-length, weight-for-height and body mass index-for-age: methods and development: World Health Organization 2006.
25. Reinhardt K, Fanzo J. Addressing chronic malnutrition through multi-sectoral, sustainable approaches: a review of the causes and consequences. *Frontiers in nutrition* 2014;1:13.
26. Kismul H, Acharya P, Mapatano MA, et al. Determinants of childhood stunting in the Democratic Republic of Congo: further analysis of Demographic and Health Survey 2013–14. *BMC public health* 2018;18(1):74.
27. Abera L, Dejene T, Laelago T. Magnitude of stunting and its determinants in children aged 6–59 months among rural residents of Damot Gale district; southern Ethiopia. *BMC research notes* 2018;11(1):557.
28. Adhikari RP, Shrestha ML, Acharya A, et al. Determinants of stunting among children aged 0–59 months in Nepal: findings from Nepal Demographic and health Survey, 2006, 2011, and 2016. *BMC nutrition* 2019;5(1):37.
29. Khanam M, Shimul SN, Sarker AR. Individual-, household-, and community-level determinants of childhood undernutrition in Bangladesh. *Health services research and managerial epidemiology* 2019;6:2333392819876555.
30. Khan S, Zaheer S, Safdar NF. Determinants of stunting, underweight and wasting among children< 5 years of age: evidence from 2012-2013 Pakistan demographic and health survey. *BMC Public Health* 2019;19(1):358.
31. Croft T, Marshall A, Allen C. Guide to DHS statistics. Rockville, Maryland, USA: ICF; 2018, 2018.
32. Sreeramareddy CT, Shidhaye RR, Sathiakumar N. Association between biomass fuel use and maternal report of child size at birth-an analysis of 2005-06 India Demographic Health Survey data. *BMC public health* 2011;11(1):1-10.

For peer review only - http://bmjopen.bmj.com/site/about/guidelines.xhtml

33. Khanal V, Sauer K, Karkee R, et al. Factors associated with small size at birth in Nepal: further analysis of Nepal Demographic and Health Survey 2011. *BMC pregnancy and childbirth* 2014;14(1):1-9.
34. Haque SR, Tisha S, Huq N. Poor birth size a badge of low birth weight accompanying less antenatal care in Bangladesh with substantial divisional variation: evidence from BDHS-2011. *Birth* 2015;1(2476):33.8.
35. Bursac Z. Gauss CH Williams DK, & Hosmer DW (2008). *Purposeful selection of variables in logistic regression Source Code for Biology and Medicine*;3:17.
36. Chan Y. Biostatistics 202: logistic regression analysis. *Singapore medical journal* 2004;45(4):149-53.
37. Endris N, Asefa H, Dube L. Prevalence of malnutrition and associated factors among children in rural Ethiopia. *BioMed research international* 2017;2017
38. Ziba M, Kalimbira AA, Kalumikiza Z. Estimated burden of aggregate anthropometric failure among Malawian children. *South African Journal of Clinical Nutrition* 2018;31(2):20-23.
39. Bejarano IF, Oyhenart EE, Torres MF, et al. Extended composite index of anthropometric failure in Argentinean preschool and school children. *Public health nutrition* 2019;22(18):3327-35.
40. Al-Sadeeq AH, Bukair AZ, Al-Saqladi A-WM. Assessment of undernutrition using Composite Index of Anthropometric Failure among children aged< 5 years in rural Yemen. *Eastern Mediterranean Health Journal* 2018;24(12)
41. Boregowda G, Soni G, Jain K, et al. Assessment of under nutrition using composite index of anthropometric failure (CIAF) amongst toddlers residing in Urban slums of Raipur City, Chhattisgarh, India. *Journal of clinical and diagnostic research: JCDR* 2015;9(7):LC04.
42. Saha KK, Billah M, Menon P, et al. Bangladesh National Nutrition Services: assessment of implementation status: World Bank Publications 2015.
43. Rahman MS, Howlader T, Masud MS, et al. Association of low-birth weight with malnutrition in children under five years in Bangladesh: do mother's education, socioeconomic status, and birth interval matter? *PloS one* 2016;11(6):e0157814.
44. Tiwari R, Ausman LM, Agho KE. Determinants of stunting and severe stunting among under-fives: evidence from the 2011 Nepal Demographic and Health Survey. *BMC pediatrics* 2014;14(1):1-15.
45. Varela-Silva MI, Azcorra H, Dickinson F, et al. Influence of maternal stature, pregnancy age, and infant birth weight on growth during childhood in Yucatan, Mexico: a test of the intergenerational effects hypothesis. *American Journal of Human Biology: The Official Journal of the Human Biology Association* 2009;21(5):657-63.
46. Ntenda PAM. Association of low birth weight with undernutrition in preschool-aged children in Malawi. *Nutrition journal* 2019;18(1):1-15.
47. Jahanihashemi H, Noroozi M, Zavoshy R, et al. Malnutrition and birth related determinants among children in Qazvin, Iran. *European journal of public health* 2017;27(3):559-62.
48. Doyle LW. Growth and respiratory health in adolescence of the extremely low-birth weight survivor. *Clinics in perinatology* 2000;27(2):421-32.
49. Bhowmik B, Siddique T, Majumder A, et al. Maternal BMI and nutritional status in early pregnancy and its impact on neonatal outcomes at birth in Bangladesh. *BMC pregnancy and childbirth* 2019;19(1):1-14.
50. Ntenda PAM, Chuang Y-C. Analysis of individual-level and community-level effects on childhood undernutrition in Malawi. *Pediatrics & Neonatology* 2018;59(4):380-89.
51. Lenters L, Wazny K, Bhutta ZA. Management of severe and moderate acute malnutrition in children. *Reproductive, maternal, newborn, and child health: disease control priorities 3rd edition Washington, DC: World Bank* 2016:205-23.
52. Nisbett N, Davis P, Yosef S, et al. Bangladesh's story of change in nutrition: Strong improvements in basic and underlying determinants with an unfinished agenda for direct community level support. *Global food security* 2017;13:21-29.

For peer review only - http://bmjopen.bmj.com/site/about/guidelines.xhtml

53. Sinha RK, Dua R, Bijalwan V, et al. Determinants of stunting, wasting, and underweight in five high-burden pockets of four Indian states. *Indian journal of community medicine: official publication of Indian Association of Preventive & Social Medicine* 2018;43(4):279.
54. Aiga H, Abe K, Andrianome VN, et al. Risk factors for malnutrition among school-aged children: a cross-sectional study in rural Madagascar. *BMC Public Health* 2019;19(1):1-13.
55. Alderman H, Headey DD. How important is parental education for child nutrition? *World development* 2017;94:448-64.
56. Pieters J, Rawlings S. Parental unemployment and child health in China. *Review of Economics of the Household* 2020;18(1):207-37.
57. Odei Obeng-Amoako GA, Myatt M, Conkle J, et al. Concurrently wasted and stunted children 6-59 months in Karamoja, Uganda: prevalence and case detection. *Maternal & child nutrition* 2020;16(4):e13000.
58. Imam A, Hassan-Hanga F, Sallahdeen A, et al. A cross-sectional study of prevalence and risk factors for stunting among under-fives attending acute malnutrition treatment programmes in north-western Nigeria: Should these programmes be adapted to also manage stunting? *International Health* 2021;13(3):262-71.
59. Wells JC, Briend A, Boyd EM, et al. Beyond wasted and stunted—a major shift to fight child undernutrition. *The Lancet Child & Adolescent Health* 2019;3(11):831-34.

For peer review only

For peer review only - http://bmjopen.bmj.com/site/about/guidelines.xhtml

Tables

Table 1

Background characteristics of the under-5 children

Factors	Number	Percentage (%)
**Total**	**7,661**	**100.0**
**Mothers' age (in years)**		
15-19	938	12.2
20-24	2,679	35.0
25-29	2,146	28.0
30-34	1,293	16.9
35-39	481	6.3
≥ 40	124	1.6
**Parents' education**		
Both parents were uneducated	294	3.8
Only father was uneducated	865	11.3
Only mother was uneducated	243	3.2
Both parents were educated	6,259	81.7
**Mother currently working**		
No	4,560	59.5
Yes	3,101	40.5
**Underweight mother**[a]		
No	6,535	85.3
Yes(<18.5 kg/m^2)	1,126	14.7
**Mothers received antenatal care (n=4,540)**		
No	363	8.0
Yes	4,177	92.0
**Mothers received postnatal care (n=4,535)**		
No	1,509	33.3
Yes	3,026	66.7
**Mothers experience IPV**[b]		
No	6,264	81.8
Yes	1,397	18.2
**Mothers' decision-making autonomy**[c]		
Not practiced	1,083	14.1
Practiced	6,578	85.9
**Father's occupation**		
Currently not working	73	1.0
Manual labourer	5,458	71.2
Professional	472	6.2
Businessman	1,658	21.6
**Source of water**		
Improved	6,658	86.9
Unimproved	1,003	13.1
**Type of toilet facility**		
Improved	4,359	56.9
Unimproved	3,302	43.1
**Solid waste use in cooking**		
No	2,218	28.9
Yes	5,443	71.1
**Mass media exposure**[d]		
No	4,890	63.8
Yes	2,771	36.2
**Wealth index** [e]		
Poorest	1,708	22.2
Poorer	1,545	20.2
Middle	1,381	18.0
Richer	1,533	20.0

For peer review only - http://bmjopen.bmj.com/site/about/guidelines.xhtml

Richest	1,494	19.5
**Place of residence**		
Urban	2,605	34.0
Rural	5,056	66.0
**Children's age (in months)**		
0-11 months	1,673	21.8
12-23 months	1,583	20.7
24-35 months	1,475	19.3
36-47 months	1,417	18.5
48-59 months	1,513	19.7
**Sex of child**		
Male	3,995	52.2
Female	3,666	47.8
**Birth order**		
One	2,902	37.9
Two	2,507	32.7
Three	1,297	16.9
Four and above	955	12.5
**Low birthweight [f](n=4,735)**		
No	1,815	38.3
Yes (<2.5 kg)	326	6.9
Not weighted	2,594	54.8
**Currently had disease [g]**		
No	4,043	52.8
Yes	3,618	47.2

[a]underweight measured as <18.5 kg/m^2

[b]a wife being beaten if she went out without telling her partner/ neglected the children/argued with her partner/burnt food/ was forced to have sex regardless of her consent

[c]a woman usually can decide by herself or jointly on her healthcare/large household purchases/visits to family or relatives

[d]exposed television/radio/newspaper/magazine to some extent

[e]integrating household asset ownership and access to drinking water and sanitation

[f]child's weight at birth measured as ≤2.5 kg

[g]child had at least one morbid condition out of diarrhea, fever and cough in the two weeks preceding the survey

For peer review only

For peer review only - http://bmjopen.bmj.com/site/about/guidelines.xhtml

Table 2

Prevalence of multiple concurrent forms of undernutrition among under-5 children

Factors	Multiple concurrent forms of undernutrition			
	No		Yes	
	Number (%)	Proportion (95% CI)	Number (%)	Proportion/prevalence (95% CI)
**Total**	6133	80.7 (79.5,81.8)	1528	19.3 (18.2-20.5)
**Mothers' age (in years)**				
15-19	755	81.1 (78.1-83.8)	183	18.9 (16.2-21.9)
20-24	2187	81.7 (79.9-83.4)	492	18.3 (16.6-20.1)
25-29	1697	79.8 (77.9-81.6)	449	20.2 (18.4-22.1)
30-34	1029	80.6 (77.8-83.2)	264	19.4 (16.8-22.2)
35-39	378	80.1 (75.8-83.8)	103	19.9 (16.2-24.2)
≥ 40	87	71.0 (61.1-79.2)	37	29.0 (20.8-38.9)
***p* values (Chi-square test)**		0.160		
**Parents' education**				
Both parents were uneducated	186	62.6 (55.7-69.0)	108	37.4 (31.0-44.3)
Only father was uneducated	644	74.4 (70.9-77.7)	221	25.6 (22.3-29.1)
Only mother was uneducated	168	72.0 (65.0-78.1)	75	28.0 (21.9-35.0)
Both parents were educated	5135	82.7 (81.5-83.8)	1124	17.3 (16.2-18.5)
***p* values (Chi-square test)**		<0.001		
**Mother currently working**				
No	3715	82.2 (80.7-83.6)	845	17.8 (16.4-19.3)
Yes	2418	78.4 (76.6-80.1)	683	21.6 (19.9-23.4)
***p* values (Chi-square test)**		0.0004		
**Underweight mother**				
No	5353	82.3 (81.0-83.5)	1182	17.7 (16.5-19.0)
Yes (<18.5 kg/m^2)	780	70.5 (67.5-73.3)	346	29.5 (26.7-32.5)
***p* values (Chi-square test)**		<0.001		
**Mothers received antenatal care**				
No	266	73.8 (68.7,78.4)	97	26.2 (21.6-31.3)
Yes	3476	84.0 (82.6,85.2)	701	16.0 (14.8-17.4)
***p* values (Chi-square test)**		<0.001		
**Mothers received postnatal care**				
No	1290	85.8 (83.7-87.7)	219	14.2 (12.3-16.3)
Yes	2448	81.7 (80.1-83.3)	578	18.3 (16.7-19.9)
		p=0.001		
**Mothers experienced IPV**				
No	5055	81.3 (80.1-82.5)	1209	18.7 (17.5-19.9)
Yes	1078	77.8 (75.2-80.2)	319	22.2 (19.8-24.8)
***p* values (Chi-square test)**		0.005		
**Mothers' decision-making autonomy**				
Not practiced	852	79.5 (76.7-82.1)	231	20.5 (17.9-23.3)
Practiced	5281	80.8 (79.5-82.1)	1297	19.2 (17.9-20.5)
***p* values (Chi-square test)**		0.370		
**Father's occupation**				
Currently not working	52	71.9 (58.6-82.2)	21	28.1 (17.8-41.4)
Manual labourer	4277	79.1 (77.7-80.4)	1181	20.9 (19.6-22.3)
Professional	425	90.4 (87.1-92.9)	47	9.6 (7.1-12.9)
Businessman	1379	84.0 (81.8-86.0)	279	16.0 (14.0-18.2)
***p* values (Chi-square test)**		*p*<0.001		
**Source of water**				
Improved	5309	80.3 (79.0-81.6)	1349	19.7 (18.4-21.0)
Unimproved	824	82.9 (80.1-85.4)	179	17.1 (14.6-19.9)
***p* values (Chi-square test)**		0.092		
**Type of toilet facility**				
Improved	3587	82.6 (81.1-84.0)	772	17.4 (16.0-18.9)
Unimproved	2546	78.1 (76.2-79.9)	756	21.9 (20.1-23.8)

For peer review only - http://bmjopen.bmj.com/site/about/guidelines.xhtml

For peer review only

			0.0001	
**Solid waste use in cooking**				
No	1888	84.7 (82.7-86.5)	330	15.3 (13.5-17.3)
Yes	4245	78.9 (77.5-80.3)	1198	21.1 (19.7-22.5)
***p* values (Chi-square test)**			<0.001	
**Mass media exposure**				
No	2095	76.2 (74.2-78.1)	676	23.8 (21.9-25.8)
Yes	4038	83.1 (81.7-84.3)	852	16.9 (15.7-18.3)
***p* values (Chi-square test)**			<0.001	
**Wealth index**				
Poorest	1248	73.6 (71.0-76.0)	460	26.4 (24.0-29.0)
Poorer	1172	77.2 (74.8-79.5)	373	22.8 (20.5-25.2)
Middle	1105	81.3 (78.6-83.7)	276	18.7 (16.3-21.4)
Richer	1266	82.8 (80.2-85.1)	267	17.2 (14.9-19.8)
Richest	1342	89.8 (87.7-91.5)	152	10.2 (8.5-12.3)
***p* values (Chi-square test)**			*p*<0.001	
**Place of residence**				
Urban	2152	82.8 (80.5-84.8)	453	17.2 (15.2-19.5)
Rural	3981	79.9 (78.5-81.3)	1075	20.1 (18.7-21.5)
***p* values (Chi-square test)**			0.032	
**Children's age (in months)**				
0-11 months	1454	88.0 (86.2-89.6)	219	12.0 (10.4-13.8)
12-23 months	1293	82.7 (80.5-84.7)	290	17.3 (15.3-19.5)
24-35 months	1141	77.6 (75.0-80.1)	334	22.4 (19.9-25.0)
36-47 months	1097	77.7 (75.0-80.2)	320	22.3 (19.8-25.0)
48-59 months	1148	76.1 (73.6-78.5)	365	23.9 (21.5-26.4)
***p* values (Chi-square test)**			<0.001	
**Sex of child**				
Male	3188	80.4 (78.8-82.0)	807	19.6 (18.0-21.2)
Female	2945	80.9 (79.3-82.4)	721	19.1 (17.6-20.7)
***p* values (Chi-square test)**			0.645	
**Birth order**				
One	2385	82.3 (80.5-83.9)	517	17.7 (16.1-19.5)
Two	2043	82.1 (80.3-83.7)	464	17.9 (16.3-19.7)
Three	1023	80.4 (78.0-82.6)	274	19.6 (17.4-22.0)
Four and above	682	72.4 (68.8-75.8)	273	27.6 (24.2-31.2)
***p* values (Chi-square test)**			<0.001	
**Low birth weight**				
No	1618	89.5 (87.7-91.0)	197	10.5 (9.0-12.3)
Yes (<2.5 kg)	230	72.7 (67.1-77.7)	96	27.3 (22.3-32.9)
Not weighted	2043	79.7 (77.8-81.4)	551	20.3 (18.6-22.2)
***p* values (Chi-square test)**			<0.001	
**Currently had disease**				
No	3277	81.5 (79.9-82.9)	766	18.5 (17.1-20.1)
Yes	2856	79.8 (78.1-81.4)	762	20.2 (18.6-21.9)
***p* values (Chi-square test)**			0.103	

No undenutrition for multiple concurrent forms includes healthy children and children with single form of undernutrition

CI, confidence interval

For peer review only

Table 3

Prevalence of single form of undernutrition among under-5 children

Factors	Single form of undernutrition			
	No		Yes	
	Number (%)	Proportion (95% CI)	Number (%)	Proportion/prevalence (95% CI)
**Total**	6205	81.1 (80.1-82.1)	1456	18.9 (17.9-19.9)
**Mothers' age (in years)**				
15-19	745	79.9 (76.8-82.7)	193	20.1 (17.3-23.2)
20-24	2174	81.2 (79.4-82.8)	505	18.8 (17.2-20.6)
25-29	1759	82.0 (80.1-83.8)	387	18.0 (16.2-19.9)
30-34	1031	79.9 (77.4-82.1)	262	20.1 (17.9-22.6)
35-39	391	81.0 (76.7-84.7)	90	19.0 (15.3-23.3)
≥ 40	105	85.7 (77.7-91.2)	19	14.3 (8.8-22.3)
***p* values (Chi-square test)**			0.533	
**Parents' education**				
Both parents were uneducated	233	80.9 (75.5-85.3)	61	19.1 (14.7-24.5)
Only father was uneducated	666	77.0 (73.9-79.8)	199	23.0 (20.2-26.1)
Only mother was uneducated	191	78.2 (71.3-83.8)	52	21.8 (16.2-28.7)
Both parents were educated	5115	81.8 (80.6-82.9)	1144	18.2 (17.1-19.4)
***p* values (Chi-square test)**			0.017	
**Mother currently working**				
No	3704	81.5 (80.0-82.8)	856	18.5 (17.2-20.0)
Yes	2501	80.5 (78.9-82.1)	600	19.5 (17.9-21.1)
***p* values (Chi-square test)**			0.396	
**Underweight mother**				
No	5323	81.5 (80.3-82.6)	1212	18.5 (17.4-19.7)
Yes (<18.5 kg/m^2)	882	78.8 (76.1-81.2)	244	21.2 (18.8-23.9)
***p* values (Chi-square test)**			0.053	
**Mothers received antenatal care**				
No	279	77.5 (72.5-81.8)	84	22.5 (18.2-27.5)
Yes	3334	79.8 (78.5-81.2)	843	20.2 (18.8-21.5)
***p* values (Chi-square test)**			0.327	
**Mothers received postnatal care**				
No	1210	80.5 (78.1-82.8)	299	19.5 (17.2-21.9)
Yes	2398	79.2 (77.6-80.7)	628	20.8 (19.3-22.4)
			0.335	
**Mothers experienced IPV**				
No	5071	81.2 (80.0-82.3)	1193	18.8 (17.7-20.0)
Yes	1134	80.8 (78.5-82.9)	263	19.2 (17.1-21.5)
***p* values (Chi-square test)**			0.744	
**Mothers' decision-making autonomy**				
Not practiced	881	82.2 (79.6-84.5)	202	17.8 (15.5-20.4)
Practiced	5324	80.9 (79.8-82.0)	1254	19.1 (18.0-20.2)
***p* values (Chi-square test)**			0.377	
**Father's occupation**				
Currently not working	63	87.0 (76.2-93.3)	10	13.0 (6.7-23.8)
Manual labourer	4347	79.5 (78.3-80.6)	1111	20.5 (19.4-21.7)
Professional	414	89.0 (85.6-91.6)	58	11.0 (8.4-14.4)
Businessman	1381	84.3 (82.2-86.3)	277	15.7 (13.7-17.8)
***p* values (Chi-square test)**			<0.001	
**Source of water**				
Improved	5399	81.1 (80.0-82.2)	1259	18.9 (17.8-20.0)
Unimproved	806	80.9 (78.2-83.3)	197	19.1 (16.7-21.8)
***p* values (Chi-square test)**			0.857	
**Type of toilet facility**				
Improved	3583	82.3 (80.9-83.6)	776	17.7 (16.4-19.1)
Unimproved	2622	79.5 (78.0-81.0)	680	20.5 (19.0-22.0)

For peer review only

			0.006	
**Solid waste use in cooking**				
No	1846	83.2 (81.3-84.9)	372	16.8 (15.1-18.7)
Yes	4359	80.2 (79.0-81.4)	1084	19.8 (18.6-21.0)
***p* values (Chi-square test)**			0.008	
**Mass media exposure**				
No	2175	78.8 (77.0-80.4)	596	21.2 (19.6-23.0)
Yes	4030	82.4 (81.0-83.6)	860	17.6 (16.4,19.0)
***p* values (Chi-square test)**			0.0009	
**Wealth index**				
Poorest	1324	77.9 (75.7-79.9)	384	22.1 (20.1-24.3)
Poorer	1213	79.0 (76.7-81.2)	332	21.0 (18.8-23.3)
Middle	1136	82.3 (80.1-84.4)	245	17.7 (15.6-19.9)
Richer	1246	81.2 (78.9-83.3)	287	18.8 (16.7-21.1)
Richest	1286	85.8 (83.5-87.9)	208	14.2 (12.1-16.5)
***p* values (Chi-square test)**			<0.001	
**Place of residence**				
Urban	2173	84.0 (82.2-85.7)	432	16.0 (14.3-17.8)
Rural	4032	80.1 (78.8-81.3)	1024	19.9 (18.7-21.2)
***p* values (Chi-square test)**			0.0004	
**Children's age (in months)**				
0-11 months	1406	83.8 (81.9-85.6)	267	16.2 (14.4-18.1)
12-23 months	1215	77.1 (74.7-79.4)	368	22.9 (20.6-25.3)
24-35 months	1139	77.1 (74.5-79.6)	336	22.9 (20.4-25.5)
36-47 months	1137	80.7 (78.3-82.9)	280	19.3 (17.1-21.7)
48-59 months	1308	86.7 (84.6-88.5)	205	13.3 (11.5-15.4)
***p* values (Chi-square test)**			0.001	
**Sex of child**				
Male	3241	81.0 (79.6-82.4)	754	19.0 (17.6-20.4)
Female	2964	81.2 (79.7-82.6)	702	18.8 (17.4-20.3)
***p* values (Chi-square test)**			=0.863	
**Birth order**				
One	2377	81.9 (80.3-83.4)	525	18.1 (16.6-19.7)
Two	2040	81.4 (79.6-83.1)	467	18.6 (16.9-20.4)
Three	1032	79.4 (76.8-81.8)	265	20.6 (18.2-23.2)
Four and above	756	80.1 (77.3-82.6)	199	19.9 (17.4-22.7)
***p* values (Chi-square test)**			0.285	
**Low birth weight**				
No	1502	82.4 (80.2-84.3)	313	17.6 (15.7-19.8)
Yes (<2.5 kg)	260	79.7 (74.2-84.3)	66	20.3 (15.7-25.8)
Not weighted	2001	77.5 (75.8-79.1)	593	22.5 (20.9-24.2)
***p* values (Chi-square test)**			0.002	
**Currently had disease**				
No	3251	80.9 (79.5-82.3)	792	19.1 (17.7-20.5)
Yes	2954	81.3 (79.8-82.7)	664	18.7 (17.3-20.2)
***p* values (Chi-square test)**			0.699	

No undenutrition for single form includes healthy children and children with multiple concurrent forms of undernutrition
CI, confidence interval

For peer review only

For peer review only - http://bmjopen.bmj.com/site/about/guidelines.xhtml

Table 4

Risk factors of multiple concurrent forms and single form of under-5 child undernutrition (results of stepwise logistic regression with backword selection)

Factors	Multiple concurrent forms		Single form	
	AOR (95% CI)	*p* values	AOR (95% CI)	*p* values
**Parents' education** [a,b]				
Both parents were uneducated	2.03 (1.53-2.71)	<0.001	1.33 (0.95-1.85)	0.095
Only father was uneducated	1.37 (1.14-1.65)	0.001	1.35 (1.12-1.63)	0.002
Only mother was uneducated	1.57 (1.14-2.16)	0.006	1.37 (0.98-1.91)	0.067
Both parents were educated	1.00		1.00	
**Underweight mothers** [a,b]				
No	1.00		1.00	
Yes	1.91 (1.62-2.25)	<0.001	1.30 (1.09-1.55)	0.003
**Father's occupation** [a,b]				
Currently not working	1.98 (1.11-3.54)	0.021	1.11 (0.53-2.31)	0.784
Manual labourer	1.25 (1.06-1.46)	0.007	1.36 (1.16-1.59)	<0.001
Professional	0.76 (0.53-1.09)	0.139	0.71 (0.50-1.01)	0.053
Businessman	1.00		1.00	
**Wealth index** [a,b]				
Poorest	2.57 (2.05-3.23)	<0.001	1.79 (1.45-2.21)	<0.001
Poorer	2.33 (1.86-2.92)	<0.001	1.63 (1.33-2.01)	<0.001
Middle	1.83 (1.46-2.30)	<0.001	1.26 (1.02-1.55)	0.035
Richer	1.79 (1.43-2.25)	<0.001	1.42 (1.16-1.74)	0.001
Richest	1.00		1.00	
**Children's age (in months)** [a,b]				
0-11 months	1.00		1.00	
12-23 months	1.78 (1.45-2.19)	<0.001	1.76 (1.46-2.10)	<0.001
24-35 months	2.70 (2.20-3.30)	<0.001	1.94 (1.61-2.34)	<0.001
36-47 months	2.51 (2.05-3.07)	<0.001	1.54 (1.27-1.87)	<0.001
48-59 months	2.35 (1.93-2.87)	<0.001	0.95 (0.77-1.16)	0.607
**Birth order** [a,b]				
One	1.00			
Two	1.02 (0.88-1.19)	0.753		
Three	1.06 (0.88-1.27)	0.538		
Four and above	1.42 (1.17-1.73)	0.001		
**Low birth weight** [b]				
No	1.00		1.00	
Yes	3.76 (2.78-5.10)	<0.001	1.52 (1.11-2.08)	0.008

Note: baseoutocme or reference category of regression model was normal chidren (multiple concurrent forms vs normal and single form vs normal) and AOR indicates adjusted odds ratio.

a, stepwise logistic regression model for multiple concurrent forms of undernutrition includes maternal age, parents' education, mother's current working status, mother's underweight status, mother's experience IPV, father's occupation, use of solid waste in cooking, type of toilet facility, mass media exposure, wealth index, place of residence, age of children and birth order

b, stepwise logistic regression model for multiple concurrent forms of undernutrition includes maternal age, parents' education, mother's current working status mother's underweight status, status of mother's antenatal and postnatal care, mother's experience IPV, father's occupation, use of solid waste in cooking, type of toilet facility, mass media exposure, wealth index, place of residence, age of children, birth order, and birth weight status

a, stepwise logistic regression model for single form of undernutrition includes parents' education, mother's underweight status, father's occupation, use of solid waste in cooking, type of toilet facility, mass media exposure, wealth index, place of residence and age of children

b, stepwise logistic regression model for multiple concurrent forms of undernutrition includes parents' education, mother's underweight status, father's occupation, use of solid waste in cooking, type of toilet facility, mass media exposure, wealth index, place of residence, age of children and birth weight status

For peer review only - http://bmjopen.bmj.com/site/about/guidelines.xhtml

Figure 1

Sample size selection

Figure 2

Trends of the prevalence of stunting, wasting and underweight

For peer review only

For peer review only - http://bmjopen.bmj.com/site/about/guidelines.xhtml

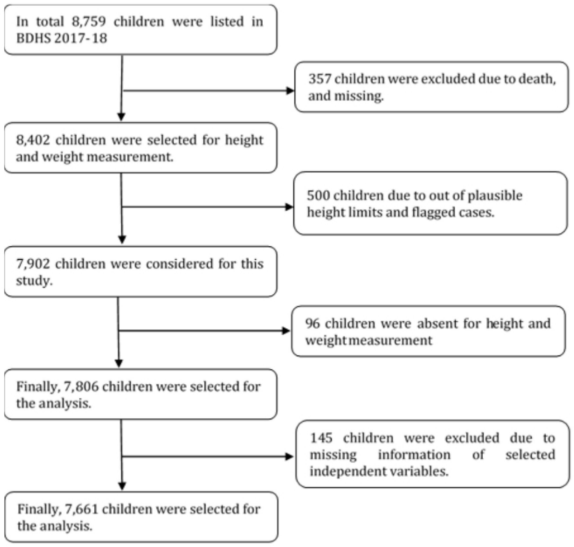

Figure 1: Sample size selection

Figure 1: Sample size selection

210x297mm (300 x 300 DPI)

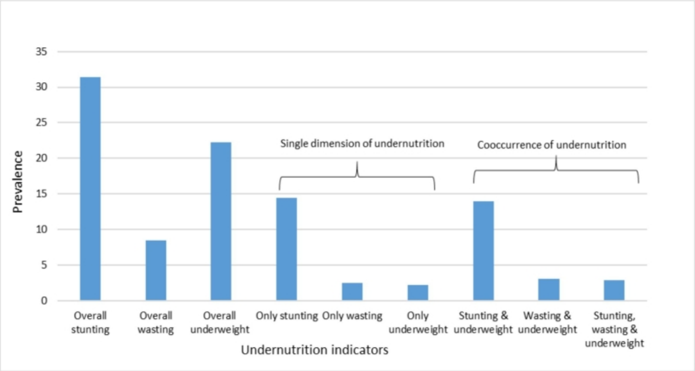

Figure 2: Prevalence of various forms of under-5 child undernutrition in Bangladesh

1

Figure 2: Prevalence of various forms of under-5 child undernutrition in Bangladesh

210x297mm (300 x 300 DPI)

Supplementary Table 1

Definitions of outcome variables

Variable	Type of variable	Description	Measurement	Scale of measurement	Number of responses/Mis sing
**Multiple concurrent forms of undernutrition**	**Outcome variable**	A child was considered to have multiple concurrent forms of undernutrition when stunting and underweight and wasting and underweight, or these three forms of undernutrition (stunting, wasting, and underweight) are present in the same child (Figure 2)	No, yes	Binary	7,806/96
**Single form of undernutrition**	**Outcome variable**	A child with a single form of undernutrition (in a composite manner) includes a child is living with stunting, wasting, or underweight only (Figure 2)	No, yes	Binary	7,806/96

For peer review only

Supplementary Table 2

Estimating scores for a child with different nutritional status

Nutritional indicators	Code	Stunting yes (1)/no (0)	wasting yes (1)/no (0)	underweight yes (1)/no (0)	Total score
**Stunning**					
No	0				
Yes	1				
**Wasting**					
No	0				
Yes	1				
**Underweight**					
No	0				
Yes	1				
**Healthy children/no undernutrition**		0	0	0	0
**Only stunting**		1	0	0	1
**Only wasting**		0	1	0	1
**Only underweight**		0	0	1	1
**Stunting & wasting**		1	0	1	2
**Stunting & underweight**		1	0	1	2
**Wasting and underweight**		0	1	1	2
**Stunting, wasting and underweight**		1	1	1	3

For peer review only

Supplementary Table 3

Variable definitions (parental characteristics)

Variable	Type of variable	Description	Measurement	Scale of measurement	Number of responses/Mis sing
**Mothers' age (in years)**	Independent variable	Age of mothers at the time of data collection	15-19, 20-24, 25-29, 30-34, 35-39, ≥ 40	Discrete, categorial	7,902/0
**Parents' education**	Independent variable	Parental educational status	Both parents were uneducated, only father was uneducated, only mother was uneducated, both parents were educated	Categorical	7,779/123
**Mother currently working**	Independent variable	Mothers engaged in economic activity at the time of data collection	No, yes	Binary	7,902/0
**Underweight mother**	Independent variable	Mother with <18.5 kg/m^2 of body mass index	No, yes	Binary	7,902/0
**Mothers received antenatal care**	Independent variable	Medical surveillance and review performed during pregnancy for the early detection of possible complications of pregnancy	No, yes	Binary	4,540/3,562
**Mothers received postnatal care**	Independent variable	Postnatal care is the individualised care provided to meet the needs of a mother and her baby following childbirth	No, yes	Binary	4,535/3,367
**Mothers' experience inmate partner violence**	Independent variable	In the BDHS surveys, IPV was measured using a shortened and modified version of the Conflict Tactics Scale and based on information such as physical violence and sexual violence. Physical violence included a wife being beaten if she went out without telling her partner, if she neglected the children and if she ever argued with her partner. The incidence of sexual violence was calculated on a woman's positive response to a question asking whether she had ever been physically forced to	No, yes	Binary	7,902/0

For peer review only

		have sexual intercourse even when she did not want to. For each of the questions, responses were re-coded dichotomously: 1= yes and 0 = no and IPV scores were calculated through adding up all four binary IPV indicators with maximum and minimum values of 4 and 0 respectively. These estimated scores were then recoded as a binary variable with categories labeled no IPV=0 and yes=1 (score 1- 4).			
**Mothers' decision-making autonomy**	Independent variable	in the BDHS surveys, a woman's decision-making power is assessed on the following three themes: 1) a woman who usually decides on her healthcare 2) a woman who usually decides on large household purchases and 3) a woman who usually decides on visits to family or relatives. The response options were as follows: (a) respondent alone, (b) respondent and husband/partner, (c) respondent and another person, (d) husband/partner alone, (e) someone else, (f) other. For each question, a value of 1 was assigned for inability in decision-making if the responses were d, e, or f and 0 for otherwise if the responses were a, b, or c. The values were then added, resulting in a score from 0 to 3. The Cronbach's α for the instruments was 0·79, indicating high internal consistency.	Not practiced, Practiced	Binary	7,778/124
**Father's occupation**	Independent variable	Fathers engaged in economic activity at the time of data collection	Currently not working, manual labourer, professional, businessman	Categorical	7,772/130

For peer review only

Supplementary Table 4

Variable definitions (household and contextual factors)

**Variable**	**Type of variable**	**Description**	**Measurement**	**Scale of measurement**	**Number of responses/Mis sing**
**Source of water**	Independent variable	improved: piped into dwelling, piped to yard/plot, public tap/standpipe, piped to neighbor, tube well or borehole, protected well, protected spring, rainwater, tanker truck, cart with small tank, bottled water; unimproved: unprotected well, unprotected spring, surface water (river, dam, lake, pond, stream, canal, irrigation channel), other	Improved, unimproved	Binary	7,902/0
**Type of toilet facility**	Independent variable	improved: flush - to piped sewer system, flush - to septic tank, flush - to pit latrine, flush - don't know where, pit latrine - ventilated improved pit, pit latrine - with slab, composting toilet; unimproved: flush - to somewhere else, pit latrine - without slab / open pit, bucket toilet, hanging toilet/latrine, other	Improved, unimproved	Binary	7,902/0
**Solid waste use in cooking**	Independent variable	solid waste includes coal, lignite, charcoal, wood, straw / shrubs / grass, agricultural crop and animal dung; unsolid include electricity, natural gas, processed gas, biogas, kerosene	No, yes	Binary	7,896/8
**Mass media exposure**	Independent variable	mass media exposure through television, radio and newspaper/magazine has been defined as exposure to at least one media that exposes to at least once a week	No, yes	Binary	7,902/0
**Wealth index**	Independent variable	wealth index in the DHS surveys is calculated, by the DHS authority, based on information on household characteristics and assets using	Poorest, poorer, middle, richer, richest	Categorical	7,902/0

For peer review only

		principal component analysis. Then households are classified into quintiles based on the values of the wealth index, where households with lower values of the index is considered as poorest and vice-versa			
**Place of residence**	Independent variable		Urban, rural	Binary	7,902/0

For peer review only

Supplementary Table 5

Variable definitions (child characteristics)

Variable	Type of variable	Description	Measurement	Scale of measurement	Number of responses/Mis sing
**Children's age (in months)**	Independent variable	Age of the children at the time of data collection	0-11 months, 12-23 months, 24-35 months, 36-47 months, 48-59 months	Categorical	7,902/0
**Sex of child**	Independent variable	Sex differential of children	Male, female	Binary	7,902/0
**Birth order**	Independent variable	Birth order is the chronological order of sibling births in a family	One, two, three, four and above	Categorical	7,902/0
**Low birth weight**	Independent variable	Children were <2.5 kg of weight during birth. Approximately 75% mothers can correctly report their baby's size at birth; therefore mother's recall is a valid proxy measure of birth weight.	No, yes, not weighted	Categorical	4,735/3,167
**Currently had disease**	Independent variable	Children had at least cough, fever or diarrhea before 2 weeks of the survey	No, yes	Binary	7,899/3

For peer review only

STROBE 2007 (v4) Statement—Checklist of items that should be included in reports of *cross-sectional studies*

Section/Topic	Item #	Recommendation	Reported on page #
**Title and abstract**	1	(*a*) Indicate the study's design with a commonly used term in the title or the abstract	2
		(*b*) Provide in the abstract an informative and balanced summary of what was done and what was found	2
**Introduction**			
Background/rationale	2	Explain the scientific background and rationale for the investigation being reported	4-6
Objectives	3	State specific objectives, including any prespecified hypotheses	5-6
**Methods**			
Study design	4	Present key elements of study design early in the paper	6-8
Setting	5	Describe the setting, locations, and relevant dates, including periods of recruitment, exposure, follow-up, and data collection	6-8
Participants	6	(*a*) Give the eligibility criteria, and the sources and methods of selection of participants	7-8
Variables	7	Clearly define all outcomes, exposures, predictors, potential confounders, and effect modifiers. Give diagnostic criteria, if applicable	9-11
Data sources/ measurement	8*	For each variable of interest, give sources of data and details of methods of assessment (measurement). Describe comparability of assessment methods if there is more than one group	11 (online supplemental table 2)
Bias	9	Describe any efforts to address potential sources of bias	8
Study size	10	Explain how the study size was arrived at	Figure 1
Quantitative variables	11	Explain how quantitative variables were handled in the analyses. If applicable, describe which groupings were chosen and why	online supplemental table 2
Statistical methods	12	(*a*) Describe all statistical methods, including those used to control for confounding	11-12
		(*b*) Describe any methods used to examine subgroups and interactions	11
		(*c*) Explain how missing data were addressed	7 (Figure 1)
		(*d*) If applicable, describe analytical methods taking account of sampling strategy	11-12
		(*e*) Describe any sensitivity analyses	11
**Results**			
Participants	13*	(a) Report numbers of individuals at each stage of study—eg numbers potentially eligible, examined for eligibility, confirmed eligible, included in the study, completing follow-up, and analysed	Page 7 (Figure 1)
		(b) Give reasons for non-participation at each stage	Page 7 (Figure 1)
		(c) Consider use of a flow diagram	Page 7 (Figure 1)

For peer review only

Descriptive data	14*	(a) Give characteristics of study participants (eg demographic, clinical, social) and information on exposures and potential confounders	12-13
		(b) Indicate number of participants with missing data for each variable of interest	online supplemental table 2
Outcome data	15*	Report numbers of outcome events or summary measures	12
Main results	16	(*a*) Give unadjusted estimates and, if applicable, confounder-adjusted estimates and their precision (eg, 95% confidence interval). Make clear which confounders were adjusted for and why they were included	12-13
		(*b*) Report category boundaries when continuous variables were categorized	online supplemental table 2
		(*c*) If relevant, consider translating estimates of relative risk into absolute risk for a meaningful time period	Not relevant
Other analyses	17	Report other analyses done—eg analyses of subgroups and interactions, and sensitivity analyses	13
**Discussion**			
Key results	18	Summarise key results with reference to study objectives	14-19
Limitations	19	Discuss limitations of the study, taking into account sources of potential bias or imprecision. Discuss both direction and magnitude of any potential bias	18-19
Interpretation	20	Give a cautious overall interpretation of results considering objectives, limitations, multiplicity of analyses, results from similar studies, and other relevant evidence	14-17
Generalisability	21	Discuss the generalisability (external validity) of the study results	19
**Other information**			
Funding	22	Give the source of funding and the role of the funders for the present study and, if applicable, for the original study on which the present article is based	20

*Give information separately for cases and controls in case-control studies and, if applicable, for exposed and unexposed groups in cohort and cross-sectional studies.

Note: An Explanation and Elaboration article discusses each checklist item and gives methodological background and published examples of transparent reporting. The STROBE checklist is best used in conjunction with this article (freely available on the Web sites of PLoS Medicine at http://www.plosmedicine.org/, Annals of Internal Medicine at http://www.annals.org/, and Epidemiology at http://www.epidem.com/). Information on the STROBE Initiative is available at www.strobe-statement.org.

For peer review only
